# Photocycle Dynamics of the Archaerhodopsin 3 Based Fluorescent Voltage Sensor QuasAr1

**DOI:** 10.3390/ijms21010160

**Published:** 2019-12-25

**Authors:** Alfons Penzkofer, Arita Silapetere, Peter Hegemann

**Affiliations:** 1 Fakultät für Physik, Universität Regensburg, Universitätsstraße 31, D-93053 Regensburg, Germany; 2 Experimentelle Biophysik, Institut für Biologie, Humboldt Universität zu Berlin, Invalidenstraße 42, D-10115 Berlin, Germany; arita.silapetere@hu-berlin.de (A.S.); hegemann@rz.hu-berlin.de (P.H.)

**Keywords:** QuasAr1, Archaerhodopsin 3, genetically encoded fluorescent voltage sensor, absorption spectroscopic characterization, fluorescence studies, photocycle dynamics, photoisomerization, deprotonation, reprotonation

## Abstract

The retinal photocycle dynamics of the fluorescent voltage sensor QuasAr1 (Archaerhodopsin 3 P60S-T80S-D95H-D106H-F161V mutant from *Halorubrum sodomense*) in pH 8 Tris buffer was studied. The samples were photoexcited to the first absorption band of the protonated retinal Schiff base (PRSB) Ret_580 (absorption maximum at λ_max_ ≈ 580 nm), and the retinal Schiff base photoisomerization and protonation state changes were followed by absorption spectra recordings during light exposure and after light exposure. Ret_580 turned out to be composed of two protonated retinal Schiff base isomers, namely Ret_580_I_ and Ret_580_II_. Photoexcitation of Ret_580_I_ resulted in barrier-involved isomerization to Ret_540 (quantum yield ≈ 0.056) and subsequent retinal proton release leading to Ret_410 deprotonated retinal Schiff base (RSB). In the dark, Ret_410 partially recovered to Ret_580_I_ and partially stabilized to irreversible Ret_400 due to apoprotein restructuring (Ret_410 lifetime ≈ 2 h). Photoexcitation of Ret_580_II_ resulted in barrier-involved isomerization to Ret_640 (quantum yield ≈ 0.00135) and subsequent deprotonation to Ret_370 (RSB). In the dark, Ret_370 partially recovered to Ret_580_II_ and partially stabilized to irreversible Ret_350 due to apoprotein restructuring (Ret_370 lifetime ≈ 10 h). Photocycle schemes and reaction coordinate diagrams for Ret_580_I_ and Ret_580_II_ were developed and photocyle parameters were determined.

## 1. Introduction

Tracking membrane potential of cells, especially neurons, using fluorescence methods is of high interest and is an active field of research (change of membrane voltage causes change of fluorescence efficiency) [1,2,3,4,5,6,7,8,9,10]. To determine membrane voltage, a variety of voltage sensitive dyes [11,12,13], genetically encoded calcium indicators (GECI) [4,14,15], and genetically encoded voltage indicators (GEVI) based on voltage sensing domains (VSD, composed of four trans-membrane helices and fused fluorescent proteins) [16,17,18,19,20] and on microbial rhodopsins (composed of seven trans-membrane α-helices with covalently bound retinal, using the intrinsic fluorescence of retinal [9,10,21,22,23] or the modified fluorescence from attached fluorescent proteins [19,23,24] or dyes [12]) are in use. Often, Förster-type resonance energy transfer (FRET) is involved in dye or fluorescent protein connection to VSDs and rhodopins [12,25].

All-optical electrophysiology in neuroscience was achieved by channelrhodopsin based optical perturbation of membrane potentials and the membrane potential readout with fluorescent voltage sensing domains [26,27,28].

Most microbial rhodopsin voltage indicators are based on Archaerhodopsin 3 (Arch) from *Halorubrum sodomense* [29] and variants thereof obtained by mutations (Arch D95N [29], Arch D95N-D106E [27], Arch D95Q-D106E [30], Archer1 (=Arch D95E-T99C) [31], Archer2 (=Arch D95E-T99C-A225M) [31], QuasAr1 (=Arch P60S-T80S-D95H-D106H-F161V) [26], QuasAr2 (=QuasAr1 H95Q) [26], QuasAr3 (=QuasAr2 K171R) [28], paQuasAr3 (=QuasAr3 V59A) [28], Archon1 (=Arch T20S-G41A-V44E-P60S-T80P-D86N-D95Q-D106H-A136T-F161V-T183I-L197I-G241Q) [32], and Archon2 (=Arch T56P-P60S-T80P-D95H-T99S-T116I-F161V-T183I-L197I-A225C) [32]). The mutations improved the fluorescence intensity dependence on membrane voltage and the membrane localization [26,28,31,32].

Here, a detailed study is presented of the photocycle dynamics of QuasAr1 (“Quality superior to Arch”) in pH 8 Tris buffer to better understand the photoexcitation and relaxation dynamics affecting the behavior of the fluorescent voltage sensor.

The analysis of the photocycle dynamics revealed that Ret_580 was composed of two protonated retinal Schiff base (PRSB) isomers, named Ret_580_I_ and Ret_580_II_, with different photocycle dynamics (different photoisomerization paths, isomerization yields, deprotonation rates, and protonation recoveries). Schemes of the photocycle were developed according to the experimental results. While the photoisomerization occurred on a ten picoseconds timescale, the protonated retinal Schiff base deprotonation to neutral retinal Schiff base (RSB) in the formed isomeric states occurred on a ten seconds timescale. The reprotonation to the original state was found to be slow, of the order of an hour for the reformation of Ret_580_I_ and of the order of ten hours for the reformation of Ret_580_II_. The slow reformation of Ret_580_I_ and Ret_580_II_ was competing with thermal apoprotein restructuring leading to RSB stabilization without reprotonation. The thermal dynamics of QuasAr1 was studied in a separate paper (apparent melting temperature determination, thermal activated ground-state protonated retinal Schiff base isomerization, deprotonation, and apoprotein restructuring) [33].

## 2. Results

The QuasAr1 samples in pH 8 Tris buffer were photoexcited to the first absorption band (protonated retinal Schiff base Ret_580) in the green-yellow-orange spectral range, and the retinal photoisomerization and protonation state changes were followed by absorption spectra recordings during light exposure and after light exposure. The temporal absorption coefficient development at fixed wavelengths was measured with high time resolution. Additionally, excitation wavelength dependent fluorescence emission quantum distributions were measured immediately after excitation light switch-off and after sample recovery in the dark (results presented in the Supplementary Materials). Emission wavelength dependent fluorescence excitation quantum distributions were also determined after sample recovery (results are shown in the Supplementary Materials).

### 2.1. Absorption Spectroscopic Photocycle Studies

QuasAr1 samples were excited with light emitting diodes LED 590 nm (excitation near absorption maximum of Ret_580) and LED 530 nm (excitation near absorption maximum of protonated retinal Schiff base photoisomer Ret_540 of Ret_580_I_) as well as with a HeNe laser at 632.8 nm (excitation near absorption maximum of protonated retinal Schiff base photoisomer Ret_640 of Ret_580_II_). For the excitation with LED 590 nm, photocycle studies with three different excitation intensities were carried out to study the dependence of the photocycle dynamics on the excitation intensity. The excitations with LED530 nm and a HeNe laser were carried out to study the influence of the excitation wavelength within the broad S_0_–S_1_ absorption band of the Ret_580 chromophores and of the formed photoisomer excitations on the photocycle dynamics.

The results of the photocycle studies with LED 590 nm at high excitation intensity are presented below (Figure 1, Figure 2, Figure 3 and Figure 4), while the results of the photocycle studies with LED 590 nm at medium intensity (Figures S1–S3) and at low intensity (Figures S4 and S5) as well as the results of the photocycle studies with LED 530 nm (Figures S6–S9) and with the HeNe laser (Figures S10–S13) are presented in the Supplementary Materials.

**Figure 1:**
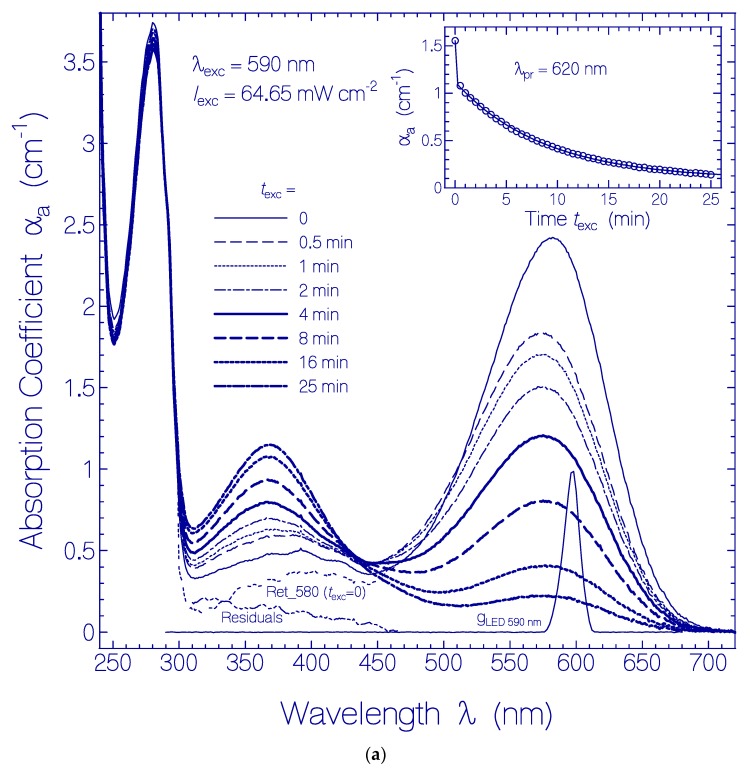

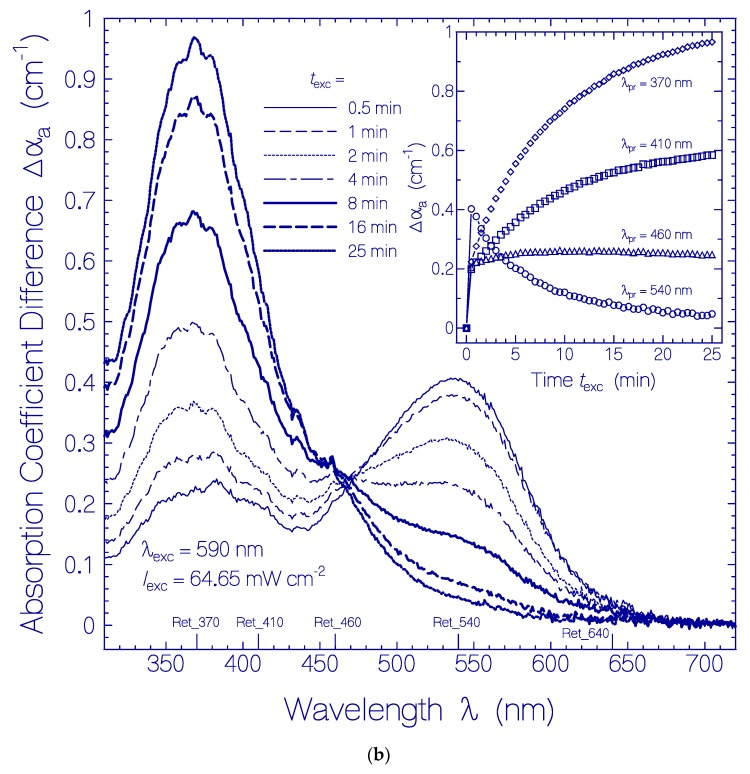
(**a**) Development of absorption coefficient spectra of a QuasAr1 sample in pH 8 Tris buffer during light exposure with LED 590 nm with input excitation intensity of *I*_exc_ = 64.65 mW cm^−2^. The durations of light exposure are listed in the figure. The triple dotted curve named Ret_580 (*t*_exc_ = 0) in the wavelength range >310 nm shows the absorption coefficient contribution of Ret_580 to QuasAr1 before light exposure (taken from Figure 1 in [33]). The dashed triple-dotted curve named Residuals shows the absorption coefficient contribution of residual retinal isomers in QuasAr1 other than Ret_580 (taken from Figure 1 in [33]). The curve g_LED 590 nm_ (λ) = *S*_LED 590 nm_ (λ)/*S*_LED 590 nm_ (λ_max_) shows the spectral distribution of the excitation light source LED 590 nm. The inset shows the temporal dependence of α_a_ (620 nm) versus exposure time *t*_exc_. The data points are fitted by $\alpha_{a}(t_{rec})=\alpha_{a}(0)-\Delta \alpha_{I}\left[1-\exp\left(-t_{exc}/\tau_{sat,I}\right)\right]-\Delta \alpha_{II}\left[1-\exp\left(-t_{exc}/\tau_{sat,II}\right)\right]$ with α_a_(0) = 1.554 cm^−1^, Δα_I_ = 0.436 cm^−1^, τ_sat,I_ = 0.015 min, Δα_II_ = 1.03 cm^−1^, and τ_sat,II_ = 8.65 min. (**b**) Absorption coefficient spectra of formed species of QuasAr1 in pH 8 Tris buffer due to light exposure with LED 590 nm of input intensity *I*_exc_ = 64.65 mW cm^−2^. The absorption contributions of Ret_580, α_aRet_580_ (λ, *t*_exc_), and of the initial residuals, α_a,Residuals_(λ,0), from (**a**) are subtracted, i.e., $\Delta \alpha_{a}(\lambda ,t_{exc})=\alpha_{a}(\lambda ,t_{exc})-\alpha_{a,\mathrm{Ret}\_580}(\lambda ,t_{exc})-\alpha_{a,\mathrm{Residuals}}(\lambda ,t_{exc}=0)$. The approximate peak wavelength positions of the retinal isomers Ret_640, Ret_540, Ret_460, Ret_410, and Ret_370 are indicated at the bottom. The inset shows the temporal development of Δα_a_ at λ_pr_ = 540 nm, 460 nm, 410 nm, and 370 nm versus exposure time *t*_exc_.

In Figure 1a, the development of absorption coefficient spectra of QuasAr1 in pH 8 buffer during light exposure with LED 590 nm (λ_exc_ = 590 nm) of input intensity *I*_exc_ = 64.65 mW cm^−2^ is displayed. The spectral light distribution *g*_LED 590 nm_ (λ) of the LED 590 nm is included in the figure. The absorption coefficient curves belong to the exposure times listed in the legend. With increasing exposure time, the curves show the decrease of the absorption band around 580 nm and the dominant buildup of an absorption band around 370 nm. The triple-dotted curve belonging to *t*_exc_ = 0 (named Ret_580 (*t*_exc_ = 0)) shows the initial absorption coefficient spectrum of QuasAr1 deprived from retinal isomer contributions other than Ret_580 (dashed triple dotted curve named Residuals). The curves Ret_580 (*t*_exc_ = 0) and Residuals were determined in [33]. The inset in Figure 1a shows the temporal development of the absorption coefficient α_a_ (*t*_exc_) at the probe wavelength λ_pr_ = 620 nm (long-wavelength absorption region of Ret_580). It indicates an initially fast absorption decrease (photoconversion of Ret_580_I_ component) followed by a slow absorption decrease (photoconversion of Ret_580_II_ component).

More detailed information on the photoinduced retinal isomerization and deprotonation dynamics of Ret_580 during light exposure was obtained by subtracting the remaining Ret_580 absorption coefficient contributions α_a,Ret_580_(λ,*t*_exc_) at time *t*_exc_ and the initial residual retinal isomers contributions Residuals from the developing absorption coefficient spectra of Figure 1a. The remaining Ret_580 absorption coefficient contributions are approximately determined by $\alpha_{a,\mathrm{Ret}\_580}(\lambda ,t_{exc})\approx \alpha_{a,\mathrm{Ret}\_580}(\lambda ,t_{exc}=0)\times \alpha_{a}(\lambda =620\;\mathrm{nm},t_{exc})/\alpha_{a}(\lambda =620\;\mathrm{nm},t_{exc}=0)$ (the spectral shape of α_a,Ret_580_ is assumed to do not change with exposure time, the magnitude of α_a,Ret_580_(λ = 620 nm, *t*_exc_) is nearly given by the magnitude of α_a_(λ = 620 nm, *t*_exc_) of QuasAr1 since at λ = 620 nm absorption contributions from formed species are thought to be small). The resulting curves $\Delta \alpha_{a}(\lambda ,t_{exc})=\alpha_{a}(\lambda ,t_{exc})-\alpha_{a,\mathrm{Ret}\_580}(\lambda ,t_{exc})-\alpha_{a,\mathrm{Residuals}}(\lambda ,t_{exc}=0)$, which are displayed in the main part of Figure 1b, show the absorption coefficient spectra development of formed species of QuasAr1 due to the light exposure. New absorption bands are seen around λ ≈ 540 nm (PRSB Ret_540), ≈ 460 nm (PRSB Ret_460), ≈ 410 nm (RSB Ret_410), and ≈ 370 nm (RSB Ret_370). There is an indication of a new absorption band around 640 nm (PRSB Ret_640). The absorption band of Ret_540 extends out beyond λ_exc_ = 590 nm. The temporal developments of Δα_a_ at the probe wavelengths λ_pr_ = 540 nm, 460 nm, 410 nm, and 370 nm are depicted in the inset of Figure 1b. The absorption band of Ret_540 decreased with continued light exposure. It is thought that Ret_540 is formed by photoisomerization of PRSB Ret_580_I_ (likely 13-*cis* isomer in specific apoprotein conformation Apoprotein_I_) to PRSB Ret_540 (likely all-*trans* isomer in apoprotein conformation Apoprotein_I_). The decrease of Ret_540 for *t*_exc_ > 30 s is thought to be determined dominantly by deprotonation of Ret_540 to Ret_410. The absorption bands of Ret_460, Ret_410, and Ret_370 are overlapping. Ret_370 was built up during the whole time of light exposure. It is thought that Ret_580_II_ (likely all-*trans* isomer in specific apoprotein conformation Apoprotein_II_) is converted to Ret_370 (likely formed by photoisomerization of all-*trans* retinal isomer to a *cis* isomer Ret_640 in specific apoprotein conformationApoprotein_II_ and subsequent deprotonation, for details see discussion below). At λ_pr_ = 460 nm the absorption changes are dominated by the short-wavelength tail of Ret_540 and the long-wavelength tails of Ret_410 and Ret_370. The build-up of Ret_460 population is small and only indicated by a small absorption structure change around 460 nm.

**Figure 2:**
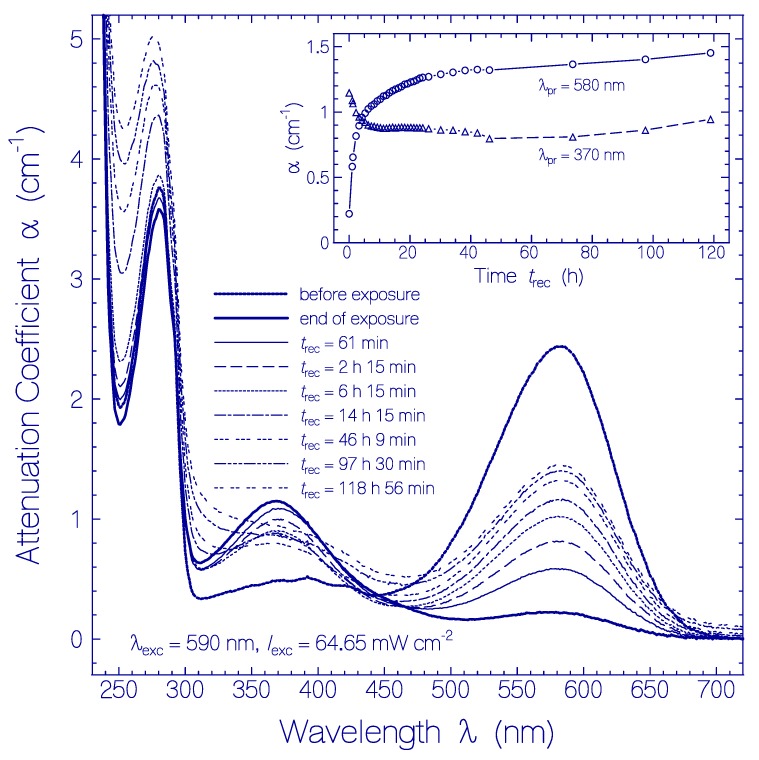
Attenuation coefficient spectra recovery of QuasAr1 in pH 8 Tris buffer after light exposure with LED 590 nm (input excitation intensity *I*_exc_ = 64.65 mW cm^−2^) for an exposure time of *t*_exc_ = 25 min (see Figure 1a). The durations of recovery *t*_rec_ are listed in the Figure. The attenuation coefficient spectra before exposure (*t*_exc_ = 0) and at end of exposure (*t*_exc_ = 25 min) are included. The inset shows the attenuation coefficient recovery α (*t*_rec_) at λ_pr_ = 580 nm and 370 nm.

The attenuation coefficient spectra development of the QuasAr1 sample used in Figure 1a after excitation light switch-off over a recovery time range of nearly five days (sample in the dark at room temperature) is displayed in Figure 2. The inset in Figure 2 shows the temporal attenuation coefficient development at λ_pr_ = 580 nm and 370 nm.

**Figure 3:**
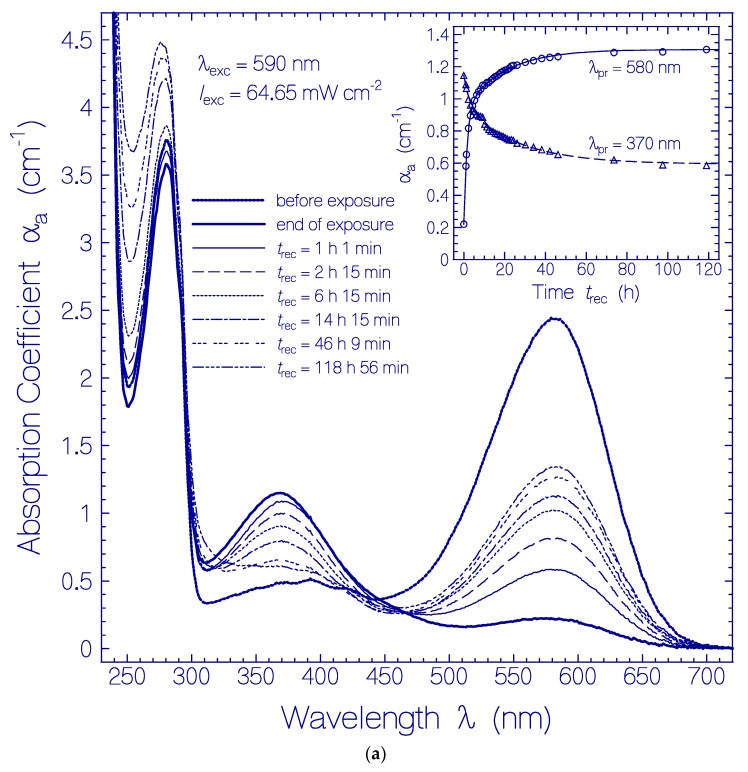

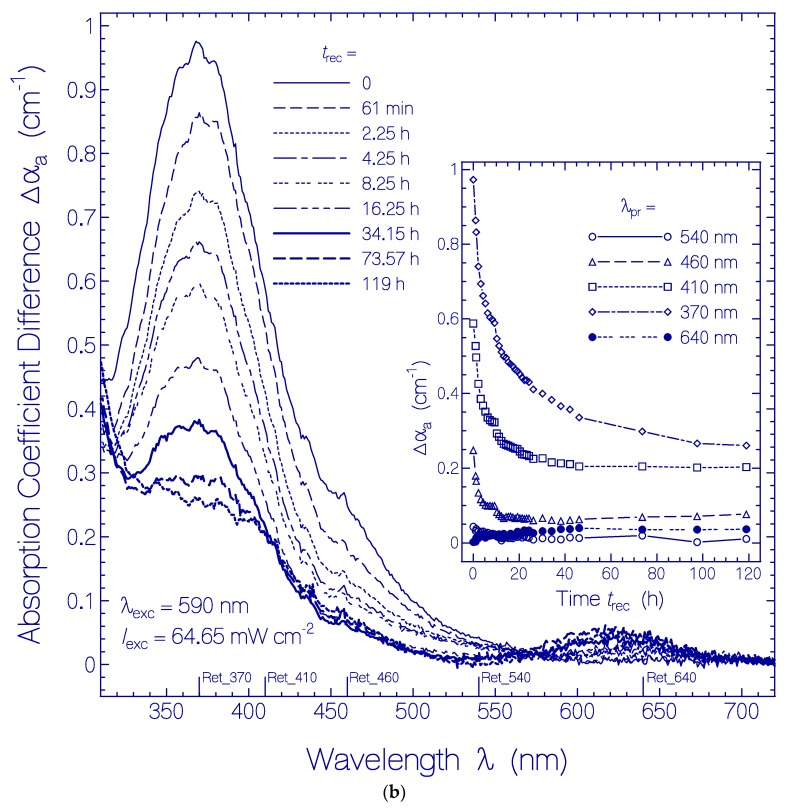
(**a**) Absorption coefficient spectra recovery of QuasAr1 in pH 8 Tris buffer after light exposure with LED 590 nm (input excitation intensity *I*_exc_ = 64.65 mW cm^−2^) for an exposure time of *t*_exc_ = 25 min (see Figure 1a). Immediately after the end of exposure, fluorescence emission spectra were measured. The durations of recovery *t*_rec_ are listed in the figure. The absorption coefficient spectra before exposure (*t*_exc_ = 0) and at the end of exposure (*t*_exc_ = 25 min) are included. The inset shows the absorption coefficient recoveries α_a_(*t*_rec_) at λ_pr_ = 580 nm and 370 nm. The data points are fitted by $\alpha_{a}(t_{rec})=\alpha_{a}(0)+\Delta \alpha_{I}\left[1-\exp\left(-t_{rec}/\tau_{rec,I}\right)\right]+\Delta \alpha_{II}\left[1-\exp\left(-t_{rec}/\tau_{rec,II}\right)\right]$ with α_a_ (0,580 nm) = 0.22 cm^−1^, Δα_I_(580 nm) = 0.71 cm^−1^, τ_rec,I_(580 nm) = 1.52 h, Δα_II_(580 nm) = 0.376 cm^−1^, τ_rec,II_(580 nm) = 19.26 h, α_a_(0,370 nm) = 1.15 cm^−1^, Δα_I_(370 nm) = −0.195 cm^−1^, τ_rec,I_(370 nm) = 2.96 h, Δα_II_(370 nm) = −0.358 cm^−1^, and τ_rec,II_(370 nm) = 26.7 h. (**b**) Absorption coefficient difference spectra development $\Delta \alpha_{a}(\lambda ,t_{rec})=\alpha_{a}(\lambda ,t_{rec})-\alpha_{a,\mathrm{Ret}\_580}(\lambda ,t_{rec})-\alpha_{a,\mathrm{Residuals}}(\lambda ,t_{exc}=0)$ of QuasAr1 in pH 8 Tris buffer after light exposure with LED 590 nm of input intensity *I*_exc_ = 64.65 mW cm^−2^ for 25 min. The inset shows the temporal development of Δα_a_ at λ_pr_ = 640 nm, 540 nm, 460 nm, 410 nm, and 370 nm versus recovery time *t*_rec_.

The corresponding absorption coefficient spectra development (scattering contributions approximately subtracted) is shown in Figure 3a. The absorption band centered at 580 nm (Ret_580) recovered partly, and the formed absorption band around 370 nm (Ret_370 including Ret_410) disappeared partly. The absorption band around 280 nm (dominant tryptophan absorption) increased steadily due to thermal apoprotein restructuring [33]. The inset in Figure 3a shows the partial absorption coefficient recovery at λ_pr_ = 580 nm were the absorption is determined by Ret_580, and the partial absorption coefficient decrease at λ_pr_ = 370 nm due to reprotonation of Ret_370 to Ret_580. The only partial reconversion of Ret_370 to Ret_580 is due to a changeover from the reversible photocycle dynamics to the thermal irreversible deprotonation of Ret_580 and the Ret_370 ground-state potential energy lowering below the ground-state energy level of Ret_580 (changeover from Ret_370 to Ret_350, see discussion below) caused by the dynamic thermal apoprotein restructuring [33] during the slow recovery time of the photocycle process.

In order to see details in the absorption coefficient spectra development after excitation light switch-off in Figure 3a, the absorption coefficient spectra development $\Delta \alpha_{a}(\lambda ,t_{rec})=\alpha_{a}(\lambda ,t_{rec})-\alpha_{a,\mathrm{Ret}\_580}(\lambda ,t_{rec})-\alpha_{a,\mathrm{Residuals}}(\lambda ,t_{exc}=0)$ is displayed in Figure 3b (Ret_580 contribution and initial residual retinal contributions are subtracted from Figure 3a). The inset of Figure 3b shows the temporal development of Δα_a_(*t*_rec_) at the probe wavelengths λ_pr_ = 540 nm, 460 nm, 410 nm, 370 nm, and 640 nm. The absorption of Ret_370, Ret_410, and Ret_460 decreased within the first 20 h of light switch-off and then leveled off. Δα_a_(640 nm,*t*_rec_) indicates the formation of Ret_640 by thermal activation of isomerization of Ret_580_II_ [33].

**Figure 4:**
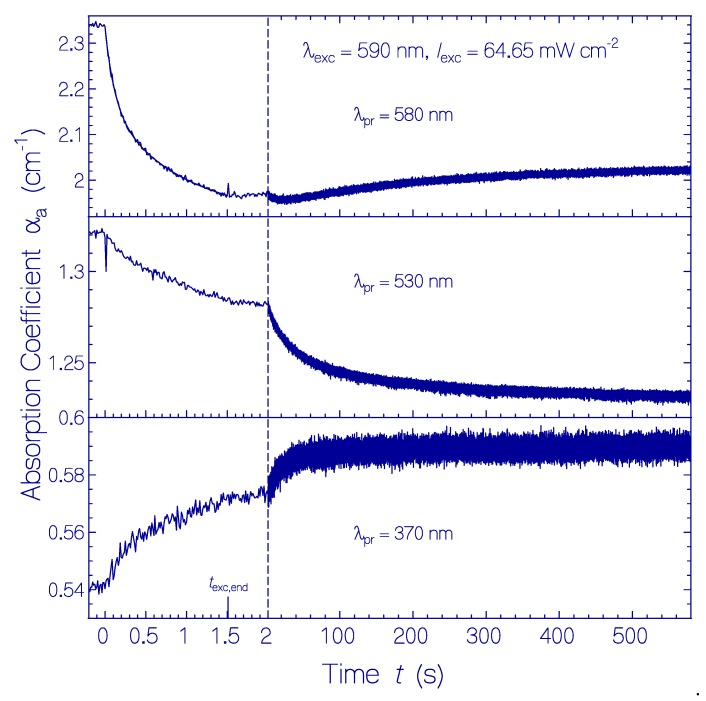
Temporal absorption coefficient development of QuasAr1 in pH 8 Tris buffer at the probe wavelengths λ_pr_ = 580 nm (top part, peak absorption of Ret_580), 530 nm (middle part, near peak absorption of Ret_540), and 370 nm (bottom part, peak absorption of Ret_370) before, during, and after photoexcitation with LED 590 nm of excitation intensity *I*_exc_ = 64.65 mW cm^−2^ for a duration of *t*_exc_ = 1.5 s. The same sample was used. Immediately after measurement at λ_pr_ = 580 nm, the measurement was continued at λ_pr_ = 530 nm, and then, at λ_pr_ = 370 nm. In the top left subfigure, the data points during light exposure are fitted by $\alpha_{a}(t_{exc})=\alpha_{a}(0)-\Delta \alpha_{a,I}\left[1-\exp\left(-t_{exc}/\tau_{I}\right)\right]-\Delta \alpha_{a,II}\left[1-\exp\left(-t_{rec}/\tau_{II}\right)\right]$ with α_a_(0) = 2.34 cm^−1^, Δα_a,I_ = 0.172 cm^−1^, τ_I_ = 116 ms, Δα_a,II_ = 0.245 cm^−1^, and τ_II_ = 907 ms. A fit of the right part of the middle subfigure with $\alpha_{a}(t>t_{exc,end})=\alpha_{a}(t_{exc,end})-\Delta \alpha_{a}\left[1-\exp\left(-\left(t-t_{exc,end}\right)/\tau_{rel,\mathrm{Ret}\_540}\right)\right]$ gives α_a_(*t*_exc,end_) = 1.284 cm^−1^, Δα_a_ = 0.0425 cm^−1^, and τ_rel,Ret_540_ = 37 s. A fit of the right part of the bottom subfigure with $\alpha_{a}(t>t_{exc,end})=\alpha_{a}(t_{exc,end})+\Delta \alpha_{a}\left[1-\exp\left(-\left(t-t_{exc,end}\right)/\tau_{rel,\mathrm{Ret}\_640}\right)\right]$ gives α_a_(*t*_exc,end_) = 0.571 cm^−1^, Δα_a_ = 0.017 cm^−1^, and τ_rel,Ret_640_ = 19.3 s.

The temporal absorption coefficient developments with a time resolution of δ*t*_res_ = 12.5 ms at λ_pr_ = 580 nm, 530 nm, and 370 nm are displayed in Figure 4 for a QuasAr1 sample in pH 8 Tris buffer. A fresh thawed sample was used. In the first run, the probe wavelength was set to λ_pr_ = 580 nm, the exposure time was *t*_exc_ = 1.5 s, and the time of recovery in the dark was set to 10 min. Then, it was followed immediately by the second run with the same exposure/dark parameters at λ_pr_ = 530 nm. Next, it was followed immediately by the third run with the same exposure/dark parameters at λ_pr_ = 370 nm.

The top part of Figure 4 shows the absorption development at λ_pr_ = 580 nm during and after light exposure. During light exposure, the absorption decreased dominantly by photoisomerization of Ret_580_I_ to Ret_540. After excitation light switch-off, initially a minute absorption decrease is observed likely due to the conversion of Ret_540 to Ret_410 (absorption band of Ret_540 extends out to 580 nm). The following slight absorption increase is thought to be due to partial reprotonation of Ret_410 to Ret_580_I_ (see discussion below).

The middle part of Figure 4 shows the absorption development at λ_pr_ = 530 nm in a second exposure of the sample. The absorption decrease during light exposure is due to the absorption decrease of the broad absorption band of Ret_580 which dominates the absorption at 530 nm. The weaker absorption decrease, as compared with λ_pr_ = 580 nm, is due to the formation of the absorption band of Ret_540 during light exposure. After light switch-off, the absorption at 530 nm decreased because of deprotonation of Ret_540 to Ret_410 (time constant τ_rel,Ret_540_ ≈ 37 s, see discussion below). The spike at the position of light switch-on is thought to be an artifact caused by a photoinduced transient thermal grating [34,35] (the same effect was observed by replacing the QuasAr1 sample with a sample of rhodamine 6G in methanol).

The bottom part of Figure 4 shows the absorption development at λ_pr_ = 370 nm in a third exposure of the sample. After excitation light switch-on, the increase of absorption is slightly time delayed (≈0.1 s). After excitation light switch-off (*t*_exc,end_ = 1.5 s), the absorption continues to increase within the first 40 s, and then, levels off (time constant τ_rel,Ret_640_ ≈ 17 s). The absorption dynamics is thought to be dominated by the conversion of Ret_640 to Ret_370 by proton release (see discussion below).

### 2.2. Quantum Yield of Photoconversion

The quantum yield of photoconversion *ϕ*_con_ of Ret_580 to other retinal isomers during light exposure is given [36] by the ratio of the number density Δ*N*_con_ of converted Ret_580 molecules to the number density Δ*n*_ph,abs_ of absorbed photons by Ret_580, i.e.,

(1) $$\phi_{con}=\frac{\Delta N_{con}}{\Delta n_{ph,abs}}$$

The number density Δ*N*_con_ is determined by

(2) $$\Delta N_{con}=N_{0}\frac{\Delta \alpha_{a}(\lambda_{pr})}{\alpha_{a,0}(\lambda_{pr})}$$

where *N*_0_ is the initial number density of Ret_580, α_a,0_(λ_pr_) is the initial absorption coefficient of Ret_580 at the probe wavelength λ_pr_, and Δα_a_(λ_pr_) is the absorption coefficient change of Ret_580 at λ_pr_ (λ_pr_ is selected at a wavelength region where practically only Ret_580 is absorbing).

The initial number density *N*_0_ of Ret_580 is given by

(3) $$N_{0}=\frac{\alpha_{a,0}(\lambda_{pr})}{\sigma_{a}(\lambda_{pr})}$$

where σ_a_(λ_pr_) is the absorption cross-section of Ret_580 at λ_pr_. It is presented in Figure S2 of the Supplementary Materials to [33].

The number density Δ*n*_ph,abs_ of absorbed photons by Ret_580 is determined by the excitation light intensity *I*_exc_ at the excitation wavelength λ_exc_, the time interval of light exposure δ*t*_exc_ and the absorption coefficient α_a_(λ_exc_) of Ret_580. It is given by

(4) $$\Delta n_{ph,abs}=\frac{I_{exc}\delta t_{exc}}{h\nu_{exc}}\alpha_{a}(\lambda_{exc})$$

where *h*ν_exc_ is the photon excitation energy (ν_exc_ = *c*_0_/λ_exc_ is the photon frequency, *c*_0_ is the speed of light in vacuum, and *h* is the Planck constant).

**Figure 5:**
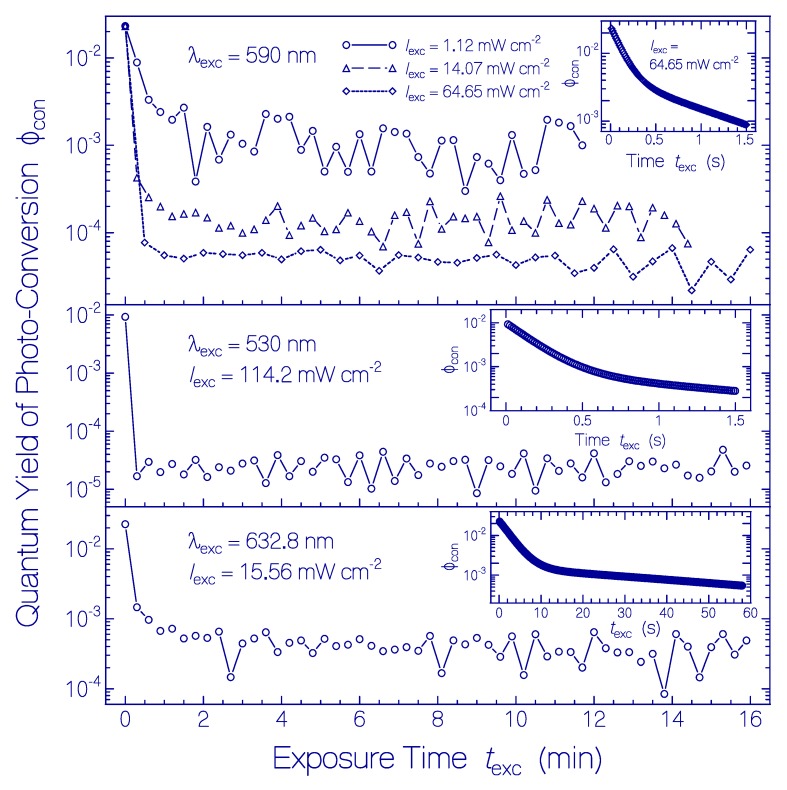
Quantum yield of photoconversion *ϕ*_con_ of Ret_580 of QuasAr1 in pH 8 Tris buffer to other retinal isomers during light exposure. (**Top part**) Photoexcitation with LED 590 nm using listed input light intensities (values derived from Figure 1a, Figures S1a and S4a). (**Middle part**) Photoexcitation with LED 530 nm using excitation intensity of *I*_exc_ = 114.2 mW cm^−2^ (values derived from Figure S6a). (**Bottom part**) Photoexcitation with He-Ne laser using *I*_exc_ = 15.56 mW cm^−2^ (values derived from Figure S10a). The curves in the insets are derived from the high time resolution absorption measurements (top part of Figure 4 for λ_exc_ = 590 nm, top part of Figure S9 for λ_exc_ = 530 nm, and top part of Figure S13 for λ_exc_ = 632.8 nm).

The determined approximate quantum yields of photoconversion of Ret_580 versus exposure time are displayed in Figure 5. The *ϕ*_con_(*t*_exc_) curves give only approximate values of *ϕ*_con,Ret_580_(*t*_exc_) since α_a_(λ_pr_,*t*_exc_) used in the calculations is only approximately equal to α_a,Ret_580_(λ_pr_,*t*_exc_), and the used α_a_(λ_exc_,*t*_exc_) is only approximately equal to α_a,Ret_580_(λ_exc_,*t*_exc_). In the main subfigures, λ_pr_ = 620 nm was used where α_a_(λ_pr_,*t*_exc_) is nearly equal to α_a,Ret_580_(λ_pr_,*t*_exc_) during the whole exposure time. In the insets of the subfigures, λ_pr_ = 580 nm was used. There, α_a_(*t*_exc_) was measured with high time resolution and for the short exposure times α_a_(λ_pr_,*t*_exc_) remained nearly equal to α_a,Ret_580_(λ_pr_,*t*_exc_). The absorption coefficient data in the insets of Figure 1a (λ_exc_ = 590 nm, *I*_exc_ = 64.65 mW cm^−2^, and λ_pr_ = 620 nm), Figure S1a (λ_exc_ = 590 nm, *I*_exc_ = 14.07 mW cm^−2^, and λ_pr_ = 620 nm), Figure S4a (λ_exc_ = 590 nm, *I*_exc_ = 1.12 mW cm^−2^, and λ_pr_ = 620 nm), Figure S6a (λ_exc_ = 530 nm, *I*_exc_ = 114.2 mW cm^−2^, and λ_pr_ = 620 nm), and Figure S10a (λ_exc_ = 632.8 nm, *I*_exc_ = 15.56 mW cm^−2^, and λ_pr_ = 620 nm) were employed for the main subfigures. The absorption coefficient curves in the top left parts of Figure 4 (λ_exc_ = 590 nm, *I*_exc_ = 64.65 mW cm^−2^, and λ_pr_ = 580 nm), Figure S9 (λ_exc_ = 530 nm, *I*_exc_ = 114.2 mW cm^−2^, and λ_pr_ = 580 nm), and Figure S13 (λ_exc_ = 632.8 nm, *I*_exc_ = 15.65 mW cm^−2^, and λ_pr_ = 580 nm) were employed for the insets in the subfigures.

All *ϕ*_con_(*t*_exc_) curves in Figure 5 show an initially fast decrease and a changeover to a near exposure time independent but excitation light intensity dependent low value. As was shown in [33] and is discussed below, Ret_580 consists of two protonated retinal Schiff base isomers Ret_580_I_ (fraction $\kappa_{{\mathrm{Ret}\_580}_{I}}$ ≈ 0.41 [33]) and Ret_580_II_ (fraction $\kappa_{{\mathrm{Ret}\_580}_{\mathrm{II}}}$ ≈ 0.59 [33]) with different ground-state isomerization dynamics [33] and photoisomerization dynamics. The initially large quantum yield of photoconversion is due to the photoisomerization of Ret_580_I_ to Ret_540 and subsequent deprotonation of Ret_540 to Ret_410. The low quantum yield of photoconversion after conversion of Ret_580_I_ is due to the low-efficient photoisomerization of Ret_580_II_ to Ret_640 and subsequent deprotonation to Ret_370. The excitation intensity dependent lowering of *ϕ*_con_(*t*_exc_) for *t*_exc_ > 0 is due to the generation of the photoisomers Ret_540 and Ret_640 and their subsequent back photoisomerization of Ret_540 to Ret_580_I_ and Ret_640 to Ret_580_II_ (see discussion below).

In the top part of Figure 5, the photoconversion of Ret_580 at λ_exc_ = 590 nm is displayed for three different excitation intensities. The initial quantum yield of photoconversion (for *t*_exc_ → 0) is excitation intensity independent. It is *ϕ*_con_(*t*_exc_ = 0) = $\phi_{con{,\mathrm{Ret}\_580}_{I}}(t_{exc}=0)\kappa_{{\mathrm{Ret}\_580}_{I}}+\phi_{con{,\mathrm{Ret}\_580}_{\mathrm{II}}}(t_{exc}=0)\kappa_{{\mathrm{Ret}\_580}_{\mathrm{II}}}$ ≈ $\phi_{{\mathrm{con},\mathrm{Ret}\_580}_{I}}(t_{exc})\kappa_{{\mathrm{Ret}\_580}_{I}}$ ≈ 0.023 giving $\phi_{{\mathrm{con},\mathrm{Ret}\_580}_{I}}(t_{exc}=0)$ ≈ 0.056. After complete photoconversion of Ret_580_I_ (*t*_exc_ > 1 min), the quantum yield of photoconversion is $\phi_{con}(t_{exc}>1\;\min,I_{exc})\approx \phi_{con,{\mathrm{Ret}\_580}_{\mathrm{II}}}(I_{exc})$ which depends on the photoexcitation intensity. We find $\phi_{con,{\mathrm{Ret}\_580}_{\mathrm{II}}}(I_{exc}=1.12\;\mathrm{mW}\;{\mathrm{cm}}^{-2})$ = (1.19 ± 0.11) × 10^−3^, $\phi_{con,{\mathrm{Ret}\_580}_{\mathrm{II}}}(I_{exc}=14.07\;\mathrm{mW}\;{\mathrm{cm}}^{-2})$ = (1.38 ± 0.084) × 10^−4^, and $\phi_{con,{\mathrm{Ret}\_580}_{\mathrm{II}}}(I_{exc}=64.65\;\mathrm{mW}\;{\mathrm{cm}}^{-2})$ = (4.53 ± 0.31) × 10^−5^. This behavior is thought to be due to the low initial quantum yield of photoconversion $\phi_{con,{\mathrm{Ret}\_580}_{\mathrm{II}}}(t_{exc}\to 0,I_{exc}\to 0)$ and the excitation intensity dependent back photoisomerization of Ret_640 to Ret_580_II_ (see discussion below).

In the middle part of Figure 5, the approximate photoconversion of Ret_580 is displayed for λ_exc_ = 530 nm and *I*_exc_ = 114.2 mW cm^−2^. The initial quantum yield of photoconversion is *ϕ*_con_(*t*_exc_ = 0) ≈ $\phi_{{\mathrm{con},\mathrm{Ret}\_580}_{I}}(t_{exc}=0)\kappa_{{\mathrm{Ret}\_580}_{I}}$ ≈ 0.0093 giving $\phi_{{\mathrm{con},\mathrm{Ret}\_580}_{I}}(t_{exc})$ ≈ 0.023. For λ_exc_ = 530 the photoconversion of Ret_580_I_ is lower than that for λ_exc_ = 590 nm indicating some excitation wavelength influence on the photoisomerization efficiency. After complete photoconversion of Ret_580_I_ (*t*_exc_ > 0.5 min) the quantum yield of photoconversion is $\phi_{con}(t_{exc}>0.5\;\min,I_{exc}=114.2\;\mathrm{mW}\;{\mathrm{cm}}^{-2})\approx \phi_{con,{\mathrm{Ret}\_580}_{\mathrm{II}}}(I_{exc}=114.2\;\mathrm{mW}\;{\mathrm{cm}}^{-2})$ = (2.43 ± 0.143) × 10^−5^, due to the excitation intensity dependent back photoisomerization of Ret_640 to Ret_580_II_ (see discussion below).

In the bottom part of Figure 5, the photoconversion of Ret_580 is displayed for λ_exc_ = 632.8 nm and *I*_exc_ = 15.56 mW cm^−2^. The initial quantum yield of photoconversion is *ϕ*_con_(*t*_exc_ = 0) ≈ $\phi_{{\mathrm{con},\mathrm{Ret}\_580}_{I}}(t_{exc}=0)\kappa_{{\mathrm{Ret}\_580}_{I}}$ = 0.023 giving $\phi_{{\mathrm{con},\mathrm{Ret}\_580}_{I}}(t_{exc}=0)$ ≈ 0.056, as in the top part of Figure 5. After complete photoconversion of Ret_580_I_ (*t*_exc_ > 1 min), the quantum yield of photoconversion is $\phi_{con}(t_{exc}>1\;\min,I_{exc}=15.56\;\mathrm{mW}\;{\mathrm{cm}}^{-2})\approx \phi_{con,{\mathrm{Ret}\_580}_{\mathrm{II}}}(I_{exc}=15.56\;\mathrm{mW}\;{\mathrm{cm}}^{-2})$ = (4.48 ± 0.3) × 10^−4^, due to the excitation intensity dependent back photoisomerization of Ret_640 to Ret_580_II_ (see discussion below).

### 2.3. Fluorescence Behavior

The excitation wavelength dependent fluorescence emission quantum distributions were measured immediately after excitation light switch-off and after sample recovery in the dark. Obtained fluorescence quantum distributions are shown in Figure S14a,b and fluorescence quantum yields are shown in Figure S15 of Section S2 of the Supplementary Materials for the QuasAr1 sample used in the photocycle experiments of Figure 1a (λ_exc_ = 590 nm, *I*_exc_ = 64.65 mW cm^−2^, and *t*_exc_ = 25 min). Immediately after photoexcitation, the fluorescence quantum efficiency in the fluorescence wavelength region of the photoconversion products turned out to be reduced. After long-time recovery in the dark at room temperature, the fluorescence behavior changed over to the fluorescence behavior of the unexposed samples stored for a long time in the dark at room temperature [33].

The emission wavelength dependent fluorescence excitation quantum distributions of photoexcited QuasAr1 samples were determined after sample recovery in the dark at room temperature. Results are shown in Figure S16 of Section S3 of the Supplementary Materials. The fluorescence excitation spectra behaved similar to the unexposed samples stored for a long time in the dark at room temperature.

## 3. Discussion

The absorption and emission spectroscopic investigation of the thermal dynamics of the Archaerhodopsin 3 based fluorescent voltage sensor QuasAr1 [33] revealed that fresh thawed samples contained, as covalently bound chromophore, dominantly protonated retinal Schiff base (PRSB) Ret_580 (absorption maximum around 580 nm) with minor amounts of a PRSB isomer absorbing around 450 nm and deprotonated retinal Schiff base (RSB) isomers absorbing below 420 nm. Ret_580 was found to be composed of two isomers, Ret_580_I_ of mole fraction $\kappa_{{\mathrm{Ret}\_580}_{I}}$ ≈ 0.41 (likely having the 13-*cis* conformation in a specific Apoprotein_I_ structure) and Ret_580_II_ of mole fraction $\kappa_{{\mathrm{Ret}\_580}_{\mathrm{II}}}$ ≈ 0.59 (likely having the all-*trans* conformation in a specific Apoprotein_II_ structure). The photocycle dynamics of Ret_580 were studied experimentally above in Section 2 and in Section S1 of the Supplementary Materials by observing the absorption spectra development during light exposure and after light exposure. The light excitation wavelength and the light excitation intensity were varied.

From the experimental results, we try to resolve the photocycle dynamics of Ret_580_I_ and Ret_580_II_ and to extract photocycle parameters in the following: The photoexcitation dynamics and the recovery dynamics of Ret_580_I_ were faster than the photoexcitation dynamics and the recovery dynamics of Ret_580_II_. These dynamics differences allow the separate characterization of the photocycle dynamics of Ret_580_I_ and Ret_580_II_.

Generally, the photoexcitation of rhodopsins causes retinal spatial *cis-trans* isomerization [37,38]. In the rhodopsin photocycle, the photoisomerization of protonated retinal Schiff base (PRSB) is followed by deprotonation to neutral retinal Schiff base (RSB), and the cycle is closed by reprotonation and back isomerization [37,38,39,40,41,42].

### 3.1. Photocycle Dynamics of Ret_580_I_

**Figure 6:**
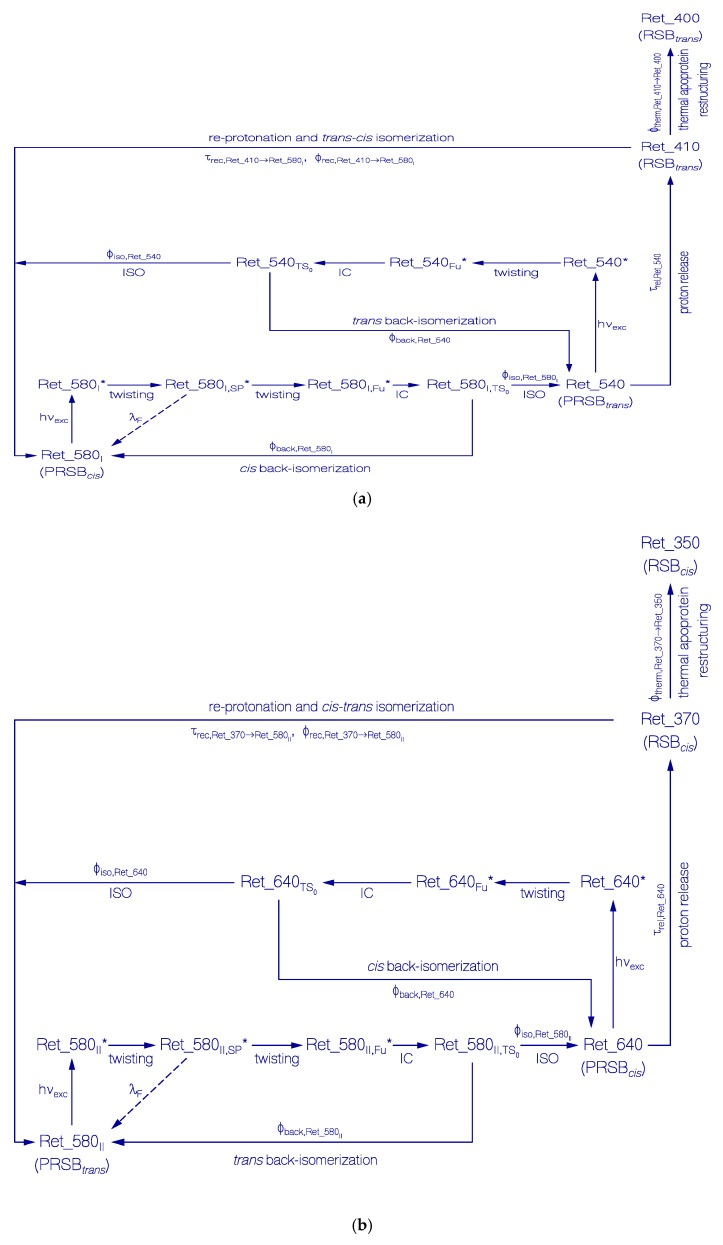
Schemes of photocycle dynamics of retinal components Ret_580_I_ (**a**) and Ret_580_II_ (**b**) of QuasAr1 in pH 8 Tris buffer. IC, internal conversion and ISO, isomerization.

**Figure 7:**
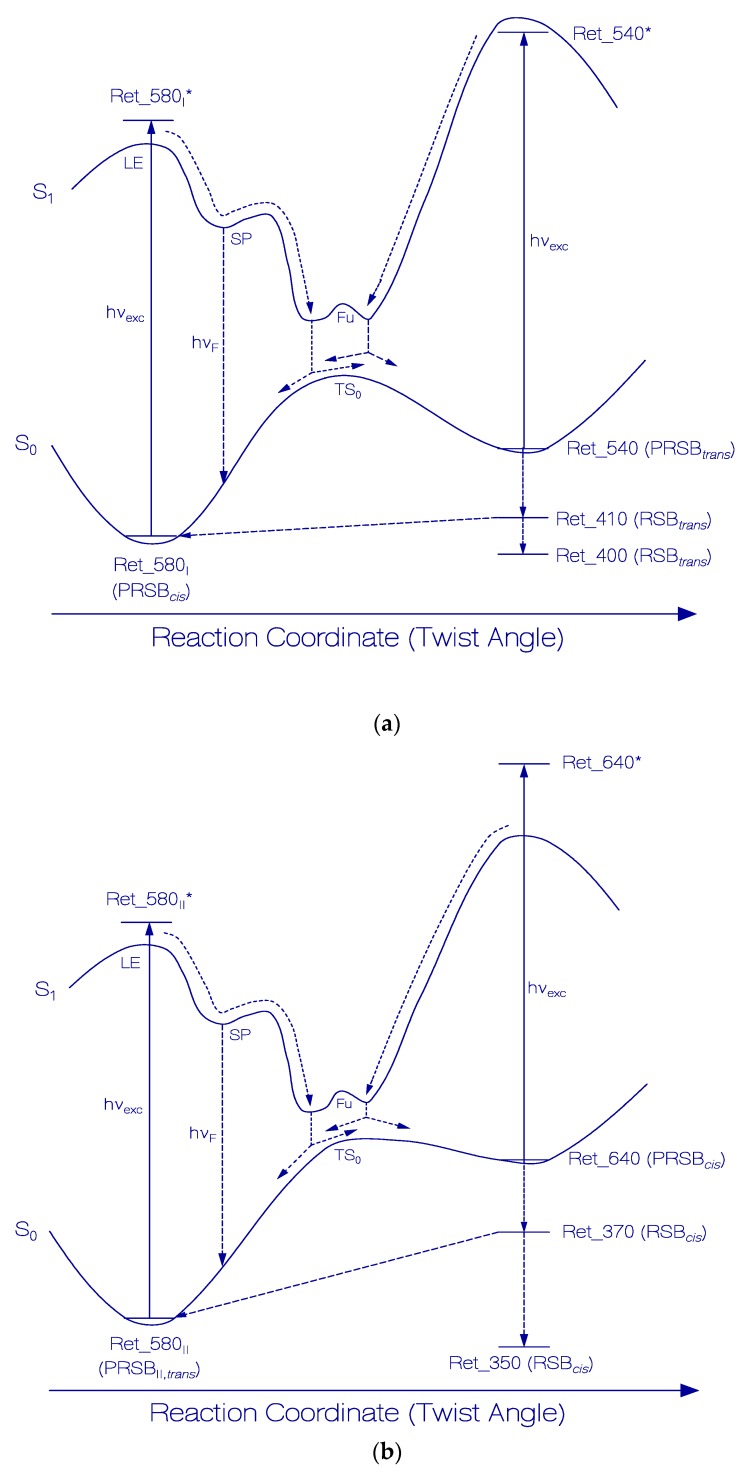
Schematic reaction coordinate diagrams for Ret_580_I_ (**a**) and Ret_580_II_ (**b**) photocycles of QuasAr1 in pH 8 Tris buffer.

In Figure 6a, a proposed scheme of the photocycle dynamics of the PRSB component Ret_580_I_ is shown, and in Figure 7a the corresponding schematic reaction coordinate diagram is depicted. Light absorption excites Ret_580_I_ in its S_0_ ground state (likely PRSB*_cis_*) to a local excited state position LE in the S_1_ first excited state (Ret_580_I_*). From there, the S_1_ state *cis-trans* isomerization begins along a torsional reaction coordinate via the stationary point SP (Ret_580_I,SP_*), and the funnel state Fu (Ret_580_I,Fu_*) with S_1_–S_0_ internal conversion (IC) to the S_0_ transition state TS_0_ (${\mathrm{Ret}\_580}_{{I,\mathrm{TS}}_{0}}$) and further torsion towards the ground-state isomer Ret_540 (likely PRSB*_trans_*). At the TS_0_ transition state, there occurs forward *trans* isomerization to Ret_540 with quantum yield of $\phi_{i{\mathrm{so},\mathrm{Ret}\_580}_{I}}$ and *cis* back isomerization with quantum yield $\phi_{back{,\mathrm{Ret}\_580}_{I}}=1-\phi_{i{\mathrm{so},\mathrm{Ret}\_580}_{I}}$. Continued light exposure causes Ret_540 photoisomerization with excitation to Ret_540*, S_1_ state twisting to Ret_540_Fu_*, S_1_–S_0_ internal conversion IC to ${\mathrm{Ret}\_580}_{{\mathrm{TS}}_{0}}$, forward isomerization to Ret_580_I_ (quantum yield $\phi_{i\mathrm{so},\mathrm{Ret}\_540}$) and back isomerization to Ret_540 (quantum yield $\phi_{back,\mathrm{Ret}\_540}=1-\phi_{i\mathrm{so},\mathrm{Ret}\_540}$). Ret_540 (PRSB*_trans_*) deprotonates to Ret_410 (RSB*_trans_*) with a relaxation time constant of τ_rel,Ret_540_. Ret_410 partly recovers back to Ret_580_I_ by reprotonation and *trans-cis* isomerization (recovery time $\tau_{rec,\mathrm{Ret}\_410\to {\mathrm{Ret}\_580}_{I}}$ and quantum yield of back recovery $\phi_{rec,\mathrm{Ret}\_410\to {\mathrm{Ret}\_580}_{I}}$) and it partly relaxes to permanently stable Ret_400 (RSB*_trans_*) caused by thermal apoprotein_I_ restructuring [33]. The quantum yield of Ret_400 formation is $\phi_{therm,\mathrm{Ret}\_410\to \mathrm{Ret}\_400}$ = $1-\phi_{rec,\mathrm{Ret}\_410\to {\mathrm{Ret}\_580}_{I}}$.

The photodynamics of Ret_580_I_ is described in Section S4.1 of the Supplementary Materials. The parameters of the Ret_580_I_ photocycle dynamics derived in the analysis are collected in Table 1.

The speed of Ret_580_I_ *cis-trans* photoisomerization to Ret_540 is slowed down by a potential energy barrier along the S_1_ state torsional path from the local excited state LE to the funnel state Fu of internal conversion. The time constant of Ret_580_I_ *cis-trans* photoisomerization to Ret_540, $\tau_{iso,{\mathrm{Ret}\_580}_{I}\to \mathrm{Ret}\_540}$ is of the order of the Ret_580 average Strickler–Berg based fluorescence lifetime [43,44,45] of τ_F,SB,Ret_580_ ≈ 61.5 ps [33] (separate fluorescence lifetimes for Ret_580_I_ and Ret_580_II_ were not determined).

The quantum yield of Ret_580_I_ → Ret_540 photoisomerization was found to be rather small and dependent on the photoexcitation wavelength ($\phi_{iso,{\mathrm{Ret}\_580}_{I}}(590\;\mathrm{nm})$ ≈ 0.056, $\phi_{iso,{\mathrm{Ret}\_580}_{I}}(530\;\mathrm{nm})$ ≈ 0.023). The S_1_–S_0_ internal conversion occurs at a reaction coordinate twist angle of less than 90° favoring the back isomerization to the original state ($\phi_{back,{\mathrm{Ret}\_580}_{I}}(590\;\mathrm{nm})=1-\phi_{iso,{\mathrm{Ret}\_580}_{I}}(590\;\mathrm{nm})$ ≈ 0.944, $\phi_{back,{\mathrm{Ret}\_580}_{I}}(530\;\mathrm{nm})=1-\phi_{iso,{\mathrm{Ret}\_580}_{I}}(530\;\mathrm{nm})$ ≈ 0.977).

The metastable Ret_540 lifetime was found to be τ_rel,Ret_540_ = 39 ± 3 s. Ret_540 deprotonates to Ret_410. During light exposure, the population of Ret_540 accumulates and the light exposure causes photoexcitation and photoisomerization of Ret_540. The data analysis (Section S.4.1 of Supplementary Materials) gives a quantum yield of Ret_540 forward photoisomerization to Ret_580_I_ of $\phi_{iso,\mathrm{Ret}\_540}(590\;\mathrm{nm})$ ≈ 0.21 and $\phi_{iso,\mathrm{Ret}\_540}(530\;\mathrm{nm})$ ≈ 0.125. The S_1_–S_0_ internal conversion occurs at a reaction coordinate twist angle of slightly larger than 90° favoring the back isomerization to the original Ret_540 state ($\phi_{back,\mathrm{Ret}\_540}(590\;\mathrm{nm})=1-\phi_{iso,\mathrm{Ret}\_540}(590\;\mathrm{nm})$ ≈ 0.79, $\phi_{back,\mathrm{Ret}\_540}(530\;\mathrm{nm})=1-\phi_{iso,\mathrm{Ret}\_540}(530\;\mathrm{nm})$ ≈ 0.875).

The lifetime $\tau_{rec,\mathrm{Ret}\_410\to {\mathrm{Ret}\_580}_{I}}$ of the deprotonated retinal Schiff base Ret_410 after excitation light switch-off depended somewhat on the previous excitation light conditions ($\tau_{rec,\mathrm{Ret}\_410\to {\mathrm{Ret}\_580}_{I}}$ (λ_exc_ = 632.8 nm and *I*_exc_ = 15.65 mW cm^−2^) ≈ 2.6 h, $\tau_{rec,\mathrm{Ret}\_410\to {\mathrm{Ret}\_580}_{I}}$ (λ_exc_ = 530 nm and *I*_exc_ = 114.2 mW cm^−2^) ≈ 0.9 h). Ret_410 recovers partly back to Ret_580_I_ by reprotonation and *trans-cis* back isomerization ($\phi_{rec,\mathrm{Ret}\_410\to {\mathrm{Ret}\_580}_{I}}$ (λ_exc_ = 632.8 nm and *I*_exc_ = 15.65 mW cm^−2^) ≈ 0.38, $\phi_{rec,\mathrm{Ret}\_410\to {\mathrm{Ret}\_580}_{I}}$ (λ_exc_ = 530 nm and *I*_exc_ = 114.2 mW cm^−2^) ≈ 0.42). This back recovery process is limited by thermal Apoprotein_I_ restructuring, thereby lowering the energy level position of the deprotonated retinal Schiff base (RSB*_trans_*) below the energy level position of Ret_580_I_ [33] (changing of metastable Ret_410 to permanently stable Ret_400 with the quantum yields $\phi_{therm,\mathrm{Ret}\_410\to \mathrm{Ret}\_400}$ (λ_exc_ = 632.8 nm and *I*_exc_ = 15.65 mW cm^−2^) ≈ 0.62, $\phi_{therm,\mathrm{Ret}\_410\to \mathrm{Ret}\_400}$ (λ_exc_ = 530 nm and *I*_exc_ = 114.2 mW cm^−2^) ≈ 0.58).

### 3.2. Photocycle Dynamics of Ret_580_II_

In Figure 6b, a proposed scheme of the photocycle dynamics the PRSB component Ret_580_II_ is shown. In Figure 7b, the corresponding schematic reaction coordinate diagram is depicted. Light absorption excites Ret_580_II_ in its S_0_ ground state (likely PRSB*_trans_*) to a local excited state position LE in the S_1_ first excited state (Ret_580_II_ *). From there, begins the S_1_ state *trans-cis* isomerization along a torsional reaction coordinate via the stationary point SP (Ret_580_II,SP_*), and the funnel state Fu (Ret_580_II,Fu_*). It follows S_1_–S_0_ internal conversion IC to the S_0_ transition state TS_0_ (${\mathrm{Ret}\_580}_{{\mathrm{II},\mathrm{TS}}_{0}}$) and continued torsion towards the ground-state isomer Ret_640 (likely PRSB*_cis_*). At the TS_0_ transition state, there occurs forward *cis* isomerization to Ret_640 with a quantum yield of $\phi_{i{\mathrm{so},\mathrm{Ret}\_580}_{\mathrm{II}}}$ and *trans* back isomerization with quantum yield of $\phi_{back{,\mathrm{Ret}\_580}_{\mathrm{II}}}=1-\phi_{i{\mathrm{so},\mathrm{Ret}\_580}_{\mathrm{II}}}$. The continued light exposure causes Ret_640 photoisomerization with excitation to Ret_640*, S_1_ state twisting to Ret_640_Fu_*¸ S_1_–S_0_ internal conversion IC to ${\mathrm{Ret}\_640}_{{\mathrm{TS}}_{0}}$, forward isomerization to Ret_580_II_ (quantum yield $\phi_{i\mathrm{so},\mathrm{Ret}\_640}$), and back isomerization to Ret_640 (quantum yield $\phi_{back,\mathrm{Ret}\_640}=1-\phi_{i\mathrm{so},\mathrm{Ret}\_640}$). Ret_640 (PRSB*_cis_*) deprotonates to Ret_370 (RSB*_cis_*) with a relaxation time constant of τ_rel,Ret_640_. Ret_370 partly recovers back to Ret_580_II_ by reprotonation and *cis-trans* isomerization (recovery time $\tau_{rec,\mathrm{Ret}\_370\to {\mathrm{Ret}\_580}_{\mathrm{II}}}$, quantum yield $\phi_{rec,\mathrm{Ret}\_370\to {\mathrm{Ret}\_580}_{\mathrm{II}}}$), and it partly relaxes to permanently stable Ret_350 (RSB*_cis_*) caused by thermal apoprotein_II_ restructuring [33]. The quantum yield of Ret_350 formation is $\phi_{therm,\mathrm{Ret}\_370\to \mathrm{Ret}\_350}=1-\phi_{rec,\mathrm{Ret}\_370\to {\mathrm{Ret}\_580}_{\mathrm{II}}}$.

The photodynamics of Ret_580_II_ is described in Section S4.2 of the Supplementary Materials. The parameters of the Ret_580_II_ photocycle dynamics derived in the analysis there are collected in Table 1.

The speed of Ret_580_II_ *trans-cis* photoisomerization to Ret_640 slowed down by a potential energy barrier along the S_1_ state torsional path from the local excited state LE to the funnel state Fu of internal conversion. The time constant of Ret_580_II_ *trans-cis* photoisomerization to Ret_640, $\tau_{iso,{\mathrm{Ret}\_580}_{\mathrm{II}}\to \mathrm{Ret}\_640}$ is of the order of the Ret_580 average Strickler–Berg based fluorescence lifetime [43,44,45] of τ_F,SB,Ret_580_ ≈ 61.5 ps [33].

The quantum yield of Ret_580_II_ → Ret_640 photoisomerization was found to be very small ($\phi_{iso,{\mathrm{Ret}\_580}_{\mathrm{II}}}(590\;\mathrm{nm})$ ≈ 0.00135). The S_1_–S_0_ internal conversion occurs at a reaction coordinate twist angle of less than 90° favoring the back isomerization to the original state ($\phi_{back,{\mathrm{Ret}\_580}_{\mathrm{II}}}(590\;\mathrm{nm})=1-\phi_{iso,{\mathrm{Ret}\_580}_{\mathrm{II}}}(590\;\mathrm{nm})$ ≈ 0.99865).

The metastable Ret_640 lifetime was found to be τ_rel,Ret_640_ = 17 ± 3 s. Ret_640 deprotonates to Ret_370. During light exposure, Ret_640 is populated and the light exposure causes photoexcitation and photoisomerization of Ret_640. The data analysis (Section S.4.2 of Supplementary Materials) gives a quantum yield of Ret_640 forward photoisomerization to Ret_580_II_ of $\phi_{iso,\mathrm{Ret}\_640}(590\;\mathrm{nm})$ ≈ 0.12. The S_1_–S_0_ internal conversion occurs at a reaction coordinate twist angle of slightly larger than 90° favoring the back isomerization to the original Ret_640 state ($\phi_{back,\mathrm{Ret}\_640}(590\;\mathrm{nm})=1-\phi_{iso,\mathrm{Ret}\_640}(590\;\mathrm{nm})$ ≈ 0.88).

The lifetime $\tau_{rec,\mathrm{Ret}\_370\to {\mathrm{Ret}\_580}_{\mathrm{II}}}$ of the deprotonated retinal Schiff base Ret_370 after excitation light switch-off depended somewhat on the previous excitation light conditions ($\tau_{rec,\mathrm{Ret}\_370\to {\mathrm{Ret}\_580}_{\mathrm{II}}}$ (λ_exc_ = 632.8 nm and *I*_exc_ = 15.65 mW cm^−2^) ≈ 15 h, $\tau_{rec,\mathrm{Ret}\_370\to {\mathrm{Ret}\_580}_{\mathrm{II}}}$ (λ_exc_ = 530 nm and *I*_exc_ = 114.2 mW cm^−2^) ≈ 8 h). Ret_370 recovers partly back to Ret_580_II_ by reprotonation and *cis-trans* back isomerization ($\phi_{rec,\mathrm{Ret}\_370\to {\mathrm{Ret}\_580}_{\mathrm{II}}}$ (λ_exc_ = 632.8 nm and *I*_exc_ = 15.65 mW cm^−2^) ≈ 0.43, $\phi_{rec,\mathrm{Ret}\_370\to {\mathrm{Ret}\_580}_{\mathrm{II}}}$ (λ_exc_ = 530 nm and *I*_exc_ = 114.2 mW cm^−2^) ≈ 0.64). This back recovery process is limited by thermal Apoprotein_II_ restructuring, thereby lowering the energy level position of the deprotonated retinal Schiff base (RSB*_cis_*) below the energy level position of Ret_580_II_ [33] (changing of metastable Ret_370 to permanently stable Ret_350 with the quantum yields $\phi_{therm,\mathrm{Ret}\_370\to \mathrm{Ret}\_350}$ (λ_exc_ = 632.8 nm and *I*_exc_ = 15.65 mW cm^−2^) ≈ 0.57, $\phi_{therm,\mathrm{Ret}\_370\to \mathrm{Ret}\_350}$ (λ_exc_ = 530 nm and *I*_exc_ = 114.2 mW cm^−2^) ≈ 0.36).

### 3.3. Comparison with Other Rhodopsins

The photocycle dynamics of QuasAr1 turned out to be slow and the quantum yield of photoizmerization was found to be low. In Table S1 of the Supplementary Materials (Section S5) quantum yields of primary photoisomerization of some rhodopsins are collected for comparization. The optimization of QuasAr1 for high fluorescence efficiency and high membrane voltage sensitivity lowered the speed of photocycle dynamics and the efficiency of photoisomerization.

## 4. Experimental

### 4.1. Sample Preparation

The sample preparation of QuasAr1 was described in [33]. The buffer contained 50 mM Tris-HCl (pH 8), 150 mM NaCl, 0.02% DDM, 0.004% CHS, 0.1 mM PMSF, and 5% glycerol. The expressed QuasAr1 solution was aliquoted to amounts of 30 μL in Eppendorf tubes, shock-frozen, and stored at −80 °C until they were thawed for experimental investigations. The experiments were carried out at room temperature.

### 4.2. Spectroscopic Measurements

Transmission measurements, *T*(λ) (λ is the wavelength), were carried out with a spectrophotometer (Cary 50, Varian Australia Pty Ltd., Mulgrave, Victoria, Australia; wavelength resolution 1.5 nm). Attenuation coefficients, α(λ) = −ln[*T*(λ)]/*l*, (*l* is sample length) were calculated, and absorption coefficients, α_a_(λ), were determined by subtracting scattering coefficient contributions, α_s_(λ), according to α_a_(λ) = α(λ)−α_s_(λ). The scattering coefficient spectrum was approximated by the empirical relation [46] $\alpha_{s}(\lambda )=\alpha_{s}(\lambda_{0}){\left(\lambda_{0}/\lambda \right)}^{\gamma }$ where the wavelength λ_0_ is selected in the transparency region and γ ≤ 4 is fitted to the experimental attenuation in the transparency region (for details see [33]).

For the absorption spectroscopic photocycle experiments, QuasAr1 samples were excited with light emitting diodes (LED 590 nm and LED 530 nm from Thorlabs Inc., Newton, NJ, United States) or with a He-Ne laser emitting at 632.8 nm (Model OEM4P, Aerotech Inc., 101 Zeta Drive, Pittsburgh, PA, USA). The sample cell in the spectrophotometer was irradiated transverse to the transmission detection path (exposed area 3 × 5 mm^2^, sample thickness along excitation path 1.5 mm, and transmission detection path length 3 mm). The excitation power *P*_exc_ was measured with a power meter (model PD 300-UV-SH photodiode detector head with NOVA power monitor, Ophir Optronics LTD., Science-based Industrial Park, Hartom St 6, Jerusalem, Israel). In the study of the absorption coefficient spectra development, transmission spectra *T*(λ) were recorded repeatedly during the period of light exposure and after light switch-off (data interval 1 nm, averaging time 0.0125 s, recording time for a spectrum from 1100 nm to 200 nm was 11.25 s, the spectra repeating time was set to 18 or 30 s during light exposure and to longer intervals in the observation of the absorption recovery after excitation light switch-off). The temporal development of the absorption behavior of QuasAr1 at selected wavelengths was carried out with a temporal resolution of 12.5 ms.

Fluorescence spectroscopic measurements immediately after the end of photoexcitation and after excitation recovery were carried out with a spectrophotometer (Cary Eclipse, Varian Australia Pty Ltd., Mulgrave, Victoria, Australia). Details of the determination of the fluorescence quantum distributions *E*_F_(λ), the fluorescence quantum yields *ϕ*_F_, and the fluorescence excitation quantum distributions *E*_ex_(λ) are given in [33]. The fluorescence spectroscopic results are presented in Sections S2 and S3 of the Supplementary Materials.

## 5. Conclusions

The photocycle dynamics of the Archaerhodopsin 3 based fluorescent voltage sensor QuasAr1 from *Halorubrum sodomense* was studied in detail. Its dominant protonated retinal Schiff base Ret_580 absorption band around 580 nm was found to consist of two isomers Ret_580_I_ (likely a *cis* isomer) and Ret_580_II_ (likely a *trans* isomer) stabilized by different adjacent apoprotein amino acid arrangements. Their slow barrier-involved photoisomerization dynamics in the tens of picosecond regime and the low quantum efficiency of photoisomerization are thought to be responsible for the high fluorescence efficiency and high membrane voltage sensitivity of QuasAr1.

The primary photoisomerization products, Ret_540 (likely PRSB*_trans_*) from the educt Ret_580_I,_ and Ret_640 (likely PRSB*_cis_*) from the educt Ret_580_II_, deprotonate slowly on a time scale of tens of seconds to the neutral retinal Schiff bases Ret_410 and Ret_370, respectively. The long lifetimes of the metastable photoisomers Ret_540 and Ret_640 cause strong excitation intensity dependent back photoisomerization to the primary isomers Ret_580_I_ and Ret_580_II_.

The reprotonation and back isomerization of the deprotonated retinal Schiff bases Ret_410 Ret_370 to the original isomers Ret_580_I_ and Ret_580_II_ occurred on a timescale of several hours. During this long time period, thermal apoprotein restructuring led to a stabilization of the deprotonated retinal Schiff base isomers, Ret_410 to Ret_400 and Ret_370 to Ret_350, leading to an incomplete recovery to the originals Ret_580_I_ and Ret_580_II_ in the photocycle process.

The performed photocycle studies on QuasAr1 are hoped to be of value for the application of this fluorescent voltage sensor in cell membrane and neuronal function studies.

## Figures and Tables

**Table 1:** Photodynamics parameters of QuasAr1 in pH 8 Tris buffer.

Parameter	Value	Comments
$\kappa_{{\mathrm{Ret}\_580}_{I}}$	≈0.41	[33]
$\kappa_{{\mathrm{Ret}\_580}_{\mathrm{II}}}$	≈0.59	[33]
λ_F,max,Ret_580_ (nm)	≈740	[33]
τ_F,SB,Ret_580_ (ps)	≈61.5	[33]
$\phi_{iso,{\mathrm{Ret}\_580}_{I}}$ (590 nm)	0.056	Figure 5 and Equation (S23)
$\phi_{iso,{\mathrm{Ret}\_580}_{I}}$ (530 nm)	0.023	Figure 5 and Equation (S23)
$\phi_{iso,{\mathrm{Ret}\_580}_{\mathrm{II}}}$ (590 nm)	0.00135	Figure 5 and Equation (S36)
$\phi_{iso,\mathrm{Ret}\_540}$ (590 nm)	≈0.21	Figure 5 and Equation (S25)
$\phi_{iso,\mathrm{Ret}\_540}$ (530 nm)	≈0.125	Figure 5 and Equation (S25)
$\phi_{iso,\mathrm{Ret}\_640}$ (590 nm)	≈0.12	Figure 5 and Equation (S37)
τ_rel,Ret_540_ (s)	39 ± 3	Middle part of Figure 4 and Figure S9
τ_rel,Ret_640_ (s)	17 ± 3	Bottom part of Figure 4 and Figure S9
$\tau_{rec,\mathrm{Ret}\_410\to {\mathrm{Ret}\_580}_{I}}$ (632.8 nm) (h)	≈2.6	Inset of Figure S12 for λ_pr_ = 580 nm (λ_exc_ = 632.8 nm and I_exc_ = 15.65 mW cm^−2^)
$\tau_{rec,\mathrm{Ret}\_410\to {\mathrm{Ret}\_580}_{I}}$ (530 nm) (h)	≈0.9	Inset of Figure S8 for λ_pr_ = 580 nm (λ_exc_ = 530 nm and I_exc_ = 114.2 mW cm^−2^)
$\tau_{rec,\mathrm{Ret}\_370\to {\mathrm{Ret}\_580}_{\mathrm{II}}}$ (632.8 nm) (h)	≈15	Inset of Figure S12 for λ_pr_ = 580 nm (λ_exc_ = 632.8 nm and I_exc_ = 15.65 mW cm^−2^)
$\tau_{rec,\mathrm{Ret}\_370\to {\mathrm{Ret}\_580}_{\mathrm{II}}}$ (530 nm) (h)	≈8	Inset of Figure S8 for λ_pr_ = 580 nm (λ_exc_ = 530 nm and I_exc_ = 114.2 mW cm^−2^)
$\phi_{rec,\mathrm{Ret}\_410\to {\mathrm{Ret}\_580}_{I}}$ (632.8 nm)	≈0.38	Figure S12 and Equation (S28)
$\phi_{rec,\mathrm{Ret}\_410\to {\mathrm{Ret}\_580}_{I}}$ (530 nm)	≈0.42	Figure S8 and Equation (S28)
$\phi_{therm,\mathrm{Ret}\_410\to \mathrm{Ret}\_400}$ (632.8 nm)	≈0.62	Figure S12 and Equation (S29)
$\phi_{therm,\mathrm{Ret}\_410\to \mathrm{Ret}\_400}$ (530 nm)	≈0.58	Figure S8 and Equation (S29)
$\phi_{rec,\mathrm{Ret}\_370\to {\mathrm{Ret}\_580}_{\mathrm{II}}}$ (632.8 nm)	≈0.43	Figure S12 and Equation (S39)
$\phi_{rec,\mathrm{Ret}\_370\to {\mathrm{Ret}\_580}_{\mathrm{II}}}$ (530 nm)	≈0.64	Figure S8 and Equation (S39)
$\phi_{therm,\mathrm{Ret}\_370\to \mathrm{Ret}\_350}$ (632.8 nm)	≈0.57	Figure S12 and Equation (S40)
$\phi_{therm,\mathrm{Ret}\_370\to \mathrm{Ret}\_350}$ (530 nm)	≈0.36	Figure S8 and Equation (S40)

Abbreviations: $\kappa_{{\mathrm{Ret}\_580}_{I}}$, fraction of Ret_580_I_ in Ret_580; $\kappa_{{\mathrm{Ret}\_580}_{\mathrm{II}}}$, fraction of Ret_580_II_ in Ret_580; λ_F,max,Ret_580_, wavelength position of maximum fluorescence emission of Ret_580; τ_F,SB,Ret_580_, Strickler-Berg based average fluorescence lifetime of Ret_580; $\phi_{iso,{\mathrm{Ret}\_580}_{I}}$, quantum yield of photoisomerization of Ret_580_I_; $\phi_{iso,{\mathrm{Ret}\_580}_{\mathrm{II}}}$, quantum yield of photoisomerization of Ret_580_II_; $\phi_{iso,\mathrm{Ret}\_540}$, quantum yield of photoisomerization of Ret_540; $\phi_{iso,\mathrm{Ret}\_640}$, quantum yield of photoisomerization of Ret_640; τ_rel,Ret_540_, relaxation time constant of Ret_540; τ_rel,Ret_640_, relaxation time constant of Ret_640; $\tau_{rec,\mathrm{Ret}\_410\to {\mathrm{Ret}\_580}_{I}}$ recovery time constant of Ret_410 to Ret_580_I_; $\tau_{rec,\mathrm{Ret}\_370\to {\mathrm{Ret}\_580}_{\mathrm{II}}}$ recovery time constant of Ret_370 to Ret_580_II_; $\phi_{rec,\mathrm{Ret}\_410\to {\mathrm{Ret}\_580}_{I}}$, quantum yield of recovery of Ret_410 to Ret_580_I_; $\phi_{rec,\mathrm{Ret}\_370\to {\mathrm{Ret}\_580}_{\mathrm{II}}}$, quantum yield of recovery of Ret_370 to Ret_580_II_; $\phi_{therm,\mathrm{Ret}\_410\to \mathrm{Ret}\_400}$, quantum yield of thermal conversion of Ret_410 to Ret_400; and $\phi_{therm,\mathrm{Ret}\_370\to \mathrm{Ret}\_350}$, quantum yield of thermal conversion of Ret_370 to Ret_350.

## Supplementary Materials

Supplementary materials can be found at https://www.mdpi.com/1422-0067/21/1/160/s1.

## Abbreviations

Arch	Archaerhodopsin 3 from *Halorubrum sodomense*
FRET	Förster resonance energy transfer
GECI	Genetically encoded calcium indicator
GEVI	Genetically encoded voltage indicator
PRSB	Protonated retinal Schiff base
QuasAr	Quality superior to Arch
Ret_xxx	Retinal with absorption maximum approximately at xxx nm
RSB	Retinal Schiff base
Trp	Tryptophan
Tyr	Tyrosine
VSD	Voltage sensing domain

## Symbols

**Symbol**	**Unit**	**Meaning**
*E*_ex_		Fluorescence excitation quantum distribution
*E′*_ex_	cm^−1^	Normalized fluorescence excitation quantum distribution
*E*_F_	nm^−1^	Fluorescence emission quantum distribtion
*I*	W cm^−2^	Intensity
*I*_exc_	W cm^−2^	Excitation intensity
*N*	cm^−3^	Number density
*g*_LED xxx nm_		Spectral light distribution of LED xxx nm
*n*_ph_	cm^−3^	Photon number density
*t*	fs, ps, ns, s, min, h, d	Time
*t*_exc_	S	Exposure time
*w*_sat_	J cm^−2^	Saturation energy density
Δ		Difference
α	cm^−1^	Attenuation coefficent
α_a_	cm^−1^	Absorption coefficent
α_s_	cm^−1^	Scattering coefficient
γ		Empirical scattering exponent
δ		Difference
ϑ	°C	Temperature
κ		Mole fraction
λ	Nm	Wavelength
λ_exc_	Nm	Excitation wavelength
λ_F_	Nm	Fluorescence emission wavelength
λ_F,exc_	Nm	Fluorescence excitation wavelength
λ_pr_	Nm	Probe wavelength
ν	Hz	Frequency
$\tilde{\nu }$	cm^−1^	Wavenumber
σ	cm^2^	Absorption cross-section
τ_F_	ps, ns	Fluorescence lifetime
τ_rec_	min, h	Recovery time constant
τ_rel_	S	Relaxation time constant
τ_sat_	S	Saturation time constant
ϕ		Quantum yield
ϕ_con_		Quantum yield of photoconversion
ϕ_F_		Fluorescence quantum yield
ϕ_iso_		Quantum yield of photoisomerization
ϕ_therm_		Quantum yielf of thermal conversion
