# Supplementary material for: Photocycle Dynamics of the Archaerhodopsin 3 Based Fluorescent Voltage Sensor QuasAr1
Source: Int J Mol Sci. 2019 Dec 25;21(1):160. doi: 10.3390/ijms21010160 (PMC6982170; doi:10.3390/ijms21010160)
Supplement: Supplementary file 1 [file ijms-21-00160-s001.pdf]

International Journal of
Molecular Sciences

MDPI

Supplementary Materials: Photocycle Dynamics of the Archaerhodopsin 3 Based Fluorescent Voltage Sensor QuasAr1

Alfons Penzkofer, Arita Silapetere and Peter Hegemann

S1. Absorption Spectroscopic Photocycle Studies

S1.1. Photoexcitation with Light Emitting Diode LED 590 nm of Medium Photoexcitation Intensity

In Figure S1a, the development of absorption coefficient spectra of a fresh thawed QuasAr1 sample in pH 8 buffer during light exposure with LED 590 nm (λ_{exc} = 590 nm) of input intensity I_{exc} = 14.07 mW cm^{-2} is displayed. The absorption coefficient curves belong to the exposure times listed in the legend. With increasing exposure time, the curves show the decrease of the absorption band around 580 nm and the dominant buildup of an absorption band around 370 nm. The inset in Figure S1a shows the temporal development of the absorption coefficient $\alpha_a(t_{exc})$ at the probe wavelength λ_{pr} = 620 nm. It indicates an initially fast absorption decrease (photoconversion of Ret_580$_I$ component) followed by a slow absorption decrease (photoconversion of Ret_580$_{II}$ component).

In Figure S1b, the absorption coefficient spectra development $\Delta\alpha_a(\lambda, t_{exc}) = \alpha_a(\lambda, t_{exc}) - \alpha_{a,\mathrm{Ret\_580}}(\lambda, t_{exc}) - \alpha_{a,\mathrm{Residuals}}(\lambda, t_{exc} = 0)$ of formed species of QuasAr1 due to the light exposure is displayed. New absorption bands are seen around λ ≈ 640 nm (PRSB Ret_640), ≈ 540 nm (PRSB Ret_540), ≈ 460 nm (PRSB Ret_460), ≈ 410 nm (RSB Ret_410), and ≈ 370 nm (RSB Ret_370). The temporal developments of $\Delta\alpha_a$ at the probe wavelengths λ_{pr} = 540 nm, 460 nm, 410 nm, and 370 nm are depicted in the inset of Figure S1b. The absorption band of Ret_540 increased within the first 54 s, then decreased for the next 10 min and leveled off. After 1 min of light exposure the absorption at 460 nm remained nearly constant. The population buildup of Ret_460 is rather small (absorption around 460 nm is dominated by absorption tails of Ret_540, Ret_410, and Ret_370). The absorptions at 410 nm and at 370 nm increased during the whole time of light exposure.

(a)

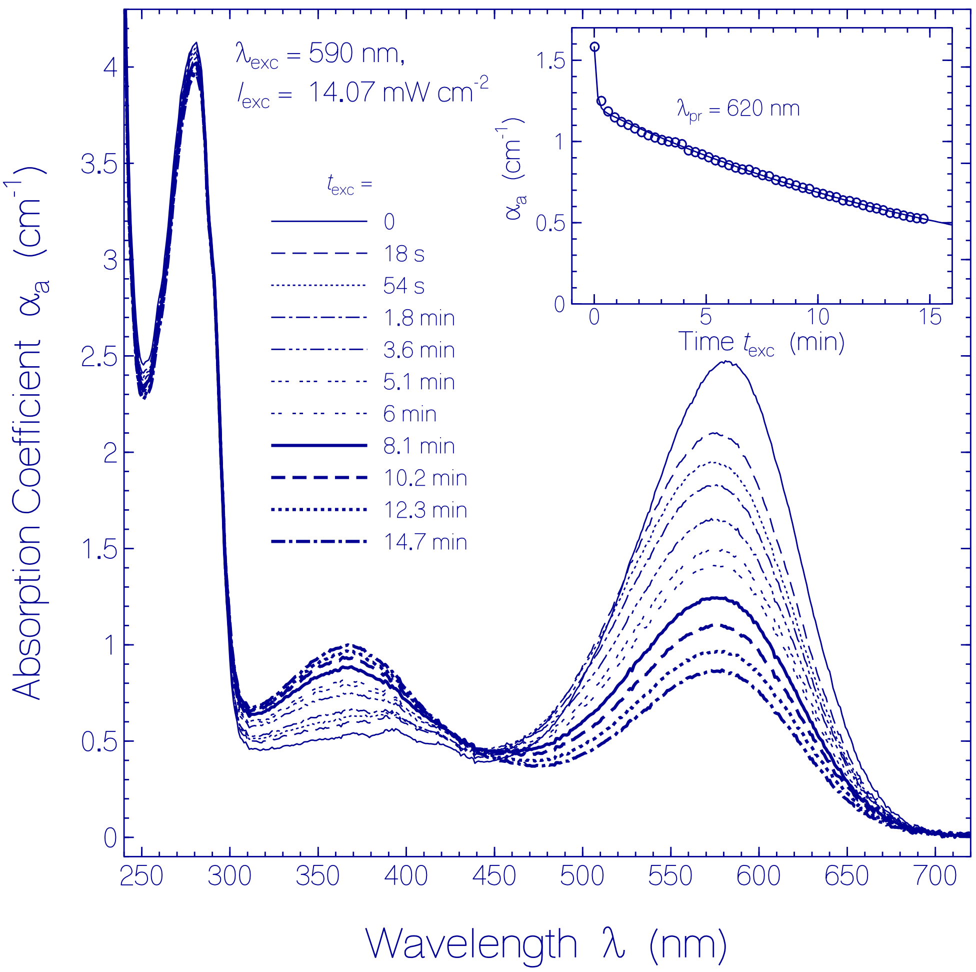
λexc = 590 nm,
Iexc = 14.07 mW cm-2
texc =
0
18 s
54 s
1.8 min
3.6 min
5.1 min
6 min
8.1 min
10.2 min
12.3 min
14.7 min
λpr = 620 nm
αa (cm-1)
Time texc (min)
Absorption Coefficient αa (cm-1)
Wavelength λ (nm)

(b)

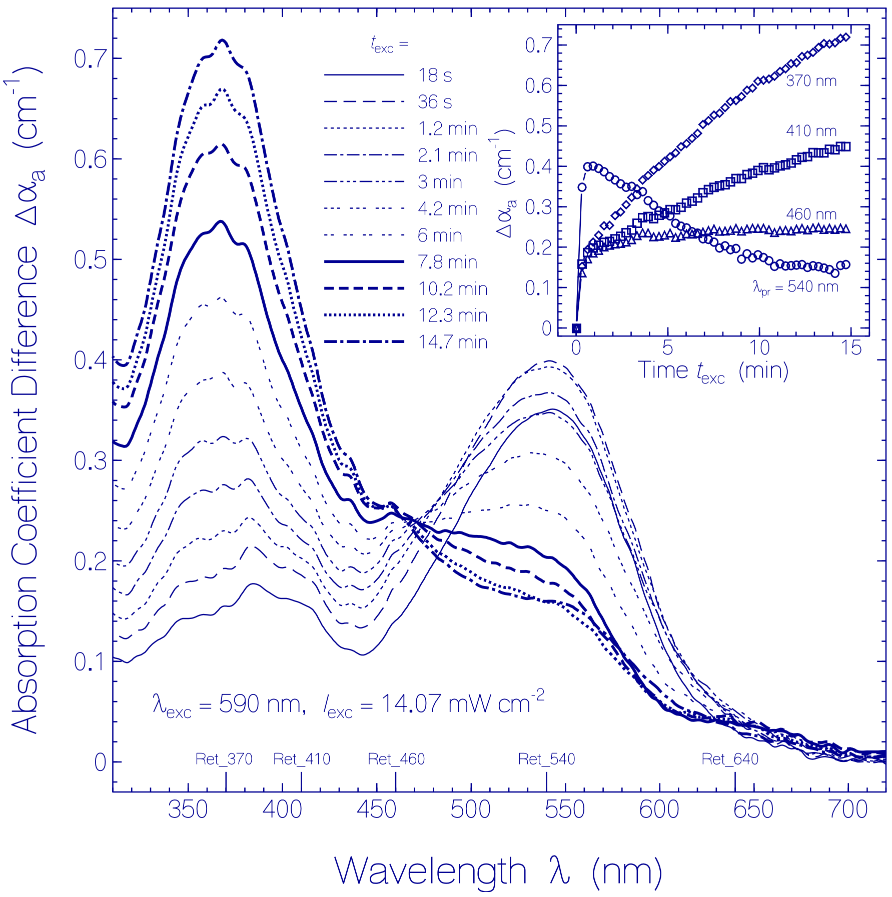
texc =
18 s
36 s
1.2 min
2.1 min
3 min
4.2 min
6 min
7.8 min
10.2 min
12.3 min
14.7 min
370 nm
410 nm
460 nm
λpr = 540 nm
Δαa (cm-1)
Time texc (min)
λexc = 590 nm, Iexc = 14.07 mW cm-2
Ret_370
Ret_410
Ret_460
Ret_540
Ret_640
Absorption Coefficient Difference Δαa (cm-1)
Wavelength λ (nm)

Figure S1. (a) Development of absorption coefficient spectra of a fresh thawed QuasAr1 sample in pH 8 Tris buffer during light exposure with LED 590 nm with input excitation intensity of I_{exc} = 14.07 mW cm^{-2}. The durations of light exposure are listed in the figure. The inset shows the temporal dependence of α_a(620 nm) versus exposure time t_{exc}. The data points are fitted by $\alpha_a(t_{rec}) = \alpha_a(0) - \Delta\alpha_I\left[1-\exp\left(-t_{exc}/\tau_{sat,I}\right)\right] - \Delta\alpha_{II}\left[1-\exp\left(-t_{exc}/\tau_{sat,II}\right)\right]$ with $\alpha_a(0)$ = 1.585 cm^{-1}, $\Delta\alpha_I$ = 0.373 cm^{-1}, $\tau_{sat,I}$ = 0.1 min, $\Delta\alpha_{II}$ = 1.22 cm^{-1}, and $\tau_{sat,II}$ = 17.78 min. **(b)** Absorption coefficient spectra of formed species of QuasAr1 in pH 8 Tris buffer due to light exposure with LED 590 nm of input intensity I_{exc} = 14.07 mW cm^{-2}. The absorption contribution of Ret_580, $\alpha_{a,Ret\_580}(\lambda,t_{exc})$, and of the initial residuals, $\alpha_{a,Residuals}(\lambda,0)$, are subtracted, i.e., $\Delta\alpha_a(\lambda,t_{exc}) = \alpha_a(\lambda,t_{exc}) - \alpha_{a,\mathrm{Ret\_580}}(\lambda,t_{exc}) - \alpha_{a,\mathrm{Residuals}}(\lambda,t_{exc}=0)$. The inset shows the temporal development of $\Delta\alpha_a$ at λ_{pr} = 540 nm, 460 nm, 410 nm, and 370 nm versus exposure time t_{exc}.

In Figure S2, the attenuation coefficient spectra development is shown after excitation light switch-off. The attenuation coefficient recovery was observed over a time period of nearly three days. The inset in Figure S2 shows the temporal attenuation coefficient development at λ_{pr} = 580 nm and 370 nm.

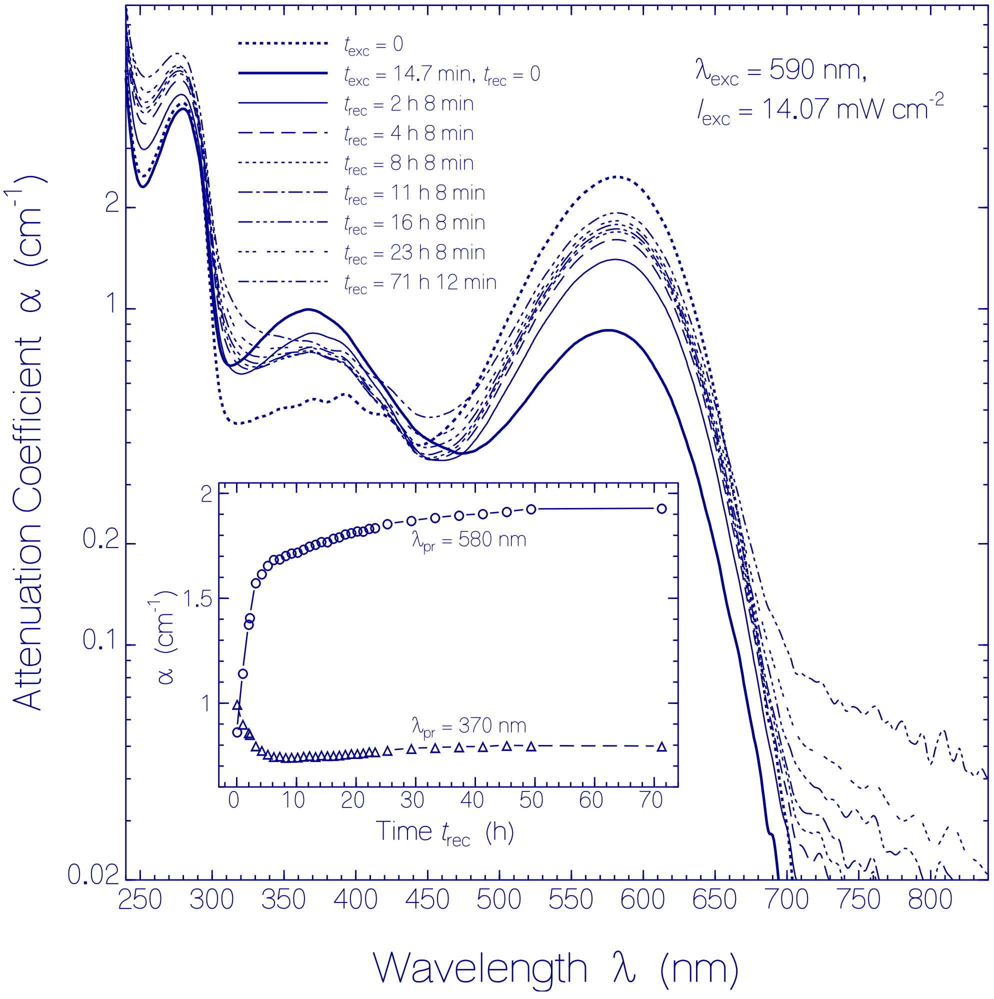

Figure S2. Attenuation coefficient spectra recovery of QuasAr1 in pH 8 Tris buffer after light exposure with LED 590 nm (input excitation intensity I_{exc} = 14.07 mW cm^{-2}) for an exposure time of t_{exc} = 14.7 min (see Figure S1a). The durations of recovery t_{rec} are listed in the figure. The attenuation coefficient spectra before exposure (t_{exc} = 0) and at end of exposure (t_{exc} = 14.7 min) are included. The inset shows the attenuation coefficient recovery $\alpha(t_{rec})$ at λ_{pr} = 580 nm and 370 nm.

The corresponding absorption coefficient spectra development is shown in Figure S3. The absorption band centered at 580 nm (Ret_580) recovered partly, and the formed absorption band around 370 nm (Ret_370 including Ret_410) disappeared partly. The attenuation band around 280 nm (dominant tryptophan absorption) increased slightly due to thermal apoprotein restructuring [33]. The inset in Figure S3 shows the partial absorption coefficient recovery at λ_{pr} = 580 nm where the absorption is determined by Ret_580 (fast rise due to recovery of Ret_410 to Ret_580$_I$, and slow rise due to recovery of Ret_370 to Ret_580$_{II}$), and the partial absorption coefficient decrease at λ_{pr} = 370 nm where the absorption is determined by Ret_410 (short-wavelength absorption tail) and Ret_370. The fast absorption decrease is caused by reprotonation of Ret_410 to Ret_580$_I$, and the slow absorption decrease is caused by reprotonation of Ret_370 to Ret_580$_{II}$, and thermal changeover from Ret_370 to irreversible Ret_350 caused by the dynamic thermal apoprotein restructuring [33].

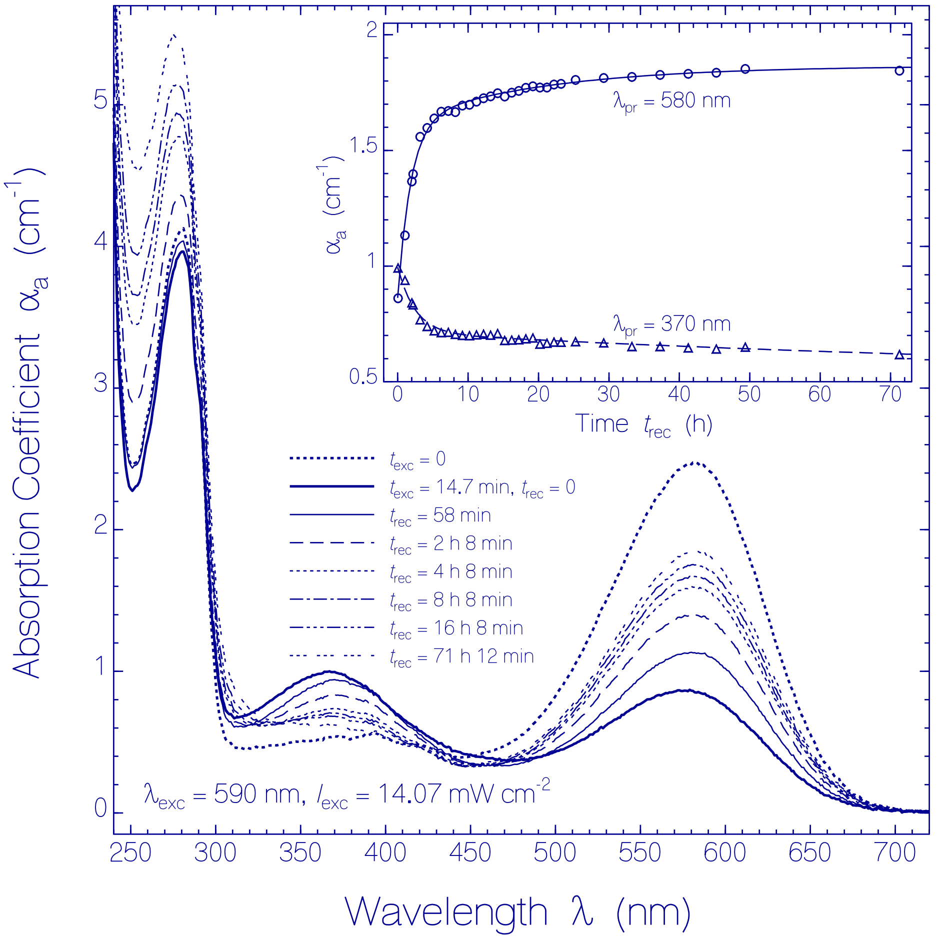

Figure S3. Absorption coefficient spectra recovery of QuasAr1 in pH 8 Tris buffer after light exposure with LED 590 nm (input excitation intensity I_{exc} = 14.07 mW cm^{-2}) for an exposure time of t_{exc} = 14.7 min (see Figure S1a). Immediately after end of exposure, fluorescence emission spectra and fluorescence excitation spectra were measured. The durations of recovery t_{rec} are listed in the figure. The absorption coefficient spectra before exposure (t_{exc} = 0) and at end of exposure (t_{exc} = 14.7 min) are included. The inset shows the absorption coefficient recovery $\alpha(t_{rec})$ at λ_{pr} = 580 nm and 370 nm. The data points are fitted by $\alpha_a(t_{rec}) = \alpha_a(0) + \Delta\alpha_{a,I}\left[1-\exp\left(-t_{rec}/\tau_{rec,I}\right)\right] + \Delta\alpha_{a,II}\left[1-\exp\left(-t_{rec}/\tau_{rec,II}\right)\right]$ with α_a(0,580 nm) = 0.86 cm^{-1}, $\Delta\alpha_{a,I}$(580 nm) = 0.76 cm^{-1}, $\tau_{rec,I}$(580 nm) = 1.86 h, $\Delta\alpha_{a,II}$(580 nm) = 0.245 cm^{-1}, $\tau_{rec,II}$(580 nm) = 20.4 h, α_a(0,370 nm) = 0.995 cm^{-1}, $\Delta\alpha_{a,I}$(370 nm) = -0.282 cm^{-1}, $\tau_{rec,I}$(370 nm) = 2.46 h, $\Delta\alpha_{a,II}$(370 nm) = -0.197 cm^{-1}, and $\tau_{rec,II}$(370 nm) = 113.7 h.

S1.2. Photoexcitation with Light Emitting Diode LED 590 nm of Low Photoexcitation Intensity

In Figure S4a, the development of absorption coefficient spectra of a fresh thawed QuasAr1 sample in pH 8 buffer during light exposure with LED 590 nm (λ_{exc} = 590 nm) of input intensity I_{exc} = 1.12 mW cm^{-2} is displayed. The absorption coefficient curves belong to the exposure times listed in the legend. With increasing exposure time, the curves show a decrease of the absorption band around 580 nm and a buildup of the absorption band around 370 nm. The inset in Figure S4a shows the temporal development of the absorption coefficient $\alpha_a(t_{exc})$ at the probe wavelength λ_{pr} = 620 nm. It indicates an initially fast absorption decrease (photoconversion of Ret_580$_I$ component) followed by a slow absorption decrease (photoconversion of Ret_580$_{II}$ component).

In Figure S4b, the absorption coefficient spectra development $\Delta\alpha_a(\lambda,t_{exc}) = \alpha_a(\lambda,t_{exc}) - \alpha_{a,\mathrm{Ret\_580}}(\lambda,t_{exc}) - \alpha_{a,\mathrm{Residuals}}(\lambda,t_{exc}=0)$ of formed species of QuasAr1 due to the light exposure is displayed. New bands are seen around λ ≈ 640 nm (PRSB Ret_640), ≈ 540 nm (PRSB Ret_540), ≈ 460 nm (PRSB Ret_460), ≈ 410 nm (RSB Ret_410), and ≈ 370 nm (RSB Ret_370). The temporal developments of $\Delta\alpha_a$ at the probe wavelengths λ_{pr} = 540 nm, 460 nm, 410 nm, and 370 nm are depicted in the inset of Figure S4b. The absorption band of Ret_540 increased within the first 2 min and then decreased slightly. After 2 min of light exposure, the absorptions at 460 nm and 410 nm continued to increase with small slope. The absorption at 370 nm increased during the whole time of light exposure with a steeper rise during the first minute of light exposure.

(a)

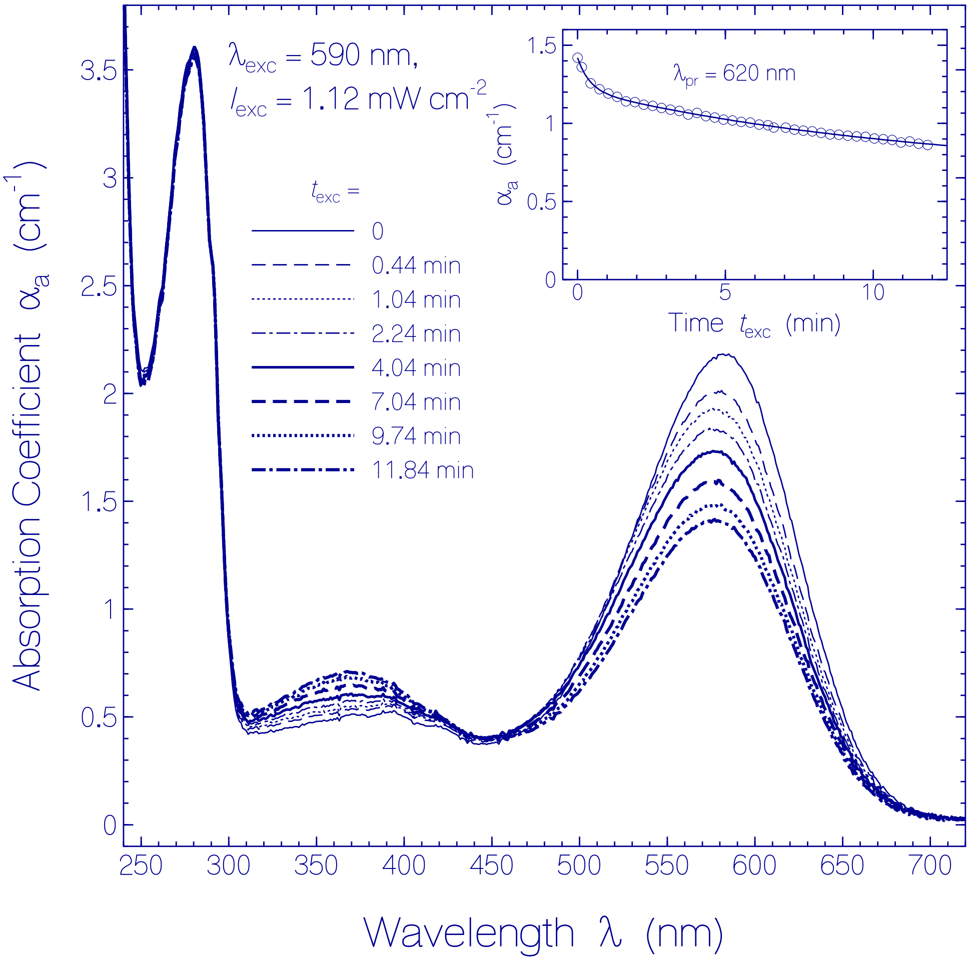

(b)

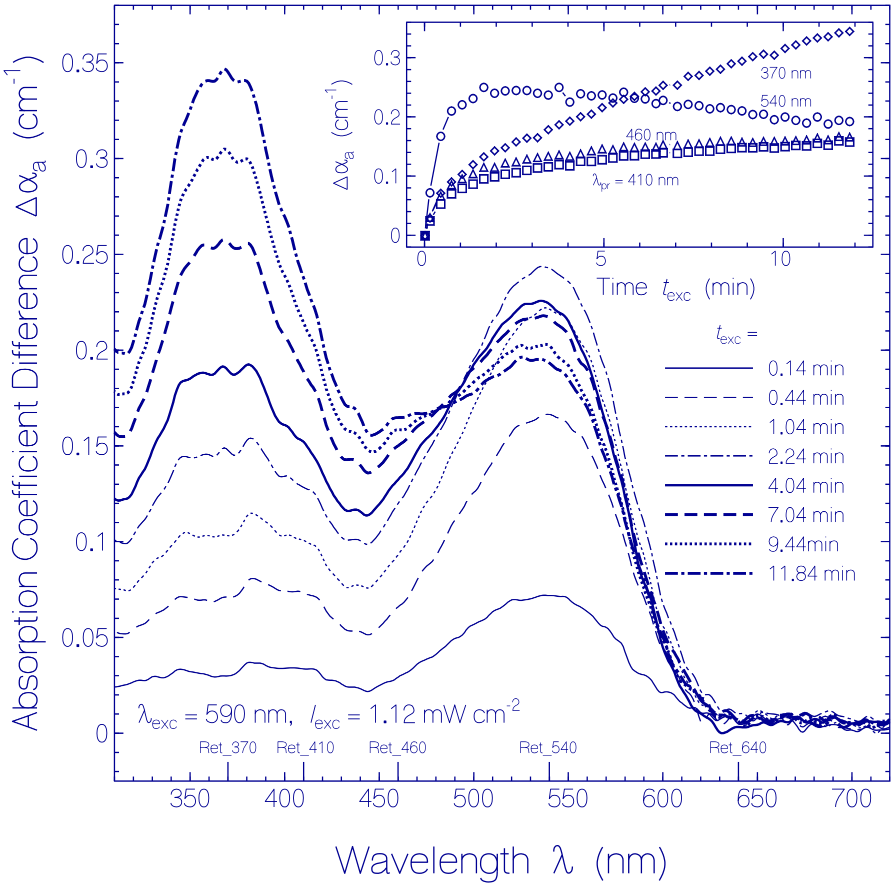

Figure S4. **(a)** Development of absorption coefficient spectra of a fresh thawed QuasAr1 sample in pH 8 Tris buffer during light exposure with LED 590 nm with input excitation intensity of I_{exc} = 1.12 mW cm^{-2}. The durations of light exposure are listed in the figure. The inset shows the temporal dependence of α_a(620 nm) versus exposure time t_{exc}. The data points are fitted by $\alpha_a(t_{rec}) = \alpha_a(0) - \Delta\alpha_I\left[1-\exp\left(-t_{exc}/\tau_{sat,I}\right)\right] - \Delta\alpha_{II}\left[1-\exp\left(-t_{exc}/\tau_{sat,II}\right)\right]$ with $\alpha_a(0)$ = 1.42 cm^{-1}, $\Delta\alpha_I$ = 0.203 cm^{-1}, $\tau_{sat,I}$ = 0.403 min, $\Delta\alpha_{II}$ = 0.546 cm^{-1}, and $\tau_{sat,II}$ = 11.67 min. **(b)** Absorption coefficient spectra of formed species of QuasAr1 in pH 8 Tris buffer due to light exposure with LED 590 nm of input intensity I_{exc} = 1.12 mW cm^{-2}. The absorption contribution of Ret_580, $\alpha_{a,\mathrm{Ret\_580}}(\lambda,t_{exc})$, and of the initial residuals, $\alpha_{a,\mathrm{Residuals}}(\lambda,0)$, are subtracted, i.e., $\Delta\alpha_a(\lambda,t_{exc}) = \alpha_a(\lambda,t_{exc}) - \alpha_{a,\mathrm{Ret\_580}}(\lambda,t_{exc}) - \alpha_{a,\mathrm{Residuals}}(\lambda,t_{exc}=0)$. The inset shows the temporal development of $\Delta\alpha_a$ at λ_{pr} = 540 nm, 460 nm, 410 nm, and 370 nm versus exposure time t_{exc}.

In Figure S5, the absorption coefficient spectra development of the QuasAr1 sample used in Figure S4a after excitation light switch-off is displayed over a recovery time range of 5.65 h (sample in the dark at room temperature). The absorption band centered at 580 nm (Ret_580) recovered partly, and the formed absorption band formed around 370 nm (Ret_370 including Ret_410) remained nearly constant. The absorption band around 280 nm (dominant tryptophan absorption) increased steadily. The inset in Figure S5 shows the partial absorption coefficient recovery at λ_{pr} = 580 nm were the absorption is determined by Ret_580 (fast rise due to recovery of Ret_410 to Ret_580$_{I}$, and slow rise due to recovery of Ret_370 to Ret_580$_{II}$), and the near constant absorption coefficient at λ_{pr} = 370 where the absorption is determined by Ret_410 (short-wavelength absorption tail), Ret_370, and the long-wavelength apoprotein absorption tail [33].

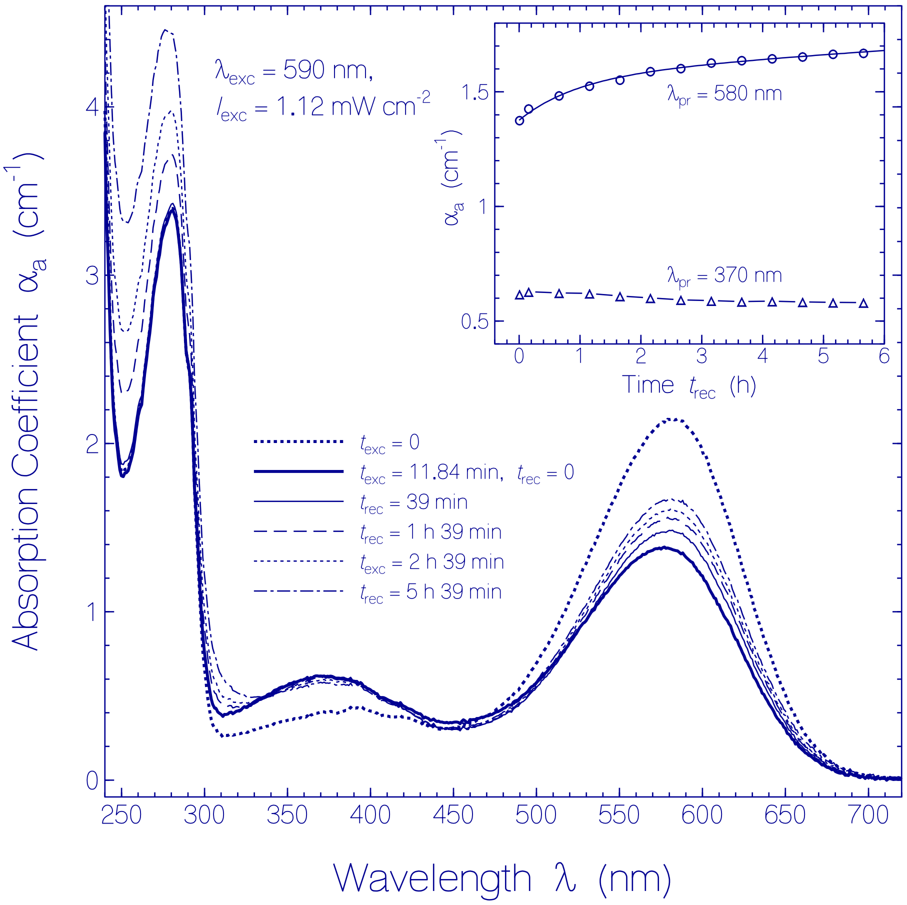

Figure S5. Absorption coefficient spectra recovery of QuasAr1 in pH 8 Tris buffer after light exposure with LED 590 nm (input excitation intensity I_{exc} = 1.12 mW cm^{-2}) for an exposure time of t_{exc} = 11.84 min (see Figure S4a). The durations of recovery t_{rec} are listed in the figure. The absorption coefficient spectra before exposure (t_{exc} = 0) and at end of exposure (t_{exc} = 11.84 min) are included. The inset shows the absorption coefficient recovery $\alpha_a(t_{rec})$ at λ_{pr} = 580 nm and 370 nm. The data points at λ_{pr} = 580 nm are fitted by $\alpha_a(t_{rec}) = \alpha_a(0) + \Delta\alpha_{a,I}\left[1-\exp\left(-t_{rec}/\tau_{rec,I}\right)\right] + \Delta\alpha_{a,II}\left[1-\exp\left(-t_{rec}/\tau_{rec,II}\right)\right]$ with $\alpha_a(0)$ = 1.38 cm^{-1}, $\Delta\alpha_{a,I}$ = 0.18 cm^{-1}, $\tau_{rec,I}$ = 0.94 h, $\Delta\alpha_{a,II}$ = 0.49 cm^{-1}, $\tau_{rec,II}$ = 20.6 h.

S1.2. Photoexcitation with Light Emitting Diode LED 530 nm

At 530 nm the absorption of a fresh thawed QuasAr1 sample is dominated by the broad absorption band of Ret_580. The photoexcitation of Ret_580 causes photoisomerization of Ret_580$_{I}$ to Ret_540 and of Ret_580$_{II}$ to Ret_640. The formed Ret_540 is photoexcited by light exposure at 530 nm and causes a partial back photoisomerization of Ret_540 to Ret_580$_{I}$ (see discussion in the main part, section 3).

In Figure S6a, the development of absorption coefficient spectra of a fresh thawed QuasAr1 sample in pH 8 buffer during light exposure with LED 530 nm (λ_{exc} = 530 nm) of input intensity I_{exc} = 114.2 mW cm^{-2} is displayed. The spectral light distribution $g_{LED\ 530\ nm}(\lambda)$ of the LED 530 nm is included in the figure. The absorption coefficient curves belong to the exposure times listed in the legend. With increasing exposure time, the curves show the decrease of the absorption band around 580 nm and the dominant buildup of an absorption band around 370 nm. The inset in Figure S6a shows the temporal development of the absorption coefficient $\alpha_a(t_{exc})$ at the probe wavelength λ_{pr} = 620 nm (long-wavelength absorption region of Ret_580). It indicates an initially fast absorption decrease (photoconversion of Ret_580$_{I}$ component) followed by a slow absorption decrease (photoconversion of Ret_580$_{II}$ component).

(a)

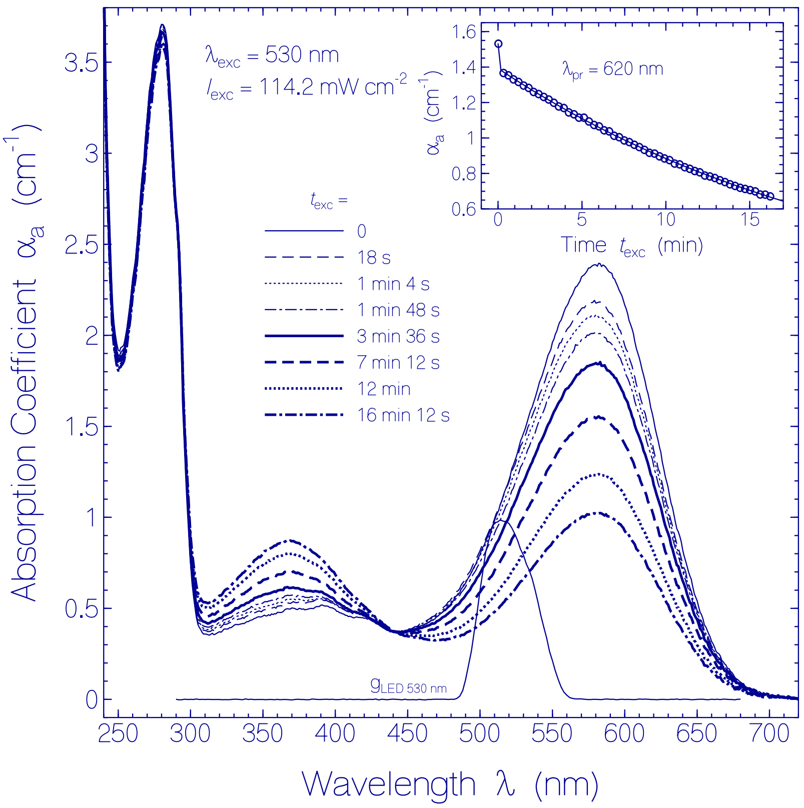

(b)

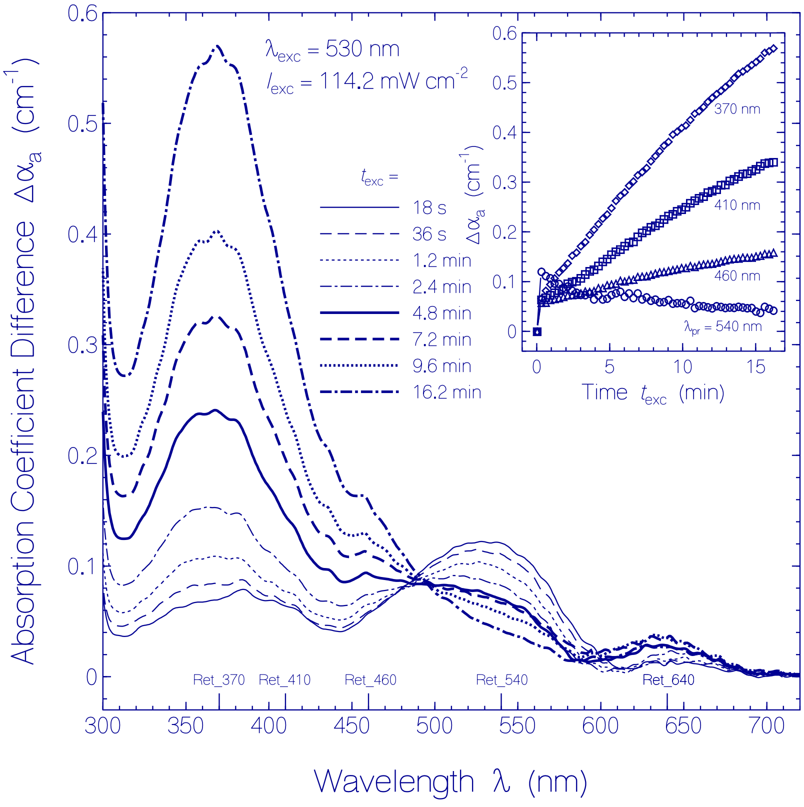

Figure S6. (a) Development of absorption coefficient spectra of a fresh thawed QuasAr1 sample in pH 8 Tris buffer during light exposure with LED 530 nm with input excitation intensity of I_{exc} = 114.2 mW cm^{-2}. The durations of light exposure are listed in the figure. The curve $g_{LED\ 530\ nm}(\lambda) = S_{LED\ 530\ nm}(\lambda)/S_{LED\ 530\ nm}(\lambda_{max})$ shows the spectral distribution of the excitation light source LED 530 nm. The inset shows the temporal dependence of α_a(620 nm) versus exposure time t_{exc}. The data points are fitted by $\alpha_a(t_{exc}) = \alpha_a(0) - \Delta\alpha_I\left[1-\exp\left(-t_{exc}/\tau_{sat,I}\right)\right] - \Delta\alpha_{II}\left[1-\exp\left(-t_{exc}/\tau_{sat,II}\right)\right]$ with $\alpha_a(0)$ =

1.53 cm^{-1}, $\Delta\alpha_I$ = 0.142 cm^{-1}, $\tau_{sat,I}$ = 0.02 min, $\Delta\alpha_{II}$ = 1.392 cm^{-1}, and $\tau_{sat,II}$ = 22.04 min.
(b) Absorption coefficient spectra of formed species of QuasAr1 in pH 8 Tris buffer due to light exposure with LED 530 nm of input intensity I_{exc} = 114.2 mW cm^{-2}. The absorption contribution of Ret_580, $\alpha_{a,Ret\_580}(\lambda,t_{exc})$, and of the initial residuals, $\alpha_{a,Residuals}(\lambda,0)$, are subtracted, i.e., $\Delta\alpha_a(\lambda,t_{exc}) = \alpha_a(\lambda,t_{exc}) - \alpha_{a,\mathrm{Ret\_580}}(\lambda,t_{exc}) - \alpha_{a,\mathrm{Residuals}}(\lambda,t_{exc}=0)$. The inset shows the temporal development of $\Delta\alpha_a$ at λ_{pr} = 540 nm, 460 nm, 410 nm, and 370 nm versus exposure time t_{exc}.

In Figure S6b, the temporal development $\Delta\alpha_a(\lambda,t_{exc}) = \alpha_a(\lambda,t_{exc}) - \alpha_{a,\mathrm{Ret\_580}}(\lambda,t_{exc}) - \alpha_{a,\mathrm{Residuals}}(\lambda,t_{exc}=0)$ of the formed species during light exposure is displayed. New absorption bands are seen around λ ≈ 640 nm (PRSB Ret_640), ≈ 540 nm (PRSB Ret_540), ≈ 460 nm (PRSB Ret_460), ≈ 410 nm (RSB Ret_410), and ≈ 370 nm (RSB Ret_370). The temporal developments of $\Delta\alpha_a$ at the probe wavelengths λ_{pr} = 540 nm, 460 nm, 410 nm, and 370 nm are depicted in the inset of Figure S6b. Within the first 18 s $\Delta\alpha_a$ increased for all wavelengths, then $\Delta\alpha_a$(540 nm) decreased with exposure time, while $\Delta\alpha_a$ at 460 nm, 410 nm and 370 nm continued to increase.

The attenuation coefficient spectra development of the QuasAr1 sample used in Figure S6a after excitation light switch-off over a recovery time range of slightly more than two days (sample in the dark at room temperature) is displayed in Figure S7. The inset in Figure S7 shows the temporal attenuation coefficient development at λ_{pr} = 580 nm and 370 nm. The corresponding absorption coefficient spectra development (scattering contributions approximately subtracted) is shown in Figure S8. The absorption band centered at 580 nm (Ret_580) recovered partly, and the formed absorption band formed around 370 nm (Ret_370 including Ret_410) disappeared partly. The absorption band around 280 nm (dominant tryptophan absorption) increased steadily due to thermal apoprotein restructuring [33]. The inset in Figure S8 shows the partial absorption coefficient recovery at λ_{pr} = 580 nm were the absorption is determined by Ret_580, and the partial absorption coefficient decrease at λ_{pr} = 370 nm due to reprotonation of Ret_370 and Ret_410 to Ret_580. The only partial reconversion is caused by the dynamic thermal apoprotein restructuring [33] during the slow recovery time of the photocycle process.

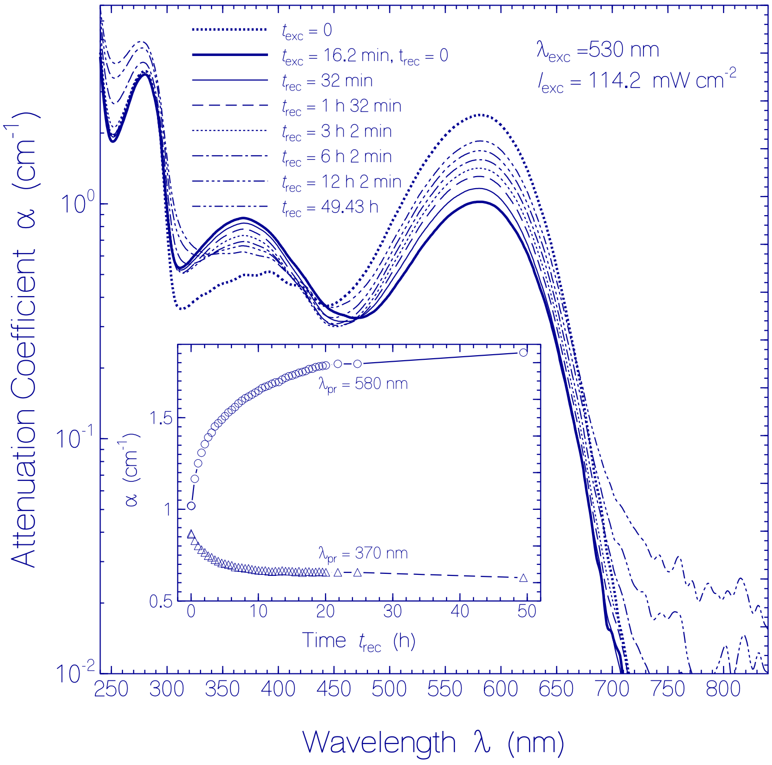

Figure S7. Attenuation coefficient spectra recovery of QuasAr1 in pH 8 Tris buffer after light exposure with LED 530 nm (input excitation intensity I_{exc} = 114.2 mW cm^{-2}) for an exposure time of

t_{exc} = 16.2 min (see Figure S6a). The durations of recovery t_{rec} are listed in the figure. The attenuation coefficient spectra before exposure (t_{exc} = 0) and at end of exposure (t_{exc} = 16.2 min) are included. The inset shows the attenuation coefficient recovery $\alpha(t_{rec})$ at λ_{pr} = 580 nm and 370 nm.

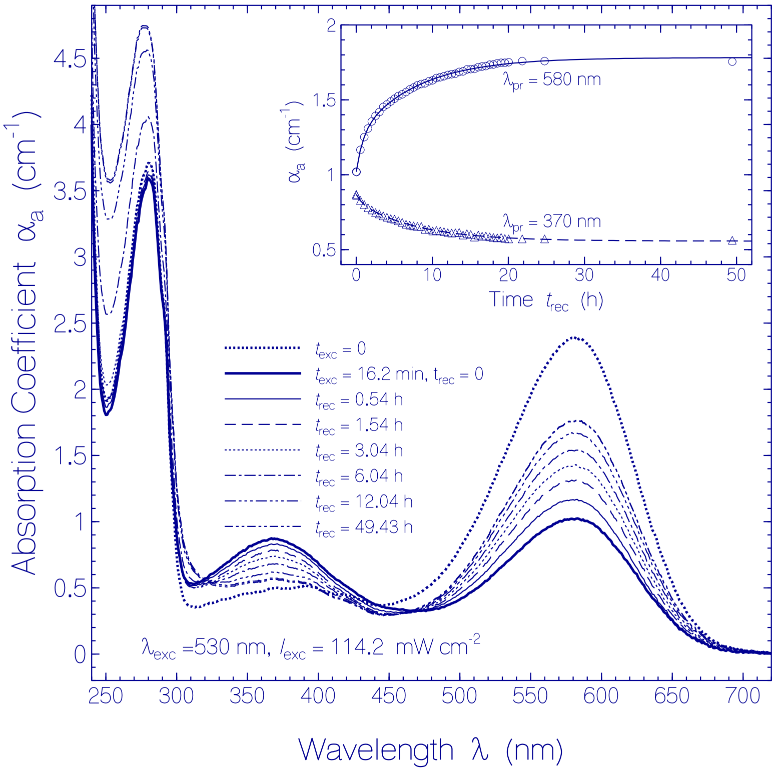

Figure S8. Absorption coefficient spectra recovery of QuasAr1 in pH 8 Tris buffer after light exposure with LED 530 nm (input excitation intensity I_{exc} = 114.2 mW cm^{-2}) for an exposure time of t_{exc} = 16.2 min (see Figure S6a). The durations of recovery t_{rec} are listed in the figure. The absorption coefficient spectra before exposure (t_{exc} = 0) and at end of exposure (t_{exc} = 16.2 min) are included. The inset shows the absorption coefficient recovery $\alpha_a(t_{rec})$ at λ_{pr} = 580 nm and 370 nm. The data points are fitted by $\alpha_a(t_{rec}) = \alpha_a(0) + \Delta\alpha_{a,I}\left[1-\exp\left(-t_{rec}/\tau_{rec,I}\right)\right] + \Delta\alpha_{a,II}\left[1-\exp\left(-t_{rec}/\tau_{rec,II}\right)\right]$ with α_a(0,580 nm) = 1.02 cm^{-1}, $\Delta\alpha_{a,I}$(580 nm) = 0.238 cm^{-1}, $\tau_{rec,I}$(580 nm) = 0.87 h, $\Delta\alpha_{a,II}$(580 nm) = 0.525 cm^{-1}, $\tau_{rec,II}$(580 nm) = 7.81 h, α_a(0,370 nm) = 0.869 cm^{-1}, $\Delta\alpha_{a,I}$(370 nm) = -0.0462 cm^{-1}, $\tau_{rec,I}$(370 nm) = 0.722 h, $\Delta\alpha_{a,II}$(370 nm) = -0.265 cm^{-1}, and $\tau_{rec,II}$(370 nm) = 7.9 h.

The temporal absorption coefficient developments at λ_{pr} = 580 nm, 530 nm, and 367 nm are displayed in Figure S9 for a fresh thawed QuasAr1 sample in pH 8 Tris buffer. In the first run, the probe wavelength was set to λ_{pr} = 580 nm, the exposure time was t_{exc} = 1.5 s, and the time interval of recovery in the dark was set to 10 min. Then, it followed immediately the second run with the same exposure/dark parameters at λ_{pr} = 530 nm. After that it followed immediately the third run with the same exposure/dark parameters at λ_{pr} = 367 nm.

The top part of Figure S9 shows the absorption development at λ_{pr} = 580 nm. During light exposure, the absorption decreased dominantly by photoisomerization of Ret_580$_I$ to Ret_540. After excitation light switch-off, initially a minute absorption decrease was observed likely due to the conversion of Ret_540 to Ret_410 (absorption band of Ret_540 extends out to 580 nm). The following slight absorption increase is thought to be due to partial reprotonation and back isomerization of Ret_410 to Ret_580$_I$ (see discussion in the main part, section 3).

The middle part of Figure S9 shows the absorption development at λ_{pr} = 530 nm in a second exposure of the sample. The absorption decrease, during light exposure, is due to the absorption decrease of the broad absorption band of Ret_580 which dominates over the absorption increase of

the absorption band of the formed Ret_540. The weaker absorption decrease, as compared with λ_{pr} = 580 nm, is due to the formation of the absorption band of Ret_540 during light exposure. The photoexcitation of Ret_540 caused back photoisomerization of Ret_540 to Ret_580$_{I}$ (see discussion in the main part, Section 3). After light switch-off the absorption at 530 nm decreased because of deprotonation of Ret_540 to Ret_410 (fitted time constant τ_{rel,Ret_540} = 41.6 s, see discussion below). The spike at the position of light switch-on is thought to be an artifact caused by a photoinduced transient thermal grating effect [34,35].

The bottom part of Figure S9 shows the absorption development at λ_{pr} = 367 nm in a third exposure of the sample. After excitation light switch-off ($t_{exc,end}$ = 1.5 s) the absorption continued to increase within the first 40 s and then leveled off (fitted time constant τ_{rel,Ret_640} = 14.4 s). The absorption dynamics is thought to be dominated by conversion of Ret_640 to Ret_370 by protonated retinal Schiff base deprotonation (see discussion in the main part, Section 3). The spike at the position of light switch-off is thought to be an artifact caused by a photoinduced transient thermal grating effect [34,35].

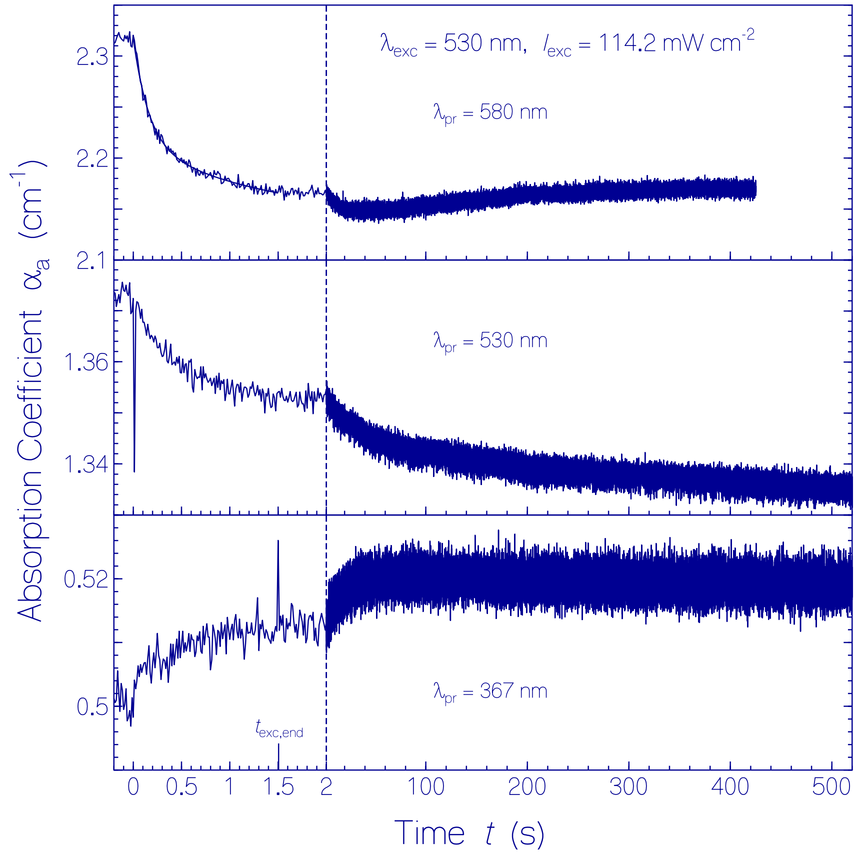

Figure S9. Temporal absorption coefficient development of QuasAr1 in pH 8 Tris buffer at the probe wavelengths λ_{pr} = 580 nm (top part, peak absorption of Ret_580), 530 nm (middle part, near peak absorption of Ret_540), and 367 nm (bottom part, peak absorption of Ret_370) before, during, and after photoexcitation with LED 530 nm of excitation intensity I_{exc} = 114.2 mW cm^{-2} for a duration of t_{exc} = 1.5 s. The same sample was used. Immediately after measurement at λ_{pr} = 580 nm the measurement was continued at λ_{pr} = 530 nm, and then at λ_{pr} = 367 nm. In the top left subfigure the data points, during light exposure, are fitted by $\alpha_a(t_{exc}) = \alpha_a(0) - \Delta\alpha_{a,I}\left[1-\exp\left(-t_{exc}/\tau_I\right)\right] - \Delta\alpha_{a,II}\left[1-\exp\left(-t_{exc}/\tau_{II}\right)\right]$ with $\alpha_a(0)$ = 2.32 cm^{-1}, $\Delta\alpha_{a,I}$ = 0.104 cm^{-1}, τ_I = 160 ms, $\Delta\alpha_{a,II}$ = 0.076 cm^{-1}, and τ_{II} = 1.47 s. A fit of the right part of the middle subfigure with $\alpha_a(t > t_{exc,end}) = \alpha_a(t_{exc,end}) - \Delta\alpha_a\left[1-\exp\left(-\left(t-t_{exc,end}\right)/\tau_{rel,\text{Ret\_540}}\right)\right]$ gives $\alpha_a(t_{exc,end})$ = 1.354 cm^{-1}, $\Delta\alpha_a$ = 0.013 cm^{-1}, and τ_{rel,Ret_540} = 41.6 s. A fit of the right part of the bottom subfigure with $\alpha_a(t > t_{exc,end}) = \alpha_a(t_{exc,end}) + \Delta\alpha_a\left[1-\exp\left(-\left(t-t_{exc,end}\right)/\tau_{rel,\text{Ret\_640}}\right)\right]$ gives $\alpha_a(t_{exc,end})$ = 0.512 cm^{-1}, $\Delta\alpha_a$ = 0.00845 cm^{-1}, and τ_{rel,Ret_640} = 14.4 s.

S1.3. Photoexcitation with Helium Neon Laser at 632.8 nm

At λ_{exc} = 632.8 nm, the absorption of a fresh thawed QuasAr1 sample is caused by the long-wavelength absorption tail of Ret_580. The photoexcitation of Ret_580 causes mainly photoisomerization of Ret_580I to Ret_540 and of Ret_580II to Ret_640. The formed Ret_640 is photoexcited by light exposure at 632.8 nm and causes a partial back photoisomerization of Ret_640 to Ret_580II (see discussion in the main part, Section 3).

(a)

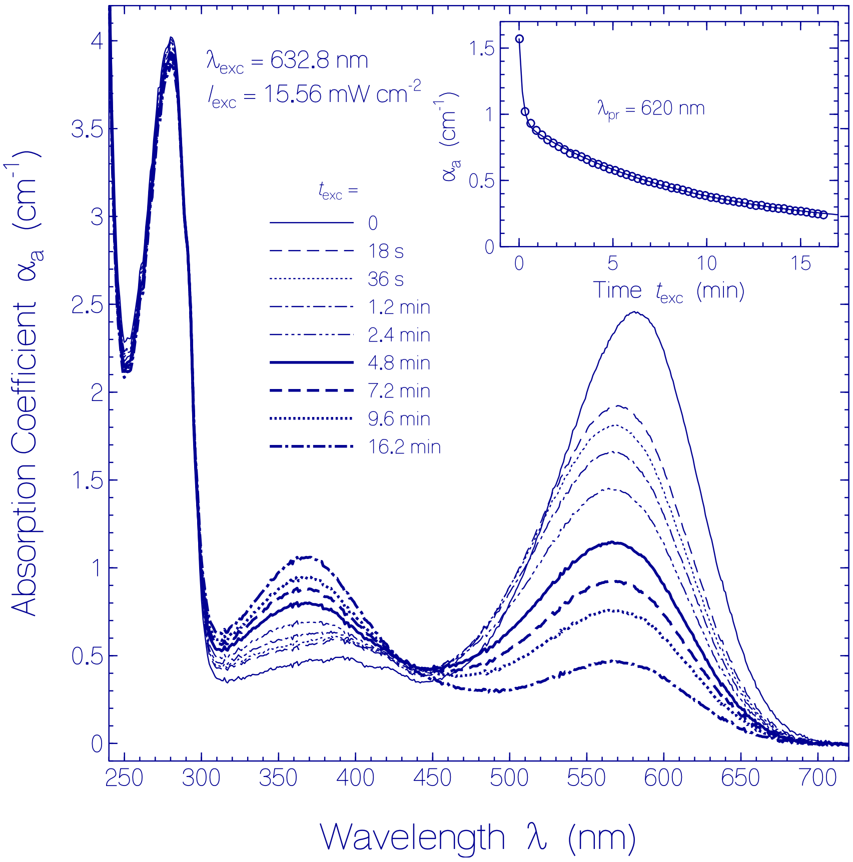

(b)

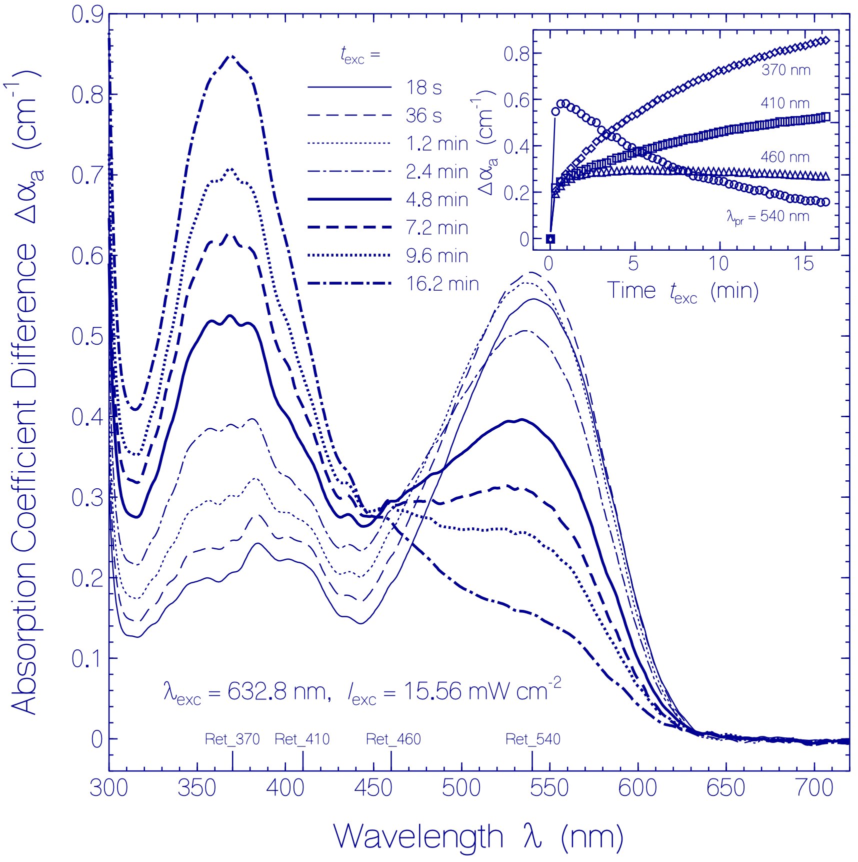

Figure S10. (a) Development of absorption coefficient spectra of a fresh thawed QuasAr1 sample in pH 8 Tris buffer, during light exposure, with helium neon laser (λ_{exc} = 632.8 nm) with input excitation intensity of I_{exc} = 15.56 mW cm^{-2}. The durations of light exposure are listed in the figure. The inset shows the temporal dependence of α_a(620 nm) versus exposure time t_{exc}. The data points are fitted by $\alpha_a(t_{exc}) = \alpha_a(0) - \Delta\alpha_I\left[1-\exp\left(-t_{exc}/\tau_{sat,I}\right)\right] - \Delta\alpha_{II}\left[1-\exp\left(-t_{exc}/\tau_{sat,II}\right)\right]$ with $\alpha_a(0)$ = 1.57 cm^{-1}, $\Delta\alpha_I$ = 0.629 cm^{-1}, $\tau_{sat,I}$ = 0.179 min, $\Delta\alpha_{II}$ = 0.815 cm^{-1}, and $\tau_{sat,II}$ = 8.56 min.

(b) Absorption coefficient spectra of formed species. The absorption contribution of Ret_580, $\alpha_{a,Ret\_580}(\lambda,t_{exc})$, and of the initial residuals, $\alpha_{a,Residuals}(\lambda,0)$, are subtracted, i.e. $\Delta\alpha_a(\lambda,t_{exc}) = \alpha_a(\lambda,t_{exc}) - \alpha_{a,\mathrm{Ret\_580}}(\lambda,t_{exc}) - \alpha_{a,\mathrm{Residuals}}(\lambda,t_{exc}=0)$. The inset shows the temporal development of $\Delta\alpha_a$ at λ_{pr} = 540 nm, 460 nm, 410 nm, and 370 nm versus exposure time t_{exc}.

In Figure S10a, the development of absorption coefficient spectra of a fresh thawed QuasAr1 sample in pH 8 buffer, during light exposure, with a helium neon laser (λ_{exc} = 632.8 nm and input intensity I_{exc} = 15.56 mW cm^{-2}) is displayed. The absorption coefficient curves belong to the exposure times listed in the legend. With increasing exposure time, the curves show the decrease of the absorption band around 580 nm and the dominant buildup of an absorption band around 370 nm. The inset in Figure S10a shows the temporal development of the absorption coefficient $\alpha_a(t_{exc})$ at the probe wavelength λ_{pr} = 620 nm. It indicates an initially fast absorption decrease (photoconversion of Ret_580$_{I}$ component) followed by a slow absorption decrease (photoconversion of Ret_580$_{II}$ component).

In Figure S10b, the temporal development $\Delta\alpha_a(\lambda,t_{exc}) = \alpha_a(\lambda,t_{exc}) - \alpha_{a,\mathrm{Ret\_580}}(\lambda,t_{exc}) - \alpha_{a,\mathrm{Residuals}}(\lambda,t_{exc}=0)$ of the formed species, during light exposure, is displayed. New absorption bands are seen around $\lambda \approx$ 540 nm (PRSB Ret_540), ≈ 460 nm (PRSB Ret_460, small contribution), ≈ 410 nm (RSB Ret_410), and ≈ 370 nm (RSB Ret_370). No new absorption band around $\lambda \approx$ 640 nm (PRSB Ret_640) is seen, because the excitation laser at 632.8 nm photoexcites Ret_640 with photoisomerization back to Ret_580$_{II}$. The temporal developments of $\Delta\alpha_a$ at the probe wavelengths λ_{pr} = 540 nm, 460 nm, 410 nm, and 370 nm are depicted in the inset of

Figure S10b. Within the first minute, $\Delta\alpha_a$ increased for all wavelengths, then $\Delta\alpha_a$(540 nm) decreased with exposure time, $\Delta\alpha_a$(460 nm) remained approximately constant, while $\Delta\alpha_a$ at 410 nm and 370 nm continued to increase.

The attenuation coefficient spectra development of the QuasAr1 sample used in Figure S10a after excitation light switch-off over a recovery time range of slightly more than two days (sample in the dark at room temperature) is displayed in Figure S11. The inset in Figure S11 shows the temporal attenuation coefficient development at λ_{pr} = 580 nm and 370 nm. The corresponding absorption coefficient spectra development (scattering contributions approximately subtracted) is shown in Figure S12. The absorption band centered at 580 nm (Ret_580) recovered partly, and the formed absorption bands formed around 460 nm, 410 nm, and 370 nm decreased slightly. The absorption band around 280 nm (dominant tryptophan absorption) and its absorption tail out to 400 nm (likely due to dityrosine [47], tyrosinyl radicals [48], and tryptophanyl radicals [49]) increased steadily due to thermal apoprotein restructuring [33] (some long-time interaction of the buffer components with the apoprotein cannot be excluded). The inset in Figure S12 shows the partial absorption coefficient recovery at λ_{pr} = 580 nm where the absorption is determined by Ret_580, and the partial absorption coefficient decrease at λ_{pr} = 370 nm due to reprotonation of Ret_370 and Ret_410 to Ret_580. The only partial reconversion is caused by the dynamic thermal apoprotein restructuring [33], during the slow recovery time of the photocycle process.

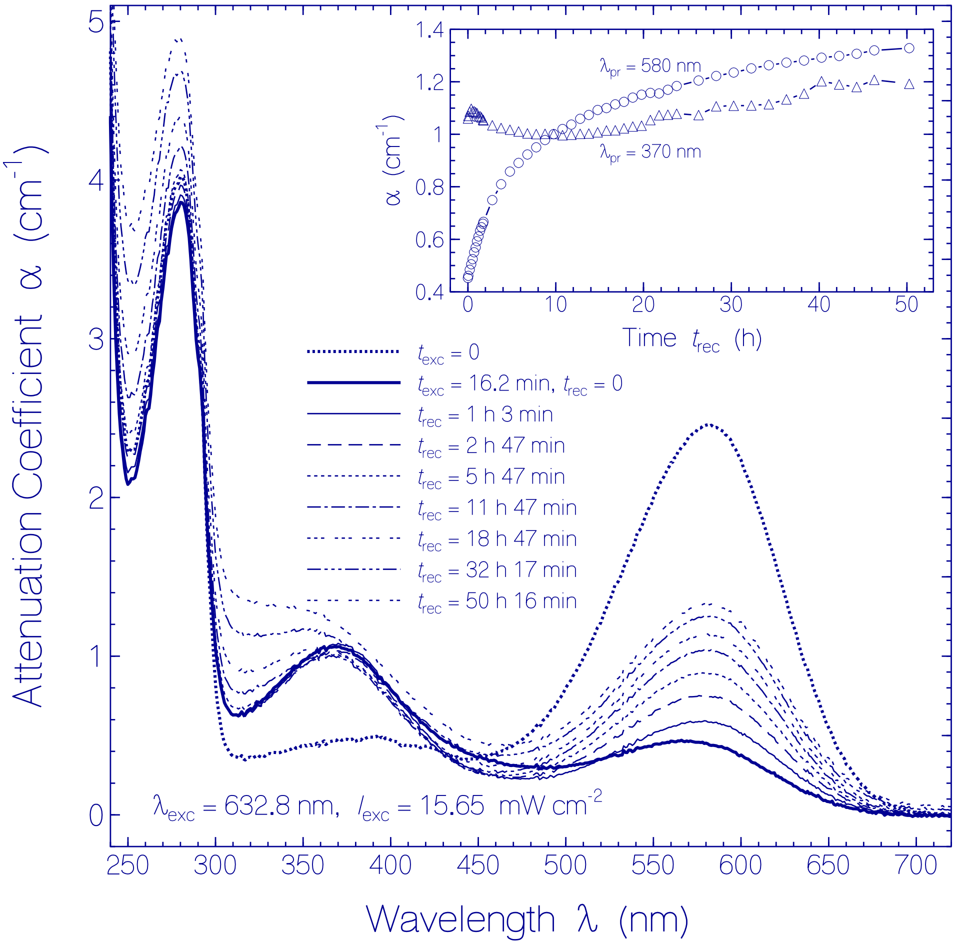

Figure S11. Attenuation coefficient spectra recovery of QuasAr1 in pH 8 Tris buffer after light exposure with a helium neon laser (λ_{exc} = 632.8 nm) of excitation intensity I_{exc} = 15.65 mW cm^{-2} for an exposure time of t_{exc} = 16.2 min (see Figure S10a). The durations of recovery t_{rec} are listed in the figure. The attenuation coefficient spectra before exposure (t_{exc} = 0) and at end of exposure (t_{exc} = 16.2 min) are included. The inset shows the attenuation coefficient recovery $\alpha(t_{rec})$ at λ_{pr} = 580 nm and 370 nm.

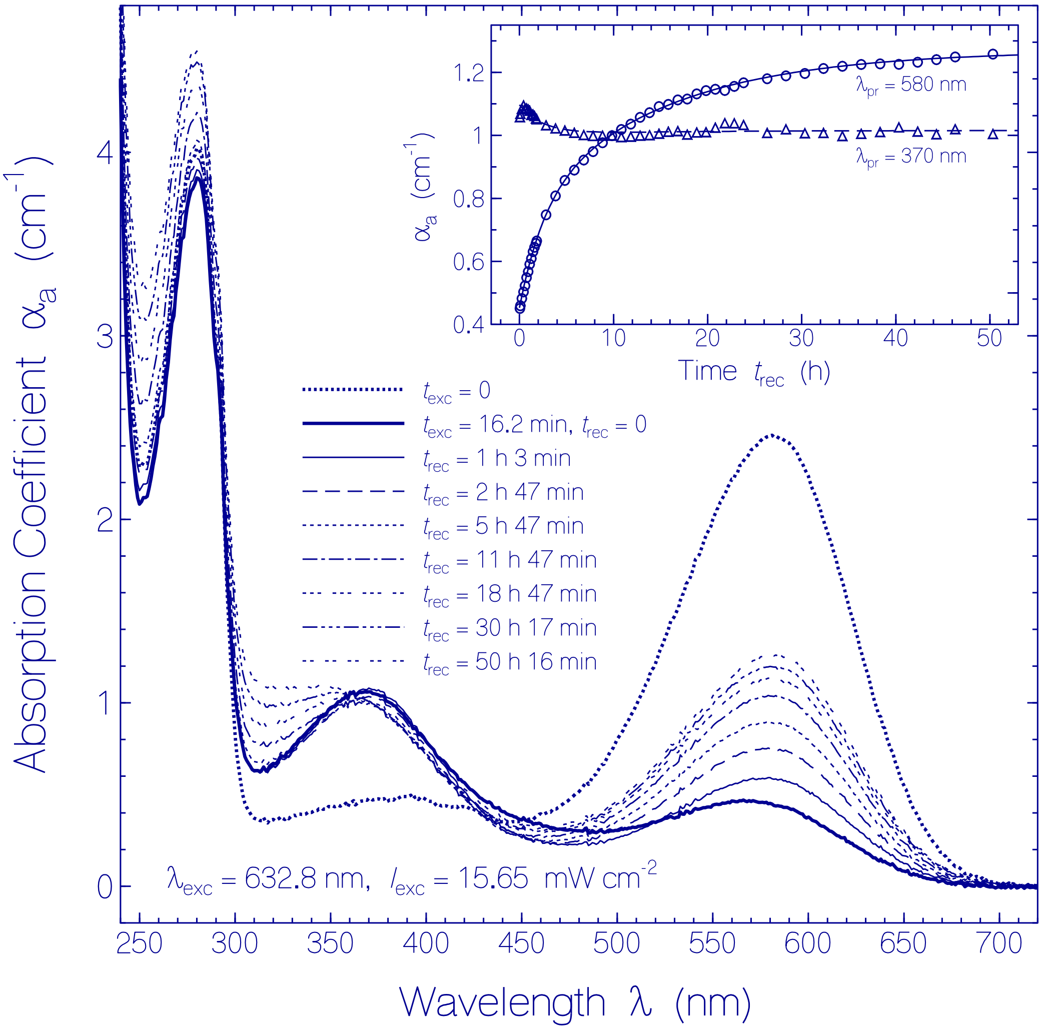

Figure S12. Absorption coefficient spectra recovery of QuasAr1 in pH 8 Tris buffer after light exposure with helium neon laser (λ_{exc} = 632.8 nm) of excitation intensity I_{exc} = 15.65 mW cm^{-2} for an exposure time of t_{exc} = 16.2 min (see Figure S10a). The durations of recovery t_{rec} are listed in the figure. The absorption coefficient spectra before exposure (t_{exc} = 0) and at end of exposure (t_{exc} = 16.2 min) are included. The inset shows the absorption coefficient recovery $\alpha_a(t_{rec})$ at λ_{pr} = 580 nm and 370 nm. The data points for λ_{pr} = 580 nm and 370 nm are fitted by $\alpha_a(t_{rec}) = \alpha_a(0) + \Delta\alpha_{a,I}\left[1-\exp\left(-t_{rec}/\tau_{rec,I}\right)\right] + \Delta\alpha_{a,II}\left[1-\exp\left(-t_{rec}/\tau_{rec,II}\right)\right]$ with α_a(0,580 nm) = 0.452 cm^{-1}, $\Delta\alpha_{a,I}$(580 nm) = 0.312 cm^{-1}, $\tau_{rec,I}$(580 nm) = 2.56 h, $\Delta\alpha_{a,II}$(580 nm) = 0.505 cm^{-1}, $\tau_{rec,II}$(580 nm) = 14.7 h, α_a(0,370 nm) = 1.1 cm^{-1}, $\Delta\alpha_{a,I}$(370 nm) = -0.097 cm^{-1}, $\tau_{rec,I}$(370 nm) = 2.5 h, $\Delta\alpha_{a,II}$(370 nm) = 0.0156 cm^{-1}, and $\tau_{rec,II}$(370 nm) = 12.8 h.

The temporal absorption coefficient developments at λ_{pr} = 580 nm, 530 nm, and 367 nm are displayed in Figure S13 for a fresh thawed QuasAr1 sample in pH 8 Tris buffer. In the first run, the probe wavelength was set to λ_{pr} = 580 nm, the exposure time was t_{exc} = 58 s, and the time of recovery in the dark was 10 min. Then, it followed immediately the second run with an exposure time of 18 s and a recovery time in the dark of 10 min at λ_{pr} = 530 nm. After that it followed immediately the third run with 18 s exposure and 10 min recovery at λ_{pr} = 367 nm.

The top part of Figure S13 shows the absorption development at λ_{pr} = 580 nm. During light exposure, the absorption decreased by photoisomerization of Ret_580$_I$ to Ret_540, photoisomerization of Ret_580$_{II}$ to Ret_640 and some deprotonation of Ret_540 to Ret_410 and of Ret_640 to Ret_370. After excitation light switch-off a slight absorption increase was observed likely due to reprotonation of Ret_410 to Ret_580$_I$ (see discussion in the main part, Section 3).

The middle part of Figure S13 shows the absorption development at λ_{pr} = 530 nm in a second exposure of the sample. The absorption decrease, during light exposure, is due to the absorption decrease of the broad absorption band of Ret_580 which dominates the absorption at 530 nm. The weaker absorption decrease, as compared with λ_{pr} = 580 nm, is due to the formation of the absorption band of Ret_540 during light exposure. After light switch-off ($t_{exc,end}$ = 18 s) the absorption at 530 nm decreased because of deprotonation of Ret_540 to Ret_410 (see discussion in the main part, Section 3).

The bottom part of Figure S13 shows the absorption development at λ_{pr} = 367 nm in a third exposure of the sample. During light exposure, the absorption increased (conversion of Ret_580$_I$ to Ret_410 and of Ret_580$_{II}$ to Ret_370). After excitation light switch-off ($t_{exc,end}$ = 18 s) the absorption continued to increase within the first 20 s dominantly due to the deprotonation of Ret_640 to Ret_370.

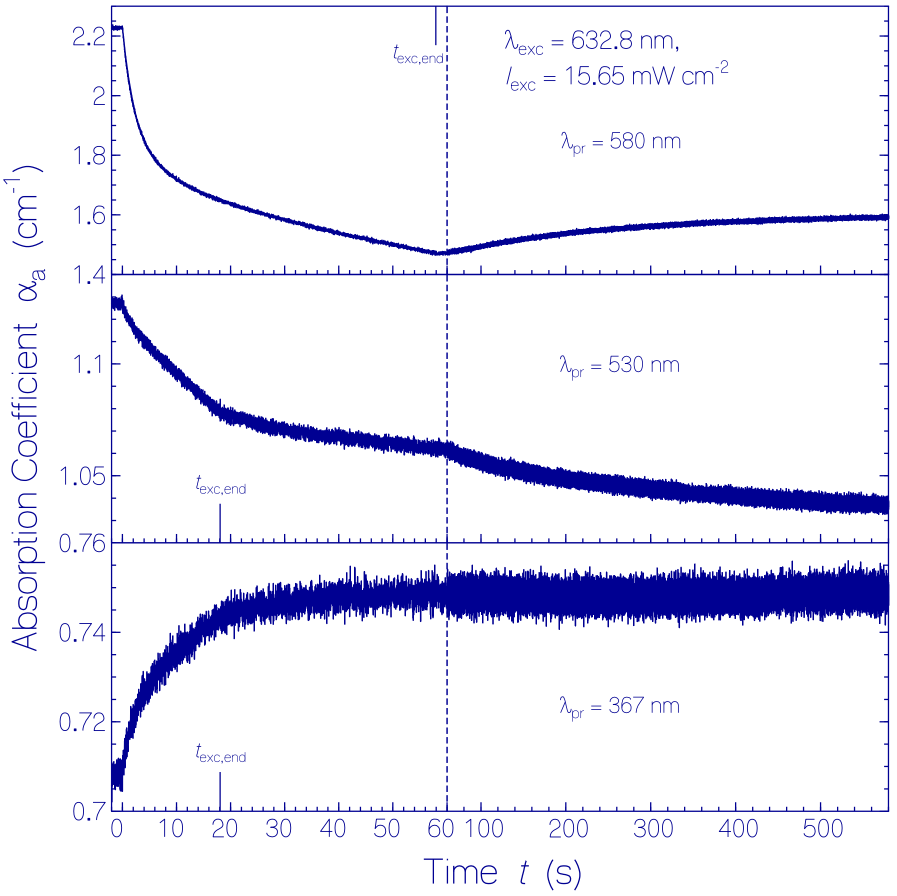

Figure S13. Temporal absorption coefficient development of QuasAr1 in pH 8 Tris buffer at the probe wavelengths λ_{pr} = 580 nm (top part, peak absorption of Ret_580, duration of exposure t_{exc} = 58 s), 530 nm (middle part, near peak absorption of Ret_540, t_{exc} = 18 s), and 367 nm (bottom part, peak absorption of Ret_370, t_{exc} = 18 s) before, during, and after photoexcitation with helium neon laser of excitation intensity I_{exc} = 15.65 mW cm^{-2}. The same sample was used. Immediately after measurement at λ_{pr} = 580 nm the measurement was continued at λ_{pr} = 530 nm, and then at λ_{pr} = 367 nm. In the top left subfigure the data points, during light exposure, are fitted by $\alpha_a(t_{exc}) = \alpha_a(0) - \Delta\alpha_{a,I}\left[1-\exp\left(-t_{exc}/\tau_I\right)\right] - \Delta\alpha_{a,II}\left[1-\exp\left(-t_{exc}/\tau_{II}\right)\right]$ with $\alpha_a(0)$ = 2.23 cm^{-1}, $\Delta\alpha_{a,I}$ = 0.431 cm^{-1}, τ_I = 2.51 s, $\Delta\alpha_{a,II}$ = 0.454 cm^{-1}, and τ_{II} = 46.7 s.

S2. Fluorescence Emission Spectra of QuasAr1 in Tris pH 8 Buffer after Light Exposure

In Figure S14, total fluorescence emission quantum distributions, $E_F(\lambda_F)$, are shown for the QuasAr1 sample in pH 8 Tris buffer used for the photocylce studies of Figure 1a (sample excitation

with LED 590 nm of input intensity I_{exc} = 64.65 mW cm^{-2} for a duration of t_{exc} = 25 min). $E_F(\lambda_F)$ is defined by [50]

$$E_F(\lambda_F) = \frac{S_F(\lambda_F)}{S_{abs}} = \frac{\sum_i S_{F,i}(\lambda_F)}{S_{abs}} \quad \text{(S1)}$$

where $S_F(\lambda_F)$ is the total intrinsic spectral fluorescence photon number density distribution, S_{abs} is the total number of absorbed excitation photons, and $S_{F,i}(\lambda_F)$ is the intrinsic spectral fluorescence photon number density distribution of species *i*.

In Figure S14a, the fluorescence spectra were recorded immediately after the end of the photoexcitation, and in Figure S14b the fluorescence spectra were recorded five days after the end of the photoexcitation. The various $E_F(\lambda_F)$ curves belong to different fluorescence excitation wavelengths $\lambda_{F,exc}$ in the range from $\lambda_{F,exc}$ = 640 nm to 260 nm. For $\lambda_{F,exc}$ > 550 nm, only Ret_580 and possibly minor present Ret_640 contribute to $E_F(\lambda_F)$. For shorter fluorescence excitation wavelengths, originally present and formed retinal isomers are excited and contribute to the $E_F(\lambda_F)$ curves. For $\lambda_{F,exc}$ < 310 nm, the fluorescence emission in the wavelength range λ_F < 400 nm is dominated by apoprotein Trp emission. A comparison of Figure S14a with Figure S14b indicates that the fluorescence spectra change with storage time in the dark after the light exposure (relaxations after photoisomerization, thermal isomerizations and protonation changes, and thermal apoprotein restructuring).

(a)

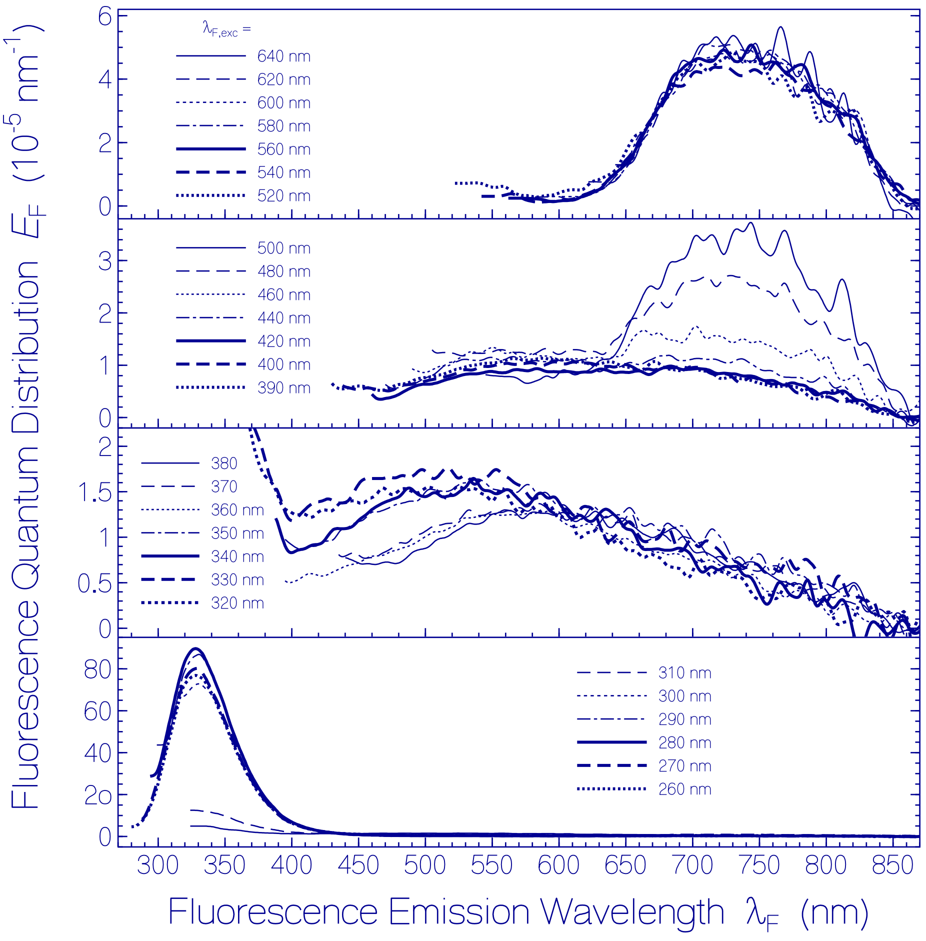

(b)

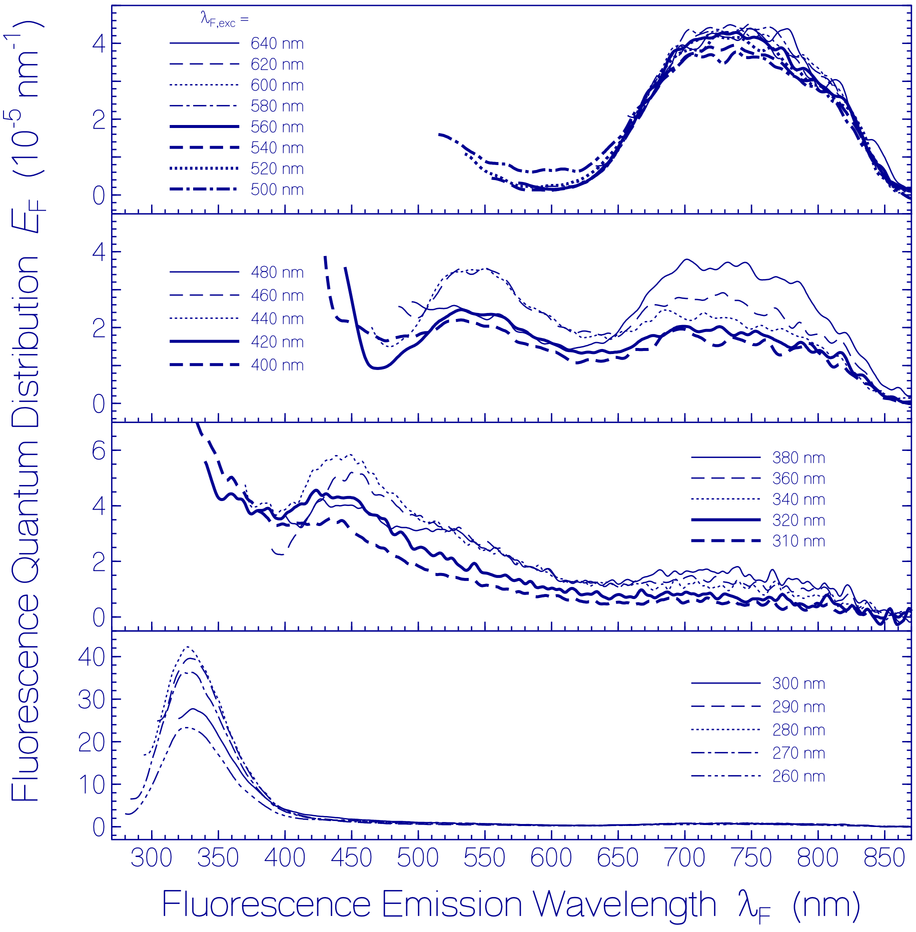

Figure S14. Fluorescence emission quantum distribution $E_F(\lambda_F)$ of QuasAr1 in pH 8 Tris buffer (**a**) immediately after photoexcitation, and (**b**) after 5 days of recovery in the dark at room temperature. The fluorescence excitation wavelengths $\lambda_{F,exc}$ are indicated in the subfigures. Sample excitation was according to Figure 1a.

In Figure S15 the dependence of the total fluorescence quantum yield

$$\phi_F(\lambda_F) = \int_{em} E_F(\lambda_F) d\lambda_F \quad \text{(S2)}$$

on the fluorescence excitation wavelength $\lambda_{F,exc}$ is shown for a fresh thawed QuasAr1 sample (data taken from [33]) and for the photoexcited QuasAr1 sample of Figure 1a (λ_{exc} = 590 nm, I_{exc} = 64.65 mW cm^{-2}, t_{exc} = 25 min) immediately after excitation, and five days after excitation. The fluorescence quantum yield of Ret_580 ($\lambda_{F,exc}$ > 500 nm) is approximately the same before photoexcitation, after photoexcitation, and after thermal recovery in the dark. The retinal isomers that are formed by photoexcitation, immediately after formation, are less fluorescent than Ret_580. This behavior changes with thermal relaxation in the dark where ϕ_F in the fluorescence excitation wavelength region from 310 nm to 500 nm becomes stronger than for the fresh thawed sample. Immediately after photoexcitation, the apoprotein Trp fluorescence quantum yield is larger than in the case of the fresh thawed sample or the thermally relaxed sample. In this case the Trp fluorescence quenching by Förster-type energy transfer [51,52] to retinal isomers is reduced.

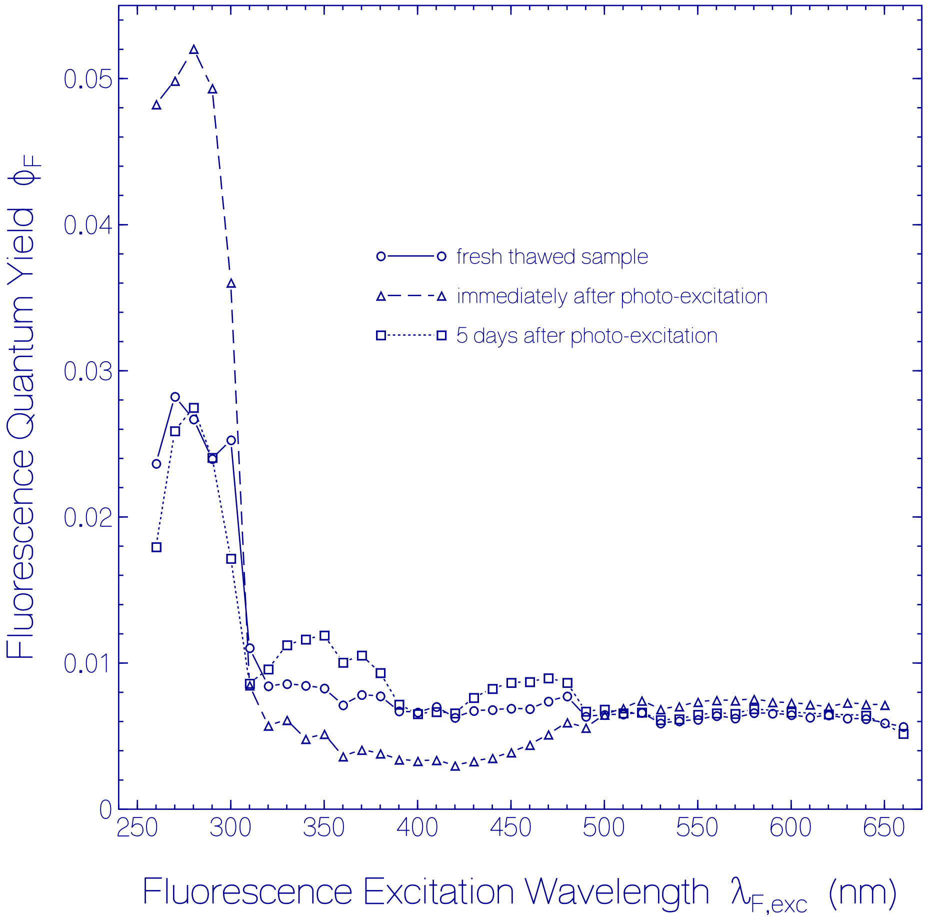

Figure S15. Dependence of fluorescence quantum yield ϕ_F of QuasAr1 in pH 8 Tris buffer on fluorescence excitation wavelength $\lambda_{F,exc}$. Circles, fresh unexposed sample; triangles, fluorescence measured immediately after light exposure at 590 nm with I_{exc} = 64.65 mW cm^{-2} for duration of 25 min; and squares, fluorescence measure of the same exposed sample five days after light switch-off (storage in the dark at room temperature).

S3. Fluorescence Excitation Spectra of QuasAr1 in Tris pH 8 Buffer after Light Exposure and Dark Recovery

In Figure S16, normalized fluorescence excitation quantum distributions $E'_{ex}(\lambda_{exc})$ [33,53] are shown of the photoexcited QuasAr1 sample of Figure 1a (λ_{exc} = 590 nm, I_{exc} = 64.65 mW cm^{-2}, and t_{exc} = 25 min) after five days of recovery in the dark. The corresponding absorption coefficient spectrum $\alpha_a(\lambda)$ of the recovered sample is included. The fluorescence excitation quantum distributions $E_{ex}(\lambda_{exc})$ are normalized according to

$$E'_{ex}(\lambda_{exc}) = E'_{ex,\lambda_{F,det}}(\lambda_{exc}) = \frac{E_{ex,\lambda_{F,det}}(\lambda_{exc})}{E_{ex,\lambda_{F,det}=720\ \mathrm{nm}}(\lambda_{exc}=580\ \mathrm{nm})}\alpha_a(580\ \mathrm{nm}) \quad (S3)$$

In the top subfigure, the $E'_{ex}(\lambda_{exc})$ curves for $\lambda_{F,exc}$ = 780 nm, 740 nm, and 700 nm resemble the absorption coefficient spectrum of Ret_580 in the wavelength range $\lambda_{exc} \geq 310$ nm. For $\lambda_{exc} < 310$ nm, the curves are dominated by apoprotein Trp and Tyr absorption and subsequent Förster-type energy transfer [51,52] to Ret_580. The curves belonging to $\lambda_{F,det}$ = 660 nm and 640 nm include already emissions from shorter wavelength absorbing retinal isomers.

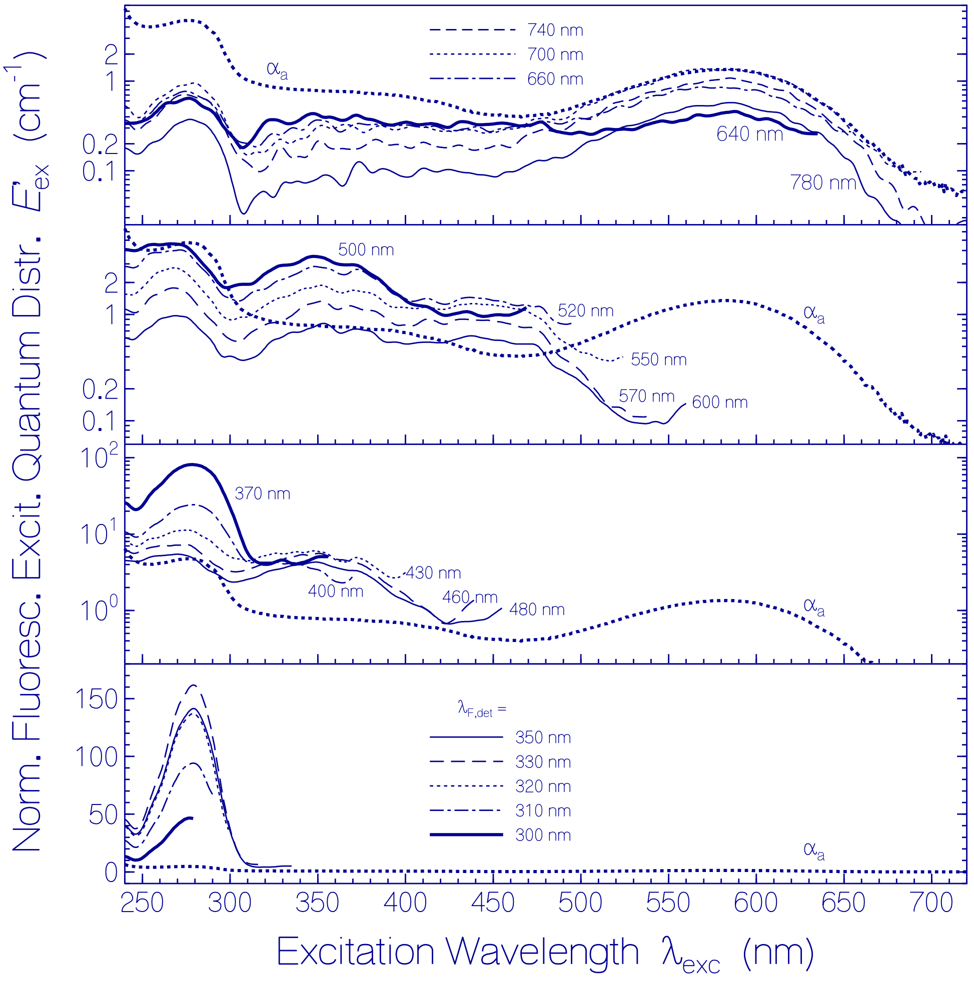

Figure S16. Normalized fluorescence excitation quantum distributions $E'_{ex}(\lambda)$ and corresponding absorption coefficient spectrum $\alpha_a(\lambda_{exc})$ of QuasAr1 in pH 8 Tris buffer after photoexcitation and recovery of five days in the dark at room temperature. The fluorescence detection wavelengths $\lambda_{F,det}$ are indicated in the subfigures. Sample excitation was according to Figure 1a.

In the second-top subfigure, $E'_{ex}(\lambda_{exc})$ curves are displayed for $\lambda_{F,det}$ = 600 nm, 570 nm, 550 nm, 520 nm, and 500 nm. They indicate emission from protonated retinal Schiff base isomers around 450 nm (Ret_450 of thermal relaxed QuasAr1, see [33]) and of deprotonated retinal Schiff base isomers around 350 nm (Ret_350 of thermal relaxed QuasAr1, see [33]). For λ_{exc} < 310 nm, the curves are dominated by apoprotein Trp and Tyr absorption and subsequent Förster-type energy transfer to Ret_350 and Ret_450.

In the second-bottom subfigure, $E'_{ex}(\lambda_{exc})$ curves are displayed for $\lambda_{F,det}$ = 480 nm, 460 nm, 430 nm, 400 nm, and 370 nm. They indicate emission from deprotonated retinal Schiff base isomers around 350 nm (Ret_350 of thermal relaxed QuasAr1, see [33]). For λ_{exc} < 310 nm, the curves again are dominated by apoprotein Trp and Tyr absorption and subsequent Förster-type energy transfer to Ret_350. For λ_{exc} < 310 nm and $\lambda_{F,det}$ = 370 nm, the direct Trp emission dominates.

In the bottom subfigure, the $E'_{ex}(\lambda_{exc})$ curves are determined by emission from Trp and Förster-type energy transfer from Tyr to Trp.

S4. Photocycle Dynamics Calculations

The photocycle dynamics of Ret_580$_{I}$ and of Ret_580$_{II}$ occurs with different photoconversion efficiency ($\phi_{con,\mathrm{Ret\_580_I}}(t_{exc}=0,\lambda_{exc}=590\ nm)$ ≈ 0.056, $\phi_{con,\mathrm{Ret\_580_{II}}}(\lambda_{exc}=590\ nm, I_{exc}\rightarrow 0)$ ≈ 0.00135, Figure

5) and different recovery times ($\tau_{rec,\mathrm{Ret\_410}\rightarrow\mathrm{Ret\_580_I}}$ = $\tau_{rec,I} \approx 1.5$ h, $\tau_{rec,\mathrm{Ret\_370}\rightarrow\mathrm{Ret\_580_{II}}}$ = $\tau_{rec,II} \approx 20$ h, Figure 3a). This different dynamic behavior allows a separation of the Ret_580I and Ret_580II photocycle analysis.

Before light exposure the absorption coefficient spectrum $\alpha_{a,\mathrm{Ret\_580}}(t_{exc} = 0)$ of Ret_580 is composed of contributions from Ret_580I and Ret_580II according to

$$\alpha_{a,\mathrm{Ret\_580}}(0) = \alpha_{a,\mathrm{Ret\_580_I}}(0) + \alpha_{a,\mathrm{Ret\_580_{II}}}(0) \quad \text{(S4)}$$

with

$$\alpha_{a,\mathrm{Ret\_580_I}}(0) = \kappa_{\mathrm{Ret\_580_I}}\alpha_{a,\mathrm{Ret\_580}}(0) \quad \text{(S5)}$$

$$\alpha_{a,\mathrm{Ret\_580_{II}}}(0) = \kappa_{\mathrm{Ret\_580_{II}}}\alpha_{a,\mathrm{Ret\_580}}(0) \quad \text{(S6)}$$

where [33] $\kappa_{\mathrm{Ret\_580_I}} \approx 0.41$ is the fraction of Ret_580I in Ret_580, and $\kappa_{\mathrm{Ret\_580_{II}}} \approx 0.59$ is the fraction of Ret_580II in Ret_580.

For $t_{exc} << \tau_{rec,\mathrm{Ret\_410}\rightarrow\mathrm{Ret\_580_I}} < \tau_{rec,\mathrm{Ret\_370}\rightarrow\mathrm{Ret\_580_{II}}}$ (slow saturable absorption [54]) the excitation intensity dependent temporal development of $\alpha_{a,\mathrm{Ret\_580_I}}(t_{exc})$ and $\alpha_{a,\mathrm{Ret\_580_{II}}}(t_{exc})$ is given by [55]

$$\alpha_{a,\mathrm{Ret\_580_I}}(t_{exc}) = \alpha_{a,\mathrm{Ret\_580_I}}(0)\exp\left(-\frac{t_{exc}}{\tau_{\mathrm{Ret\_580_I},sat}}\right) \quad \text{(S7)}$$

$$\alpha_{a,\mathrm{Ret\_580_{II}}}(t_{exc}) = \alpha_{a,\mathrm{Ret\_580_{II}}}(0)\exp\left(-\frac{t_{exc}}{\tau_{\mathrm{Ret\_580_{II}},sat}}\right) \quad \text{(S8)}$$

where

$$\tau_{\mathrm{Ret\_580_I},sat} = \frac{w_{\mathrm{Ret\_580_I},sat}}{I_{exc}} \quad \text{(S9)}$$

$$w_{\mathrm{Ret\_580_I},sat} = \frac{h\nu_{exc}}{\sigma_{a,\mathrm{Ret\_580}}(\lambda_{exc})\phi_{con,\mathrm{Ret\_580_I}}(I_{exc})} \quad \text{(S10)}$$

$$\tau_{\mathrm{Ret\_580_{II}},sat} = \frac{w_{\mathrm{Ret\_580_{II}},sat}}{I_{exc}} \quad \text{(S11)}$$

$$w_{\mathrm{Ret\_580_{II}},sat} = \frac{h\nu_{exc}}{\sigma_{a,\mathrm{Ret\_580}}(\lambda_{exc})\phi_{con,\mathrm{Ret\_580_{II}}}(I_{exc})} \quad \text{(S12)}$$

$\tau_{\mathrm{Ret\_580_I},sat}$ and $\tau_{\mathrm{Ret\_580_{II}},sat}$ are the saturation time constants. $w_{\mathrm{Ret\_580_I},sat}$ and $w_{\mathrm{Ret\_580_{II}},sat}$ are the saturation energy densities.

For $\lambda_{exc} = 590$ nm and $I_{exc} = 64.65$ mW cm^{-2}, the saturation energy density and the saturation time constant of Ret_580II are $w_{\mathrm{Ret\_580_{II}},sat} \approx 48$ J cm^{-2} ($\sigma_{a,\mathrm{Ret\_580}}(590\ \mathrm{nm}) \approx 1.55\times10^{-16}$ cm^2 [33], $\phi_{con,\mathrm{Ret\_580_{II}}}(I_{exc})$

= 4.53 × 10^-5^, Figure 5) and $\tau_{\mathrm{Ret\_580_{II}},sat}$ = $w_{\mathrm{Ret\_580_{II}},sat}$ /I_{exc} ≈ 740 s. For λ_{exc} = 590 nm, I_{exc} = 64.65 mW cm^{-2}, and t_{exc} → 0 the saturation energy density and the saturation time constant of Ret_580$_{I}$ are $w_{\mathrm{Ret\_580_{I}},sat}$ ≈ 0.0388 J cm^{-2} ($\sigma_{a,\mathrm{Ret\_580}}$(590 nm) ≈ 1.55 × 10^{-16} cm^2 [33], $\phi_{con,\mathrm{Ret\_580_{I}}}(I_{exc}, t_{exc} \to 0)$ ≈ 0.056, Figure 5) and $\tau_{\mathrm{Ret\_580_{I}},sat}$ = $w_{\mathrm{Ret\_580_{I}},sat}$ /I_{exc} ≈ 0.6 s.

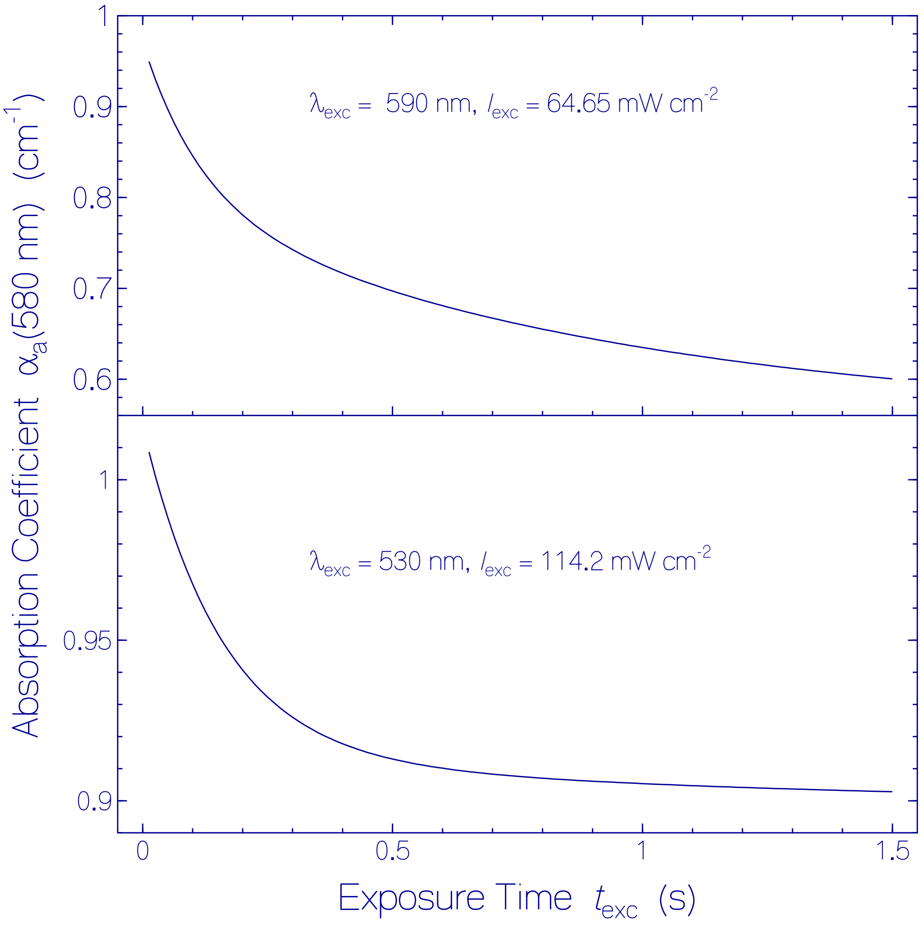

Figure S17. Temporal absorption coefficient development of Ret_580$_{I}$ at λ_{pr} = 580 nm during photoexcitation: (**Top part**) λ_{exc} = 590 nm, I_{exc} = 64.65 mW cm^{-1} and (**Bottom part**) λ_{exc} = 530 nm, I_{exc} = 114.2 mW cm^{-1}.

For $t_{exc} \ll \tau_{\mathrm{Ret\_580_{II}},sat}$ the absorption coefficient development of Ret_580$_{I}$ is approximately given by

$$\alpha_{a,\mathrm{Ret\_580_{I}}}(t_{exc}) = \alpha_{a,\mathrm{Ret\_580}}(t_{exc}) - \alpha_{a,\mathrm{Ret\_580_{II}}}(0) \quad \text{(S13)}$$

The temporal development of $\alpha_{a,\mathrm{Ret\_580_{I}}}(t_{exc}, \lambda_{pr} = 580\ nm)$ is shown in Figure S17 for λ_{exc} = 590 nm, I_{exc} = 64.65 mW cm^{-2} in the top part, and for λ_{exc} = 530 nm, I_{exc} = 114.2 mW cm^{-2} in the bottom part.

S4.1. Photocycle Dynamics Calculations for Ret_580$_{I}$

Schemes of the photocycle dynamics of Ret_580$_{I}$ are shown in Figures 6a and 7a. The absorption coefficient development at λ_{pr} = 530 nm of the middle parts of Figures 4 and S9, after excitation light switch-off, reveals a Ret_540 relaxation time constant of $\tau_{rel,\mathrm{Ret\_540}}$ ≈ 39 s.

For $t_{exc} \ll \tau_{rel,Ret\_540}$ and $t_{exc} \ll \tau_{Ret\_580_{II},sat}$, the photoexcitation dynamics of Ret_580I and Ret_540 are approximately given by

$$\frac{dN_{Ret\_580_I}}{dt_{exc}} = \frac{I_{exc}}{h\nu_{exc}}\left(-N_{Ret\_580_I}\sigma_{a,Ret\_580}(\lambda_{exc})\phi_{iso,Ret\_580_I} + N_{Ret\_540}\sigma_{a,Ret\_540}(\lambda_{exc})\phi_{iso,Ret\_540}\right) \tag{S14}$$

$$\frac{dN_{Ret\_540}}{dt_{exc}} = \frac{I_{exc}}{h\nu_{exc}}\left(N_{Ret\_580_I}\sigma_{a,Ret\_580}(\lambda_{exc})\phi_{iso,Ret\_580_I} - N_{Ret\_540}\sigma_{a,Ret\_540}(\lambda_{exc})\phi_{iso,Ret\_540}\right) \tag{S15}$$

with

$$N_{Ret\_540}(t_{exc}) = N_{Ret\_580_I,0} - N_{Ret\_580_I}(t_{exc}) \tag{S16}$$

where $N_{Ret\_580_I}$ is the number density of Ret_580I, N_{Ret_540} is the number density of Ret_540, and $N_{Ret\_580_I,0} = N_{Ret\_580_I}(0) = \kappa_{Ret\_580_I} N_{Ret\_580}(0)$ is the initial number density of Ret_580I at time $t_{exc} = 0$. $\sigma_{a,Ret\_580}(\lambda_{exc})$ and $\sigma_{a,Ret\_540}(\lambda_{exc})$ are the absorption cross-sections of Ret_580 and Ret_540 at the excitation wavelength λ_{exc}. The same absorption cross-section spectrum is assumed for Ret_580I and Ret_580II. σ_{a,Ret_580} is given in [33]. $\sigma_{a,Ret\_540,max}$ is set equal to $\sigma_{a,Ret\_580,max}$ and the shape of the absorption cross-section spectrum of Ret_540 is set equal to the shape of the absorption coefficient spectrum of Ret_580 (see for example Figures 1b, S1b, S4b, and S10b). I_{exc} is the excitation intensity. ϕ_{iso,Ret_580_I} is the quantum yield of photoisomerization of Ret_580I to Ret_540, and ϕ_{iso,Ret_540} is the quantum yield of photoisomerization of Ret_540 to Ret_580I (see Figures 6a and 7a).

Insertion of Equation (S16) into Equation (S14) and rearrangement of terms gives

$$\begin{aligned}&\frac{dN_{Ret\_580_I}}{dt_{exc}} + \frac{I_{exc}}{h\nu_{exc}}\left(\sigma_{a,Ret\_580}(\lambda_{exc})\phi_{iso,Ret\_580_I} + \sigma_{a,Ret\_540}(\lambda_{exc})\phi_{iso,Ret\_540}\right)N_{Ret\_580_I}\\ &= \frac{I_{exc}}{h\nu_{exc}} N_{Ret\_580_I,0}\sigma_{a,Ret\_540}(\lambda_{exc})\phi_{iso,Ret\_540}\end{aligned} \tag{S17}$$

Changing to the absorption coefficient development, $\alpha_{a,Ret\_580_I}(\lambda_{pr}) = N_{Ret\_580_I}\sigma_{a,Ret\_580}(\lambda_{pr})$, Equation (S17) changes to

$$\begin{aligned}&\frac{d\alpha_{Ret\_580_I}(\lambda_{pr})}{dt_{exc}} + \frac{I_{exc}}{h\nu_{exc}}\left(\sigma_{a,Ret\_580}(\lambda_{exc})\phi_{iso,Ret\_580_I} + \sigma_{a,Ret\_540}(\lambda_{exc})\phi_{iso,Ret\_540}\right)\alpha_{Ret\_580_I}(\lambda_{pr})\\ &= \frac{I_{exc}}{h\nu_{exc}} \alpha_{Ret\_580_I,0}(\lambda_{pr})\sigma_{a,Ret\_540}(\lambda_{exc})\phi_{iso,Ret\_540}\end{aligned} \tag{S18}$$

Equation (S18) is a linear differential equation with constant coefficients of the form

$$\frac{d\alpha_{Ret\_580_I}(\lambda_{pr})}{dt_{exc}} + f\alpha_{Ret\_580_I}(\lambda_{pr}) = g \tag{S19}$$

with

$$f = \frac{I_{exc}}{h\nu_{exc}}\left(\sigma_{a,\mathrm{Ret\_580}}(\lambda_{exc})\phi_{iso,\mathrm{Ret\_580_I}} + \sigma_{a,\mathrm{Ret\_540}}(\lambda_{exc})\phi_{iso,\mathrm{Ret\_540}}\right) \quad \text{(S20)}$$

$$g = \frac{I_{exc}}{h\nu_{exc}}\alpha_{\mathrm{Ret\_580_I},0}(\lambda_{pr})\sigma_{a,\mathrm{Ret\_540}}(\lambda_{exc})\phi_{iso,\mathrm{Ret\_540}} \quad \text{(S21)}$$

and the solution

$$\alpha_{\mathrm{Ret\_580_I}}(\lambda_{pr}, t_{exc}) = \alpha_{\mathrm{Ret\_580_I}}(\lambda_{pr}, 0)\exp(-ft_{exc}) + \frac{g}{f}\left[1-\exp(-ft_{exc})\right] \quad \text{(S22)}$$

The quantum yield of photoisomerization $\phi_{iso,\mathrm{Ret\_580_I}}$ of Ret_580I to Ret_540 is equal to the initial quantum yield of photoconversion $\phi_{con,\mathrm{Ret\_580_I},0} = \phi_{con,\mathrm{Ret\_580_I}}(t_{exc} \to 0)$ of Ret_580I determined in Section 2.2 (Figure 5). This equality is derived from Equations (1) to (4) with application of Equation (S14) for $t_{exc} \to 0$ (there $N_{\mathrm{Ret\_540}}(t_{exc} \to 0) = 0$). It gives

$$\phi_{con,\mathrm{Ret\_580_I},0} = -\frac{\Delta N_{\mathrm{Ret\_580_I}}(0)}{\Delta n_{ph,abs,I}(0)} = -\frac{\frac{dN_{\mathrm{Ret\_580_I}}(0)}{dt_{exc}}\delta t_{exc}}{\frac{I_{exc}\delta t_{exc}}{h\nu_{exc}}N_{\mathrm{Ret\_580_I}}(0)\sigma_{a,\mathrm{Ret\_580}}(\lambda_{exc})} = \frac{\frac{I_{exc}}{h\nu_{exc}}N_{\mathrm{Ret\_580_I}}(0)\sigma_{a,\mathrm{Ret\_580}}(\lambda_{exc})\phi_{\mathrm{iso,Ret\_580_I}}\delta t_{exc}}{\frac{I_{exc}\delta t_{exc}}{h\nu_{exc}}N_{\mathrm{Ret\_580_I}}(0)\sigma_{a,\mathrm{Ret\_580}}(\lambda_{exc})}$$
$$= \phi_{\mathrm{iso,Ret\_580_I}} \quad \text{(S23)}$$

Using the results obtained in Section 2.2, we obtain $\phi_{iso,\mathrm{Ret\_580_I}}$ (λ_{exc} = 590 nm) ≈ 0.056 and $\phi_{iso,\mathrm{Ret\_580_I}}$ (λ_{exc} = 530 nm) ≈ 0.023. These $\phi_{iso,\mathrm{Ret\_580_I}}$ results are included in Table 1.

The quantum yield of photoisomerization ϕ_{iso,Ret_540} of Ret_540 to Ret_580I is derived from Equation (S22) for $t_{exc} \to \infty$. It is

$$\alpha_{\mathrm{Ret\_580_I}}(\lambda_{pr}, \infty) = \frac{g}{f} = \frac{\alpha_{\mathrm{Ret\_580_I},0}(\lambda_{pr})\sigma_{a,\mathrm{Ret\_540}}(\lambda_{exc})\phi_{iso,\mathrm{Ret\_540}}}{\sigma_{a,\mathrm{Ret\_580}}(\lambda_{exc})\phi_{iso,\mathrm{Ret\_580_I}} + \sigma_{a,\mathrm{Ret\_540}}(\lambda_{exc})\phi_{iso,\mathrm{Ret\_540}}} \quad \text{(S24)}$$

Solving Equation (S24) for ϕ_{iso,Ret_540} gives

$$\phi_{iso,\mathrm{Ret\_540}} = \frac{\sigma_{a,\mathrm{Ret\_580}}(\lambda_{exc})\phi_{iso,\mathrm{Ret\_580_I}}}{\sigma_{a,\mathrm{Ret\_540}}(\lambda_{exc})\left(\frac{\alpha_{\mathrm{Ret\_580_I},0}(\lambda_{pr})}{\alpha_{\mathrm{Ret\_580_I}}(\lambda_{pr}, t_{exc} \to \infty)} - 1\right)} \quad \text{(S25)}$$

Insertion of values for λ_{exc} = 590 nm gives $\phi_{iso,ret\_540}(\lambda_{exc}$ = 590 nm) ≈ 0.21 (σ_{a,Ret_580}(590 nm) = 1.55 × 10^{-16} cm^2, $\phi_{iso,\mathrm{Ret\_580_I}}(590\ nm)$ = 0.056, σ_{a,Ret_540}(590 nm) = 6.91 × 10^{-17} cm^2, $\alpha_{\mathrm{Ret\_580_I},0}(\lambda_{pr} = 580\ nm)$ = 0.95 cm^{-1}, $\alpha_{\mathrm{Ret\_580_I}}(\lambda_{pr} = 580\ nm, t_{exc} \to \infty)$ = 0.595 cm^{-1}, top part of Figure S17), and $\phi_{iso,ret\_540}(\lambda_{exc}$ = 530 nm) ≈ 0.125 (σ_{a,Ret_580}(530 nm) = 1.0 × 10^{-16} cm^2, $\phi_{iso,\mathrm{Ret\_580_I}}(530\ nm)$ = 0.023, σ_{a,Ret_540}(530 nm) = 1.55 × 10^{-16} cm^2,

$\alpha_{\mathrm{Ret\_580_I},0}(\lambda_{pr} = 580\ nm)$ = 1.01 cm^{-1}, $\alpha_{\mathrm{Ret\_580_I}}(\lambda_{pr} = 580\ nm, t_{exc} \to \infty)$ = 0.902 cm^{-1}, bottom part of Figure S17). These $\phi_{iso,\mathrm{Ret\_540}}$ results are included in Table 1.

Ret_540 (PRSB$_{trans}$) relaxes to Ret_410 (RSB$_{trans}$) by proton release (Figures 6a and 7a) with the time constant $\tau_{\mathrm{rel,Ret\_540}}$. A value of $\tau_{\mathrm{rel,Ret\_540}}$ = 39 ± 3 s is extracted from the absorption coefficient recovery $\alpha_a(\lambda_{pr}$ = 530 nm, $t)$ of Figures 4 and S9 after excitation light switch-off. The value is included in Table 1.

The recovery of Ret_410 (RSB$_{trans}$) back to the original Ret_ 580$_I$ (PRSB$_{cis}$) by reprotonation and *trans-cis* isomerization with time constant $\tau_{rec,\mathrm{Ret\_410 \to Ret\_580_I}}$ occurs only partially with a quantum yield of recovery of $\phi_{rec,\mathrm{Ret\_410 \to Ret\_580_I}}$. The limited recovery is thought to be caused by a thermal Apoprotein$_I$ restructuring to irreversible Ret_400 [33] within the slow recovery of time constant $\tau_{rec,\mathrm{Ret\_410 \to Ret\_580_I}}$. The quantum yield of thermal conversion of Ret_410 to Ret_400 is $\phi_{therm,\mathrm{Ret\_410 \to Ret\_400}} = 1 - \phi_{rec,\mathrm{Ret\_410 \to Ret\_580_I}}$.

The parameters of $\tau_{rec,\mathrm{Ret\_410 \to Ret\_580_I}}$ and $\phi_{rec,\mathrm{Ret\_410 \to Ret\_580_I}}$ are extracted from the absorption coefficient development at λ_{pr} = 580 nm after excitation light switch-off. This development is given by

$$\alpha_a(t_{rec}) = \alpha_a(t_{exc,end}) + \Delta\alpha_{a,rec,\mathrm{Ret\_410 \to Ret\_580_I}}(t_{rec}) + \Delta\alpha_{a,rec,\mathrm{Ret\_370 \to Ret\_580_{II}}}(t_{rec}) \quad \text{(S26)}$$

The absorption coefficient recovery $\Delta\alpha_{a,rec,\mathrm{Ret\_410 \to Ret\text{-}580_{II}}}(t_{rec})$ at λ_{pr} = 580 nm due to back conversion of Ret_410 to Ret_580$_I$ is given by

$$\Delta\alpha_{a,rec,\mathrm{Ret\_410 \to Ret\_580_I}}(t_{rec}) = \Delta\alpha_{a,rec,\mathrm{Ret\_410 \to Ret\_580_{II}},0}\left[1 - \exp\left(-\frac{t_{rec}}{\tau_{rec,\mathrm{Ret\_410 \to Ret\_580_I}}}\right)\right] \quad \text{(S27)}$$

Values of $\Delta\alpha_{a,rec,\mathrm{Ret\_410 \to Ret\_580_I},0}$ and $\tau_{rec,\mathrm{Ret\_410 \to Ret\_580_I}}$ have been obtained by $\alpha_a(t_{rec},\lambda_{pr}$=580 nm) curve fits in the insets of Figure S8 for λ_{exc} = 530 nm, I_{exc} = 114.2 mW cm^{-2} ($\Delta\alpha_{a,rec,\mathrm{Ret\_410 \to Ret\_580_I},0}$ = $\Delta\alpha_{a,I}$(580 nm) = 0.238 cm^{-1}, $\tau_{rec,\mathrm{Ret\_410 \to Ret\_580_I}}$ = $\tau_{rec,I}$ = 0.87 h), and Figure S12 for λ_{exc} = 632.8 nm, I_{exc} = 15.65 mW cm^{-2} ($\Delta\alpha_{a,rec,\mathrm{Ret\_410 \to Ret\_580_I},0}$= $\Delta\alpha_{a,I}$(580 nm) = 0.312 cm^{-1}, $\tau_{rec,\mathrm{Ret\_410 \to Ret\_580_I}}$ = $\tau_{rec,I}$ = 2.56 h). The insets in Figures 3a and S3 for λ_{exc} = 590 nm cannot be used reliably because immediately after excitation light switch-off the samples were exposed for fluorescence measurements. The obtained absorption recovery time constants $\tau_{rec,\mathrm{Ret\_410 \to Ret\_580_I}}$ are included in Table 1. The recovery time for λ_{exc} = 530 nm is shorter than that of λ_{exc} = 632.8 nm probably due to the higher heat load to the sample by the shorter excitation wavelength and the higher excitation intensity.

The quantum yield of Ret_410 recovery to Ret_580$_I$, $\phi_{rec,\mathrm{Ret\_410 \to Ret\_580_I}}$, is approximately given by the ratio of recovered Ret_410 for $t_{rec} \to \infty$ to the photoexcited Ret_580$_I$. It is

$$\phi_{rec,\text{Ret\_410}\to\text{Ret\_580}_\text{I}} \approx \frac{\Delta\alpha_{a,rec,\text{Ret\_410}\to\text{Ret\_580}_\text{I},0}}{\kappa_{\text{Ret\_580}_\text{I}}\left[\alpha_a(t_{exc}=0)-\alpha_a(t_{exc,end})\right]} \quad \text{(S28)}$$

The obtained values are $\phi_{rec,\text{Ret\_410}\to\text{Ret\_580}_\text{I}} \approx 0.38$ for λ_{exc} = 632.8 nm, I_{exc} = 15.65 mW cm^{-2} (Figure S12, $\Delta\alpha_{a,rec,\text{Ret\_410}\to\text{Ret\_580}_\text{I},0} = \Delta\alpha_{a,I}$ = 0.312 cm^{-1}, $\kappa_{\text{Ret\_580}_\text{I}}$ = 0.41, $\alpha_a(t_{exc}=0)$ = 2.45 cm^{-1}, $\alpha_a(t_{exc,end})$ = 0.452 cm^{-1}) and $\phi_{rec,\text{Ret\_410}\to\text{Ret\_580}_\text{I}} \approx 0.42$ for λ_{exc} = 530 nm, I_{exc} = 114.2 mW cm^{-2} (Figure S8, $\Delta\alpha_{a,rec,\text{Ret\_410}\to\text{Ret\_580}_\text{I},0} = \Delta\alpha_{a,I}$ = 0.238 cm^{-1}, $\kappa_{\text{Ret\_580}_\text{I}}$ = 0.41, $\alpha_a(t_{exc}=0)$ = 2.40 cm^{-1}, and $\alpha_a(t_{exc,end})$ = 1.02 cm^{-1}). These data are included in Table 1.

The quantum yield of thermal conversion of Ret_410 to Ret_400, $\phi_{therrm,\text{Ret\_410}\to\text{Ret\_400}}$, is given by

$$\phi_{therm,\text{Ret\_410}\to\text{Ret\_400}} = 1-\phi_{rec,\text{Ret\_410}\to\text{Ret\_580}_\text{I}} \quad \text{(S29)}$$

The obtained values are $\phi_{therm,\text{Ret\_410}\to\text{Ret\_400}} \approx 0.62$ for λ_{exc} = 632.8 nm and I_{exc} = 15.65 mW cm^{-2} (Figure S12) and $\phi_{therm,\text{Ret\_410}\to\text{Ret\_400}} \approx 0.58$ for λ_{exc} = 530 nm and I_{exc} = 114.2 mW cm^{-2} (Figure S8). These data are included in Table 1.

S4.2 Photocycle Dynamics Calculations for Ret_580$_{II}$

For $t_{exc} > \tau_{rel,\text{Ret\_640}} \approx 17$ s and $t_{exc} \ll \tau_{rec,\text{Ret\_410}\to\text{Ret\_580}_\text{I}} \approx 1.5$ h the photoexcitation dynamics of Ret_580$_{II}$ is given by

$$\frac{dN_{\text{Ret\_580}_\text{II}}}{dt_{exc}} = \frac{I_{exc}}{h\nu_{exc}}\left(-N_{\text{Ret\_580}_\text{II}}\sigma_{\text{Ret\_580}}(\lambda_{exc})\phi_{iso,\text{Ret\_580}_\text{II}} + N_{\text{Ret\_640}}\sigma_{\text{Ret\_640}}(\lambda_{exc})\phi_{iso,\text{Ret\_640}}\right) \quad \text{(S30)}$$

$$\frac{dN_{\text{Ret\_640}}}{dt_{exc}} = \frac{I_{exc}}{h\nu_{exc}}\left(N_{\text{Ret\_580}_\text{II}}\sigma_{\text{Ret\_580}}(\lambda_{exc})\phi_{iso,\text{Ret\_580}_\text{II}} - N_{\text{Ret\_640}}\sigma_{\text{Ret\_640}}(\lambda_{exc})\phi_{iso,\text{Ret\_640}}\right) - \frac{N_{\text{Ret\_640}}}{\tau_{\text{rel,Ret\_640}}} \quad \text{(S31)}$$

The steady state situation $\frac{dN_{\text{Ret\_640}}}{dt_{exc}} = 0$ for $t_{exc} > \tau_{rel,\text{Ret\_640}}$ gives

$$N_{\text{Ret\_640}}(t_{exc}) = \frac{\frac{I_{exc}}{h\nu_{exc}}N_{\text{Ret\_580}_\text{II}}\sigma_{\text{Ret\_580}}(\lambda_{exc})\phi_{iso,\text{Ret\_580}_\text{II}}}{\frac{1}{\tau_{rel,\text{Ret\_640}}} + \frac{I_{exc}}{h\nu_{exc}}\sigma_{\text{Ret\_640}}(\lambda_{exc})\phi_{iso,\text{Ret\_640}}} \quad \text{(S32)}$$

Insertion of Equation (S32) into Equation (S30) leads to

$$\frac{dN_{\mathrm{Ret\_580_{II}}}}{dt_{exc}}=\frac{I_{exc}}{h\nu_{exc}}\left(-N_{\mathrm{Ret\_580_{II}}}\sigma_{\mathrm{Ret\_580}}(\lambda_{exc})\phi_{iso,\mathrm{Ret\_580_{II}}}+\frac{\frac{I_{exc}}{h\nu_{exc}}N_{\mathrm{Ret\_580_{II}}}\sigma_{\mathrm{Ret\_580}}(\lambda_{exc})\phi_{iso,\mathrm{Ret\_580_{II}}}}{\frac{1}{\tau_{rel,\mathrm{Ret\_640}}}+\frac{I_{exc}}{h\nu_{exc}}\sigma_{\mathrm{Ret\_640}}(\lambda_{exc})\phi_{iso,\mathrm{Ret\_640}}}\sigma_{\mathrm{Ret\_640}}(\lambda_{exc})\phi_{iso,\mathrm{Ret\_640}}\right) \quad \text{(S33)}$$

The quantum yield of photoconversion $\phi_{con,\mathrm{Ret\_580_{II}}}$ of Section 2.2 (Equations (1)–(4)) applied to Ret_580II may be expressed as

$$\phi_{con,\mathrm{Ret\_580_{II}}}=\frac{-\Delta N_{\mathrm{Ret\_580_{II}}}}{\Delta n_{ph,abs}}=\frac{-\frac{dN_{\mathrm{Ret\_580_{II}}}}{dt}\delta t_{exc}}{\frac{I_{exc}\delta t_{exc}}{h\nu_{exc}}N_{\mathrm{Ret\_580_{II}}}\sigma_{a,\mathrm{Ret\_580}}(\lambda_{exc})}=\frac{-\frac{dN_{\mathrm{Ret\_580_{II}}}}{dt}}{\frac{I_{exc}}{h\nu_{exc}}N_{\mathrm{Ret\_580_{II}}}\sigma_{a,\mathrm{Ret\_580}}(\lambda_{exc})} \quad \text{(S34)}$$

Insertion of Equation (S33) into Equation (S34) gives

$$\phi_{con,\mathrm{Ret\_580_{II}}}=\phi_{iso,\mathrm{Ret\_580_{II}}}-\frac{\frac{I_{exc}}{h\nu_{exc}}\phi_{iso,\mathrm{Ret\_580_{II}}}}{\frac{1}{\tau_{rel,\mathrm{Ret\_640}}}+\frac{I_{exc}}{h\nu_{exc}}\sigma_{\mathrm{Ret\_640}}(\lambda_{exc})\phi_{iso,\mathrm{Ret\_640}}}\sigma_{\mathrm{Ret\_640}}(\lambda_{exc})\phi_{iso,\mathrm{Ret\_640}} \quad \text{(S35)}$$

The quantum yield of photoisomerization of Ret_580II, $\phi_{iso,\mathrm{Ret\_580_{II}}}$, is obtained from Equation (S35) for $I_{exc} \to 0$. That is

$$\phi_{iso,\mathrm{Ret\_580_{II}}}=\phi_{con,\mathrm{Ret\_580_{II}}}(I_{exc}\to 0) \quad \text{(S36)}$$

Its value is obtained from the top part of Figure 5 to be $\phi_{iso,\mathrm{Ret\_580_{II}}}(\lambda_{exc}=590\ nm)$ = (1.35±0.15) × 10^{-3}.

Solving of Equation (S35) to $\phi_{iso,\mathrm{Ret\_640}}$ gives

$$\phi_{iso,\mathrm{Ret\_640}}=\frac{\frac{\phi_{iso,\mathrm{Ret\_580_{II}}}}{\phi_{con,\mathrm{Ret\_580_{II}}}(I_{exc})}-1}{\frac{I_{exc}}{h\nu_{exc}}\sigma_{a,\mathrm{Ret\_640}}(\lambda_{exc})\tau_{\mathrm{rel,Ret\_640}}} \quad \text{(S37)}$$

Insertion of parameters for λ_{exc} = 590 nm and I_{exc} = 64.65 mW cm^{-2} gives $\phi_{iso,\mathrm{Ret\_640}}$ ≈ 0.12 ($\phi_{iso,\mathrm{Ret\_580_{II}}}$ ≈ 0.00135, $\phi_{con,\mathrm{Ret\_580_{II}}}(I_{exc}=64.65\ \mathrm{mW\ cm^{-2}})$ ≈ 4.53 × 10^{-5}, $\sigma_{a,\mathrm{Ret\_640}}$(590 nm) ≈ 7.5 × 10^{-17} cm^2, and $\tau_{\mathrm{rel,Ret\_640}}$ ≈ 17 s). This result is included in Table 1.

Ret_640 (PRSB$_{cis}$) relaxes to Ret_370 (RSB$_{cis}$) by proton release (Figures 6b and 7b) with the time constant $\tau_{\mathrm{rel,Ret\_640}}$. A value of $\tau_{\mathrm{rel,Ret\_640}}$ = 17 ± 3 s is extracted from the absorption coefficient recovery $\alpha_a(\lambda_{pr}$ = 370 nm, $t)$ of Figures 4 and S9 after excitation light switch-off.

The recovery of Ret_370 (RSB$_{cis}$) back to the original Ret_ 580II (PRSB$_{trans}$) by reprotonation and *cis-trans* isomerization with time constant $\tau_{rec,\mathrm{Ret\_370\to Ret\_580_{II}}}$ occurs only partially with a quantum yield of recovery of $\phi_{rec,\mathrm{Ret\_370\to Ret\_580_{II}}}$. This limited recovery is thought to be caused by a thermal

Apoprotein$_{II}$ restructuring to irreversible Ret_350 [33] within the slow recovery of time constant $\tau_{rec,\mathrm{Ret\_370}\rightarrow\mathrm{Ret\_580_{II}}}$. The quantum yield of thermal conversion of Ret_370 to Ret_350 is $\phi_{therm,\mathrm{Ret\_370}\rightarrow\mathrm{Ret\_350}} = 1 - \phi_{rec,\mathrm{Ret\_370}\rightarrow\mathrm{Ret\_580_{II}}}$.

The parameters of $\tau_{rec,\mathrm{Ret\_370}\rightarrow\mathrm{Ret\_580_{II}}}$ and $\phi_{rec,\mathrm{Ret\_370}\rightarrow\mathrm{Ret\_580_{II}}}$ are extracted from the absorption coefficient development at λ_{pr} = 580 nm after excitation light switch-off (Equation (S26)). The absorption coefficient recovery $\Delta\alpha_{a,rec,\mathrm{Ret\_370}\rightarrow\mathrm{Ret\_580_{II}}}$ at λ_{pr} = 580 nm due to back conversion of Ret_370 to Ret_580$_{II}$ is given by

$$\Delta\alpha_{a,rec,\mathrm{Ret\_370}\rightarrow\mathrm{Ret\_580_{II}}}(t_{rec}) = \Delta\alpha_{a,rec,\mathrm{Ret\_370}\rightarrow\mathrm{Ret\_580_{II}},0}\left[1-\exp\left(-\frac{t_{rec}}{\tau_{rec,\mathrm{Ret\_370}\rightarrow\mathrm{Ret\_580_{II}}}}\right)\right] \quad \text{(S38)}$$

Values of $\Delta\alpha_{a,rec,\mathrm{Ret\_370}\rightarrow\mathrm{Ret\_580_{II}},0}$ and $\tau_{rec,\mathrm{Ret\_370}\rightarrow\mathrm{Ret\_580_{II}}}$ have been obtained by $\alpha_a(t_{rec},\lambda_{pr}$=580 nm) curve fits in the insets of Figure S8 for λ_{exc} = 530 nm and I_{exc} = 114.2 mW cm^{-2} ($\Delta\alpha_{a,rec,\mathrm{Ret\_370}\rightarrow\mathrm{Ret\_580_{II}},0}$ = $\Delta\alpha_{a,II}$(580 nm) = 0.525 cm^{-1}, $\tau_{rec,\mathrm{Ret\_370}\rightarrow\mathrm{Ret\_580_{II}}}$ = $\tau_{rec,I}$ = 7.81 h) and Figure S12 for λ_{exc} = 632.8 nm and I_{exc} = 15.65 mW cm^{-2} ($\Delta\alpha_{a,rec,\mathrm{Ret\_370}\rightarrow\mathrm{Ret\_580_{II}},0}$ = $\Delta\alpha_{a,II}$(580 nm) = 0.505 cm^{-1}, $\tau_{rec,\mathrm{Ret\_370}\rightarrow\mathrm{Ret\_580_{II}}}$ = $\tau_{rec,II}$ = 14.7 h). The obtained absorption recovery time constants $\tau_{rec,\mathrm{Ret\_370}\rightarrow\mathrm{Ret\_580_{II}}}$ are included in Table 1. The recovery time constant for λ_{exc} = 530 nm is shorter than that of λ_{exc} = 632.8 nm, probably due the higher heat load to the sample by the shorter excitation wavelength and the higher excitation intensity.

The quantum yield of Ret_370 recovery to Ret_580$_{II}$, $\phi_{rec,\mathrm{Ret\_370}\rightarrow\mathrm{Ret\_580_{II}}}$, is approximately given by the ratio of recovered Ret_370 to Ret_580$_{II}$ for $t_{rec} \rightarrow \infty$ to the photoexcited Ret_580$_{II}$. It is

$$\phi_{rec,\mathrm{Ret\_370}\rightarrow\mathrm{Ret\_580_{II}}} \approx \frac{\Delta\alpha_{a,rec,\mathrm{Ret\_370}\rightarrow\mathrm{Ret\_580_{II}},0}}{\kappa_{\mathrm{Ret\_580_{II}}}\left[\alpha_a(t_{exc}=0)-\alpha_a(t_{exc,end})\right]} \quad \text{(S39)}$$

The obtained values are $\phi_{rec,\mathrm{Ret\_370}\rightarrow\mathrm{Ret\_580_{II}}}$ ≈ 0.43 for λ_{exc} = 632.8 nm and I_{exc} = 15.65 mW cm^{-2} (Figure S12) and $\phi_{rec,\mathrm{Ret\_370}\rightarrow\mathrm{Ret\_580_{II}}}$ ≈ 0.64 for λ_{exc} = 530 nm and I_{exc} = 114.2 mW cm^{-2} (Figure S8). These data are included in Table 1.

The quantum yield of thermal conversion of Ret_370 to Ret_350, $\phi_{therm,\mathrm{Ret\_370}\rightarrow\mathrm{Ret\_350}}$, is given by

$$\phi_{therm,\mathrm{Ret\_370}\rightarrow\mathrm{Ret\_350}} = 1-\phi_{rec,\mathrm{Ret\_370}\rightarrow\mathrm{Ret\_580_{II}}} \quad \text{(S40)}$$

The obtained values are $\phi_{therm,\mathrm{Ret\_370 \rightarrow Ret\_350}}$ ≈ 0.57 for λ_{exc} = 632.8 nm and I_{exc} = 15.65 mW cm^{-2} (Figure S12) and $\phi_{therm,\mathrm{Ret\_370 \rightarrow Ret\_350}}$ ≈ 0.36 for λ_{exc} = 530 nm and I_{exc} = 114.2 mW cm^{-2} (Figure S8). These data are included in Table 1.

S5. Quantum Yields of Primary Photoisomerizations of some Rhodopsins

In Table S1, first-step photoisomerization quantum yields of some microbial rhodopsins (type I rhodopsins) and an animal rhodopsin (type II rhodopsin) are listed.

Table S1. Quantum yield of photoisomerization comparison of rhodopsins.

Name	Buffer	Cofactor	Primary photoisomer	ϕ_{iso}	Reference
Rh		$PRSB_{11\text{-}cis}$	$PRSB_{all\text{-}trans}$	0.67	[56,57]
PR	pH 8	$PRSB_{all\text{-}trans}$	$PRSB_{13\text{-}cis}$	≈0.7	[58]
BR	pH 7.5	$PRSB_{all\text{-}trans}$	$PRSB_{13\text{-}cis}$	0.65	[59,60]
HR	pH 7	$PRSB_{all\text{-}trans}$	$PRSB_{13\text{-}cis}$	0.34	[61]
pSRII	pH 8	$PRSB_{all\text{-}trans}$	$PRSB_{13\text{-}cis}$	0.5	[62]
C1C2	pH 8	$PRSB_{all\text{-}trans}$	$PRSB_{13\text{-}cis}$	0.30	[63]
ASR	pH 7.5	$PRSB_{all\text{-}trans}$	$PRSB_{13\text{-}cis}$	≈0.2	[64]
ASR	pH 7.5	$PRSB_{13\text{-}cis}$	$PRSB_{all\text{-}trans}$	≈0.4	[64]
HKR1	pH 7.4	$RSB_{13\text{-}cis}$	$RSB_{all\text{-}trans}$	0.096	[65]
HKR1	pH 7.4	$PRSB_{all\text{-}trans}$	$PRSB_{13\text{-}cis}$	0.405	[65]
BeRh	pH 8	$PRSB_{all\text{-}trans}$	$PRSB_{13\text{-}cis}$	0.66	[66]
CaRh	pH 7.3	$PRSB_{all\text{-}trans}$	$PRSB_{13\text{-}cis}$	0.46	[67]
QuasAr1	pH 8	$PRSB_{cis}$ (Ret_580$_{I}$)	$PRSB_{trans}$	0.056	This work
QuasAr1	pH 8	$PRSB_{trans}$ (Ret_580$_{II}$)	$PRSB_{cis}$	0.00135	This work

Abbreviations: Rh, bovine rhodopsin (type II rhodopsin); PR, proteorhodopsin from uncultivated marine γ-proteobacteria; BR, bacteriorhodopsin from *Halobacterium salinarum*; HR, halorhodopsin from *Halobacterium halobium*; pSRII, sensory rhodopsin II from *Natronobacterium pharaonic*; C1C2, chimeric channelrhodopsin ChR1 and ChR2 from *Chlamydomonas reinhardtii*; ASR, sensory rhodopsin from *Anabaena* (*Nostoc*) sp. PCC 7120 cyanobacterium; PRSB, protonated retinal Schiff base; RSB, deprotonated retinal Schiff base; ϕ_{iso}, quantum yield of photoisomerization.

References

47. Malencik, D.A.; Sprouse, J.F.; Swanson, C.A.; Anderson, S.R. Dityrosine: Preparation, isolation, and analysis. *Anal. Biochem.* **1996**, *242*, 202–213.
48. Bent, D.V.; Hayon, E. Excited state chemistry of aromatic amino acids and related peptides. I. Tyrosine. *J. Am. Chem. Soc.* **1975**, *97*, 2599–2606.
49. Jovanovic, S.V.; Simic, M.G. Repair of tryptophan radicals by antioxidants. *J. Free Radic. Biol. Med.* **1985**, *1*, 125–129.
50. Holzer, W.; Pichlmaier, M.; Penzkofer, A.; Bradley, D.D.C.; Blau, W.J. Fluorescence spectroscopic behaviour of neat and blended conjugated polymer thin films. *Chem. Phys.* **1999**, *246*, 445–462.
51. Förster, T. *Fluoreszenz Organischer Verbindungen*; Vandenhoeck und Ruprecht: Göttingen, Germany, 1951; pp. 83–86.
52. Valeur, B.; Berberan-Santos, M.N. *Molecular Fluorescence: Principles and Applications*, 2nd ed.; Wiley-VCH: Weinheim, Germany, 2012; pp. 213–261.
53. Birkmann, C.; Penzkofer, A.; Tsuboi, T. Fluorescence excitation spectroscopic characterization of colour centres in a LiF crystal. *Appl. Phys. B* **2003**, *77*, 625–632.
54. Penzkofer, A. Passive Q-switching and mode-locking for the generation of nanosecond to femtosecond pulses. *Appl. Phys. B* **1988**, *46*, 43–60.

55. Bansal, A.K.; Penzkofer, A. Spectroscopic and travelling-wave lasing characterisation of tetraphenylbenzidine and di-naphtalenyl-diphenylbenzidine. *Appl. Phys. B* **2008**, *91*, 559–569.
56. Dartnall, H.J.A. The photosensitivities of visual pigments in the presence of hydroxylamine. *Vis. Res.* **1967**, *8*, 339–358.
57. Kandori, H.; Shichida, Y.; Yoshizawa, T. Photoisomerization in rhodopsin. *Biochemistry (Moscow)* **2001**, *66*, 339–358.
58. Rupenyan, A.; van Stokkum, I.H.M.; Arents, J.C.; van Grondelle, R.; Hellingwerf, K.; Groot, M.L. Characterization of the primary photochemistry of proteorhodopsin with femtosecond spectroscopy. *Biophys. J.* **2008**, *94*, 4020–4030.
59. Tittor, J.; Oesterhelt D. The quantum yield of bacteriorhodopsin. *FEBS Lett.* **1990**, *263*, 269–273.
60. Logunov, S.L.; El-Sayed M.A. Redetermination of the quantum yield of photoisomerization and energy content in the K-intermediate of bacteriorhodopsin photocycle and its mutants by the photoacoustic technique. *J. Phys. Chem. B* **1997**, *101*, 6629–6633.
61. Oesterhelt, D.; Hegemann, P.; Tittor, J. The photocycle of the chloride pump halorhodopsin. II: Quantum yields and a kinetic model. *EMBO J.* **1985**, *4*, 2351–2356.
62. Losi, A.; Wegener, A.A.; Engelhard, M.; Gärtner, W.; Braslavsky, S.E. Time-resolved absorption and photothermal measurements with recombinant sensory rhodopsin II from natronobacterium *Pharaonis*. *Biophys. J.* **1999**, *77*, 3277–3286.
63. Hontani, Y.; Marazzi, M.; Stehfest, K.; Mathes, T.; van Stokkum, I.H.M.; Elstner, M.; Hegemann, P.; Kennis, J.T.M. Reaction dynamics of the chimeric channelrhodopsin C1C2. *Sci. Rep.* **2017**, *7*, 7217.
64. Wand, A.; Rozin, R.; Eliash, T.; Jung, K.-H.; Sheves, M.; Ruhman, V. Asymmetric toggling of a natural photoswitch: Ultrafast spectroscopy of *Anabaena* sensory rhodopsin. *J. Am. Chem. Soc.* **2011**, *133*, 20922–20932.
65. Penzkofer, A.; Luck, M.; Mathes, T.; Hegemann, P. Bistable retinal Schiff base photodynamics of histidine kinase rhodopsin HKR1 from *Chlamydomonas reinhardtii*. *Photochem. Photobiol.* **2014**, *90*, 773–785.
66. Penzkofer, A.; Scheib, U.; Hegemann, P.; Stehfest, K. Absorption and emission spectroscopic investigation of thermal dynamics and photo-dynamics of the rhodopsin domain of the rhodopsin-guanylyl cyclase from the aquatic fungus *Blastocladiella emersonii*. *BAOJ Phys.* **2016**, *2*, 1–22.
67. Penzkofer, A.; Scheib, U.; Stehfest, K.; Hegemann, P. Absorption and emission spectroscopic investigation of thermal dynamics and photo-dynamics of the rhodopsin domain of the rhodopsin-guanylyl cyclase from the nematophagous fungus *Catenaria anguillulae*. *Int. J. Mol. Sci.* **2017**, *18*, 2099.
